# Supplementary material for: Impact of SARS-CoV-2 infection and mitigation strategy during pregnancy on prenatal outcome, growth and development in early childhood in India: a UKRI GCRF Action Against Stunting Hub protocol paper
Source: BMJ Paediatr Open. 2024 Feb 27;8(Suppl 1):e001900. doi: 10.1136/bmjpo-2023-001900 (PMC10900341; doi:10.1136/bmjpo-2023-001900)
Supplement: Supplementary data [file bmjpo-2023-001900supp001.pdf]

Longitudinal follow-up of babies born to covid-19 positive antenatal mothers in Gujarat -Maternal

Basic Details

Question	Responses	Remarks
Name of District	1. Ahmedabad<br>**2.** Sabarkantha	
Mother UID	☐☐ ☐☐☐ ☐☐	
Status of Covid infection during Pregnancy	0. Negative<br>1. Positive	
Date of Interview		
GPS location		
Name of data collector:		

Section 1: Socio-demographic details

No	Question	Response	Instruction
1	Mother's s Birth date	DOB_________	Record verbal age if participants do not remember the birth date
2	Father's s Birth date	DOB_________	Record verbal age if participants do not remember the birth date
3	Religion	1. Hindu<br>2. Islam<br>3. Christian<br>4. Others _____________	
4	Caste	1. SC<br>2. ST<br>3. OBC<br>4. General	
5	Education	1. Illiterate<br>2. Can read and write<br>3. Primary<br>4. Secondary<br>5. Higher Secondary<br>6. Graduation<br>7. Postgraduation& above	
6	What type of Ration card do you have?	1. APL (white)<br>2. BPL (yellow)<br>3. Not any<br>4. Not aware/don't know	
7	Occupation	1. Farmer<br>2. Labourer<br>3. Business<br>4. Job<br>5. Housewife<br>6. Any other	
8	What is your family type?	1. Joint<br>2. Nuclear	
9	Total Number of family members in HH (including you)	.	

No	Question	Response	Instruction
10	List all family members with their age and relation to participants in detail.	________________________	
11	Status of current Housing	1. Own<br>2. Rented<br>3. Living with relatives/friends<br>4. Other	
12	Type of house (current)	0. No House/rented<br>1. Hut<br>2. Kuccha House (walls/floors made from mud, roof made from leaves or other low-quality materials)<br>3. Semi-pucca House (Partly low quality and partly high-quality material used)<br>4. Pucca House (High-quality materials used including roof, walls, floor)<br>5. Mansion	
13	Was the status and type of housing being same during covid pandemic?	0. No<br>1. Yes	*If yes, jump to the Q:15*
14	If no, record the difference in terms of both (Type & status)	______________________________	
15	Household fuel source (current)	1. Electricity (Government/state grid)<br>2. Generator (Public/personal)<br>3. Kerosine<br>4. Others (specify)	
16	Was the HH fuel source was same during covid pandemic?	0. No<br>1. Yes	*If yes, jump to the Q:18*
17	If No, record the difference in details.	__________________________________	
18	Cooking fuel source (current)	1. LPG<br>2. Wood/straw<br>3. Animal waste<br>4. Coal/Charcoal<br>5. Kerosine<br>6. Others (specify)	
19	Was the Cooking fuel source being same during the pandemic?	0. No<br>1. Yes	*If yes, jump to the Q:14*
20	If no, record the difference in details.	______________________	
21	Material Possession	1. Livestock (Cow, Sheep, Chicken, etc.)<br>2. Fridge<br>3. TV<br>4. Vehicle (car, motorcycle, etc.)<br>5. Broadband internet<br>6. Others_____<br>7. Not any_____	
22	What is the primary source of water for your Household usage?	1. Piped water into dwelling<br>2. Piped water into the yard/plot<br>3. Piped to neighbour<br>4. Public-tap/standpipe	

No	Question	Response	Instruction
		5. Tube-well/borehole<br>6. Dug well<br>7. Water from spring<br>8. Rain water<br>9. Water-vendor -tanker truck/cart delivery<br>10. Open water source (e.g. River, lake, stream, canal)<br>11. Other (specify)	
23	Do you have the same source of drinking water as HH?	0. No<br>1. Yes	*If yes, jump to the Q:25*
24	If not the same, please specify. (Write option number from Q:22)		
25	Do you have to walk/travel to get household/drinking water?	0. No<br>1. Yes	*If NO, jump to the Q:27*
26	If Yes, Distance travelled the closest source of water?		
27	Do you have a toilet facility in your house?	0. No<br>1. Yes	*If NO, jump to the Q:29*
28	If yes, what type of toilet	1. Toilet with water flush or pour-flush<br>2. Ventilated improved pit (VIP) latrine<br>3. Pit latrine with slab<br>4. Pit latrine without slab/open pit<br>5. Twin pit/composting toilet	
29	Since how long are you staying in this house with these amenities?	No of months________	
30	Were these house amenities being same (water source + Toilet facilities _ material passion) during the pandemic?	0. No<br>1. Yes	*If yes, end the Section here*
31	If no, record the details for each of the three parameters	___________________________	

Section 2: Household Income details

No	Question	Response	Instruction
32	Are you involved in any paid work?	0. No<br>1. Yes	*If no jump to the Q:38*
33	if yes, state the type of work.	_________	
34	Do you commute to work?	0. No<br>1. Yes	*If no, jump to the Q:36.*
35	If yes, state the travel distance and the transportation type used.	_______________	
36	How much are you paid per month? State the amount, if possible. If not, please give us a close estimation.		
37	Since how many years have you been involved with this work?		
38	Is your husband involved in any paid work?	0. No<br>1. Yes	*If no, jump to the Q:44.*
39	If yes, state the type of work.	__________	
40	Does he commute to work?	0. No<br>1. Yes	*If no, jump to the Q:42.*

No	Question	Response	Instruction
41	If yes, state the distance of travel and the type of transportation used.		
42	How much does he earn per month? State the amount if possible. If not, please give an estimation.		Please use 9 if the information is not known or refused.
43	Since how many years has, he been involved in this same job with the same travelling options?		
44	Does anyone else work in your household?	0. No<br>1. Yes	*If No, end sect. 2 here*
45	If yes, state your relationship (daughter, son, cousin, uncle, etc.) and the type of work.	1. Type of work________<br>2. Relationship with you_____	
46	Do they commute to work?	0. No<br>1. Yes	
47	If yes, state distance of travel and type of transportation.		
48	How much do they earn? State the amount if possible. If not please estimate.		Please use 9 in the data entry if the information is not known/refused.
49	How much have your total household income each month? State amount if known.		If not please estimate. Please use 9 in the data entry if the information is refused.
50	Do you have other sources of income (such as gifts, remittance, inheritance, etc.)	0. No<br>1. Yes	
51	Does the covid-19 pandemic affect your household income?	0. No<br>1. Yes	
52	Whose income was affected in the household?	1. Self<br>2. Husband<br>3. Son<br>4. Daughter<br>5. Uncle<br>6. Any other	
53	How was the income affected?	1. Lost job totally<br>2. lost some earnings<br>3. Other	
54	On average, how much earnings would you say your household has lost during the pandemic? State amount. If not, please estimate.		

Section 3: Current anthropometry, morbidity and addiction details

No	Question	Response	Instruction
55	Are you currently pregnant?	0. No<br>1. Yes	
56	Are you currently breastfeeding?	0. No<br>1. Yes	
57	Are you suffering from any of the listed diseases? (current)	1. Not any<br>2. Diabetes<br>3. Hypertension	Multiple answers possible

No	Question	Response	Instruction
		4. Cardiac disease<br>5. Thyroid<br>6. Chronic respiratory diseases<br>7. Immune-compromised status<br>8. Any other_______	
58	Were you suffering from any of the listed diseases during your pregnancy (please consider the pregnancy during the pandemic)?	1. Not any<br>2. Diabetes<br>3. Hypertension<br>4. Cardiac disease<br>5. Thyroid<br>6. Chronic respiratory diseases<br>7. Immune-compromised status<br>8. Any other___	Multiple answers possible – please consider the during the pandemic pregnancy
59	Do you have any infection/illness within the past seven days?	0. No<br>1. Yes	
60	If yes, record the details.	_________________	
61	Do you currently have any addiction to any of the listed items?	1. Not any<br>2. Cigarette/bidi smoking<br>3. Tobacco chewing<br>4. Alcohol<br>5. Any other	
62	If any other, record the details about the item's name with frequency/day.	__________	
63	If the response is except "not any", then record the frequency/day for each type of received response.	_________	
64	Did you have any addiction during pregnancy from the listed items?	1. Not any<br>2. Cigarette/bidi smoking<br>3. Tobacco chewing<br>4. Alcohol<br>5. Any other	
65	If any other, record the details about the item's name with frequency/day.		
66	If the response is except "not any", then record the frequency/day for each type of received response.		
67	Any other history of hospitalization (mother) till date?	1. No<br>2. Yes	
68	If yes, record the reason & no of days		

No	Question	Response	Instruction
69	Weight in kg during pregnancy (Recorded)	________________kg	
70	Level of Hb during pregnancy (Recorded)		
71	Level of RBS during pregnancy (Recorded)		
72	BP during pregnancy (Recorded)		
73	Maternal current weight in kg		
74	Weight on the date of the interview		Current anthropometry will be taken on every follow-up visits
75	Height in cm	________________cm	
76	Maternal MUAC on the date of the interview	________________cm	
77	Skin fold thickness – Triceps on the date of the interview	________________mm	
78	Skin fold thickness - Biceps on the date of the interview	________________mm	
79	Skin fold thickness – Scapula on the date of the interview	________________mm	
80	Skin fold thickness – Supra iliac on the date of the interview	________________mm	
81	Waist circumference (in cm) on the date of the interview	________________cm	
82	Hip Circumference (in cm) on the date of the interview	________________cm	

Section 4: Covid Infection Prevention and vaccination details

No	Question	Response	Instruction
83	Have you followed any of the following covid-infection preventive measures strictly and regularly?	1. Face mask wearing<br>2. Handwashing<br>3. Use of hand sanitizer frequently<br>4. Social distancing (Avoiding all general social gatherings)<br>5. Staying away from infected or suspected patients<br>6. Not any<br>7. Any other	*Multi response.*<br>*If Ans: is not any, then jump to 85*
84	If yes, specify the duration	1. During all three waves of pandemic<br>2. During Covid-infection only<br>3. Only if anyone infected in family/close contact<br>4. Not Followed	
85	Have you taken any of the following Prophylactic drugs and dietary supplements to prevent from Covid pandemic	1. Vitamin C<br>2. Vitamin D<br>3. Zink<br>4. Ayurvedic homemade Gadha<br>5. All of the above<br>6. Not any<br>7. Others	*Multi response.*<br>*If Ans: is not any, then jump to 87*
86	If yes, specify the duration (no of weeks) and Dosage.		
87	Status of during pregnancy	1. Not vaccinated<br>2. One dose<br>3. Two doses<br>4. Two doses + precautionary	

88	Status of covid vaccination on the date of interview (current)	5. Not vaccinated<br>6. One dose<br>7. Two doses<br>8. Two doses + precautionary	
89	Date & Type of the first vaccine	1. Date_____<br>2. Type_____	
90	Date & Type of the second vaccine	1. Date_____<br>2. Type______	
91	Date & Type of Precautionary Vaccine	1. Date______<br>2. Type______	
92	Did you receive covid-19 counselling during pregnancy?	1. No<br>2. Yes<br>3. Not applicable (no guideline introduced at that time)	*If Ans: is no any, then end the sec:3 here*
93	if yes, where did you receive the counselling?	1. Any Hospital settings (PHC/CHC/SC/private clinic)<br>2. Mamta day<br>3. Mass media (radio, tv,)<br>4. Family/friends<br>5. Other (write)	
94	If yes, did the counselling help you to understand the covid-19 disease, vaccination, misinformation, and its impact on your pregnancy and baby?	1. Yes – It was helped me to develop good understanding<br>2. yes – in somewhat aspects<br>3. yes – little bit but still I have/had many doubts<br>4. Others	

Section 5: Status of Covid Infection during pregnancy and Management details

No	Question	Response	Instruction
95	Have you been tested positive for COVID 19 during your pregnancy?	0. No<br>1. Yes	
96	If yes, during which wave it was detected?	1. 1.1st wave- (March 2020 to November 2020)<br>2. 2.2nd wave- (February 2021 to June 2021)<br>3. 3.3rd wave- (November 2021 to March 2022)	Skip Q:96 to 117 if the answer of ques. 95 is No
97	If yes, record the performed confirmatory investigations and result	Test done / Result (0/1) / Date<br>RAT<br>RTPCR<br>others	
98	If yes, in which trimester of pregnancy?	______month	The First Trimester – (0-13 weeks)<br>The Second Trimester –(14-26weeks)<br>Third Trimester (27 -40)
99	If yes, record the experienced symptoms	1. No symptoms<br>2. Mild Fever<br>3. High grade fever<br>4. Cough<br>5. Cold<br>6. Headache<br>7. Myalgia<br>8. Diarrhea<br>9. Breathlessness<br>10. Loss of test/ smell<br>11. Any other: ________	

No	Question	Response	Instruction
100	Mode of treatment during covid	1. Home isolation<br>2. Hospitalization<br>3. ICU care<br>4. Any other______	
101	If home Isolation, no of days	-------------------------Days	Skip Q:101 if the ans. Of Q:100 is other than home isolation
102	If hospitalized, no of days	-------------------------Days	Skip Q:102 if the ans. Of Q:100 is other than hospitalization
103	If admitted to ICU, no of days	-------------------------Days	Skip Q:103 if the ans. Of Q:100 is other than ICU care
104	Level of SPO2	______________	
105	H/O oxygen support	0. No<br>1. Yes<br>2. NA	
106	If yes, no of days	________	Skip Q:106 if the ans. Of Q:105 is other than yes
107	H/O Steroid during covid treatment	0. No<br>1. Yes	
108	H/O Ventilator during covid treatment	0. No<br>1. Yes	
109	Type of facility for covid treatment	1. Public facility<br>2. Private facility – Physician<br>3. Private facility -BAMS/BHMS<br>4. Trust hospital<br>5. Self-medication<br>6. Any other_______	
110	Latest prescription or pharmacy bill of COVID treatment (Take picture/Choose image)		
111	lab report details	1. Not performed any<br>2. CRP<br>3. D-Dimer<br>4. HB<br>5. ACE-2<br>6. IL-6<br>7. Any Other_________<br>8. Performed but not having any record	Take maximum – min value
112	If performed any, record the details for each with min two values (max, min) and the date		Skip Q:112 if the ans. Of Q:111 is opt. 8 & 1
113	Have you taken any medication after recovering from covid? (Covid follow-up RX and name of the medicine)	0. No<br>1. Yes	
114	If yes, record the detail of the treatment	______	Skip Q:114 if the ans. Of Q:113 is No
115	Have you experienced any pregnancy-related complications during/after covid treatment?	0. No<br>1. Yes	
116	If yes, name the complication.		Skip Q:116-40 if the ans. Of Q:115 is No

No	Question	Response	Instruction
117	If yes, how did you manage this complication?	1. Hospitalized<br>2. Consulted doctor<br>3. Self-medications<br>4. Any other	Skip Q:117-40 if the ans. Of Q:115 is No
118	Remarks	________	

Section 6: Reproductive History (previous) & health care access during pregnancy (consider pandemic pregnancy)

No	Question	Answer	Instruction
119	Status of Gravida	1. Nulliparous<br>2. Multi para	
120	Record the details (in numbers) of	1. Gravida____<br>2. Parity_____<br>3. Abortion ____<br>4. Live births____<br>5. Pre-term birth______	
121	Status of (Covid pandemic pregnancy) on the date of interview	1. Delivered<br>2. Yet to deliver	
122	Date of LMP	__________(dd/mm/year)	
123	Date of EDD	___________(dd/mm/year)	End the tool here if baby is yet to delivered
124	If delivered, outcome of pregnancy	0. Singleton<br>1. Twins<br>2. Triplet<br>3. Any other	
125	How many ANC visit you have completed?	1. Less than 4<br>2. Minimum 4 as per national guideline<br>3. More than 4	
126	Record the reasons if less than 4	_______	
127	Record the reasons if more than 4		
128	What services did you receive at the ANC?	1. BP check<br>2. Hb Check<br>3. Glucose check<br>4. Ultrasound check<br>5. Heart monitor<br>6. Urine analysis<br>7. Lab tasting<br>8. Anthropometry<br>9. Other, pls specify<br>0. Not any	Multiple response is possible
129	What intervention did you receive at your ANC?	1. No Intervention<br>2. Iron -Folic acid tablet<br>3. Calcium tablet<br>4. multi-micronutrient<br>5. De-worming tab<br>6. Counselling for birth planning	Multiple response is possible

No	Question	Answer	Instruction
		7. Others pls specify	
130	Have you experienced any complication during pregnancy?	0. No<br>1. Yes	
131	If yes,	1. Frequent contraction<br>2. Vaginal bleeding<br>3. Vaginal discharge<br>4. Gestational diabetes<br>5. Gestational Hypertension<br>6. sudden hike of Blood pressure<br>7. Convulsion<br>8. Anaemia<br>9. Severe Headache<br>10. Any other<br>11. Not any	
132	Have you experienced any complication during labour?	1. Prolonged labour pain<br>2. Excessive vaginal bleeding<br>3. No cervix dilatation<br>4. Convulsion<br>5. premature rupture of membrane (PROM)<br>6. Hike blood pressure<br>7. Vaginal discharge<br>8. Any other_______<br>9. Not any	
133	Have you experienced any complication after labour (during 42 days of delivery)?	1. Excessive vaginal bleeding<br>2. Fever<br>3. Vaginal discharge<br>4. Convulsion<br>5. Vaginal ulcer<br>6. Not any<br>7. Any other	
134	Actual Date of Delivery	__________(dd/mm/year)	Only for mothers who have delivered
135	Place of delivery	1. Home<br>2. Private institution<br>3. Public institution<br>4. Trust hospital<br>5. Any other	
136	Type of delivery	1. Vaginal<br>2. C-section<br>3. Induced labour	
137	If C-section	1. Medical indication<br>2. Elective	
138	If Medical indication, record the details		
139	In which trimester/term the baby was borne?	1. Full term<br>2. Preterm	
140		0. No	

No	Question	Answer	Instruction
	Were you delivered at the same place (health care facility) where you received the majority of your ANC services?	1. Yes	
141	If, No Record the details (reasons, no of hospital approached and shifted)	____________	
142	Remarks		

Section:6 Maternal Mental Health during the Pandemic

No	Question	Response	Instructions
143	Do you feel the pandemic has affected your mental health?	1. No<br>2. Yes	
144	What impact has the pandemic had on your mental health?	1. Feeling nervous, anxious, or on edge<br>2. Not being able to stop or control worrying/ Worrying too much about different things<br>3. Being so restless that it is hard to sit still<br>4. Becoming easily annoyed or irritable<br>5. Feeling afraid, as if something awful might happen<br>6. Difficulty falling / staying asleep<br>7. Inadequate / not satisfied sleep - less than 7 hours<br>8. Sleeping too much<br>9. Feeling down, depressed, or hopeless<br>10. Feeling tired or having little energy<br>11. Poor appetite or overeating<br>12. Feeling bad about yourself or that you are a failure or have let yourself or your family<br>13. Trouble concentrating on things, such as reading the newspaper or watching television.<br>14. Moving or speaking so slowly that other people could have noticed. Or the opposite being so get or restless that you have been moving around a lot more than usual<br>15. Thoughts that you would be better off dead, or of hurting yourself<br>16. Stressed<br>17. Other	
145	If any other, record the detail	______________________	
146	If feeling nervous, anxious, or on edge - rank it using Scale 1 to 5 (1 is the lowest & 5 is the highest)		
147	If not being able to stop or control worrying - rank it using Scale 1 to 5 (1 is the lowest & 5 is the highest)		
148	If being so restless that it is hard to sit still - rank it using Scale 1 to 5 (1 is the lowest & 5 is the highest)		
149	If becoming easily annoyed or irritable - rank it using Scale 1 to 5 (1 is the lowest & 5 is the highest)		
150	If feeling afraid, as if something awful might happen - rank it using Scale 1 to 5 (1 is the lowest & 5 is the highest)		

No	Question	Response	Instructions
151	If difficulty falling/staying asleep - rank it using Scale 1 to 5 (1 is the lowest & 5 is the highest)		
152	If problems waking up too early / couldn't complete 7 hours sleep - rank it using Scale 1 to 5 (1 is the lowest & 5 is the highest)		
153	If feeling down, depressed, or hopeless- rank it using Scale 1 to 5 (1 is the lowest & 5 is the highest)		
154	If feeling tired or having little energy - rank it using Scale 1 to 5 (1 is the lowest & 5 is the highest)		
155	If poor appetite or overeating - rank it using Scale 1 to 5 (1 is the lowest & 5 is the highest)		
156	If feeling bad about yourself or that you are a failure or have let yourself or your family down- rank it using Scale 1 to 5 (1 is the lowest & 5 is the highest)		
157	If trouble concentrating on things, such as reading the newspaper or watching television - rank it using Scale 1 to 5 (1 is the lowest & 5 is the highest)		
158	If moving or speaking so slowly that other people could have noticed. Or the opposite being so gety or restless that you have been moving around a lot more than usual - rank it using Scale 1 to 5 (1 is the lowest & 5 is the highest)		
159	If thoughts that you would be better o dead, or of hurting yourself- rank it using Scale 1 to 5 (1 is the lowest & 5 is the highest)		
160	If stressed- rank it using Scale 1 to 5 (1 is the lowest & 5 is the highest)		
161	What do you think were the reasons your mental health was impacted during the pandemic?	1. Bereavement<br>2. Isolation<br>3. loss of income<br>4. fear of not having enough food for your household<br>5. Fear of COVID-19 infection<br>fear of losing your unborn baby<br>6. Fear of losing a family member<br>7. Inability to visit ANC/hospital<br>8. Other (write)	
162	If Other, record the details		
163	Do you currently have or had any mental health disorders before the pandemic?	0. No<br>1. Yes	
164	If yes, record the details using prompt from above Q: what impact has the pandemic had on your mental health?	______________	
165	If yes, Record the reason for present mental health condition	______________	
166	Does anyone in your family currently have or had mental health disorder?	0. No<br>1. Yes	

Section:7 Current Food frequency questionnaire

No	Food items	Daily	Four times a week	Thrice a week	Twice a week	Once a week	Once in 15 days	Once in a month	Occasionally /seasonal	Never
**[A]**	**Protein-rich foods**									
1	Bajra									
2	Wheat flour, whole									
3	Bengal gram, dal									
4	Green gram, dal									
5	Lentil, dal									
6	Red gram, dal									
7	Soyabean, white									
8	Groundnut									
9	Egg, poultry, whole, boiled									
10	Chicken, breast									
11	Chicken, liver									
12	Salmon									
13	Tuna									
**[B]**	**Vitamin A and Beta-carotene-rich foods**									
1	Egg, poultry, whole, boiled									
2	Chicken, liver									
3	Goat, liver									
4	Amaranth, leaves									
5	Colocasia, leaves									
6	Drumstick, leaves									
7	Fenugreek leaves									
8	Spinach									
9	Mango, ripe, kesar									
10	Sweet potato, brown skin									
11	Carrot, orange									
**[C]**	**Iron-rich foods**									
1	Bengal gram, whole									
2	Cowpea, brown									
3	Lentil, dal									
4	Moth beans									
5	Peas, dry									
6	Fenugreek, leaves									
7	Drumstick, leaves									
8	Cluster beans									
9	Dates, dry, dark brown									
10	Mint leaves									
11	Gingelly seeds, white									
12	Niger seeds, black									
13	Chicken, liver									
14	Beef, liver									
**[D]**	**Calcium-rich foods**									

No	Food items	Daily	Four times a week	Thrice a week	Twice a week	Once a week	Once in 15 days	Once in a month	Occasionally/seasonal	Never
1	Ragi									
2	Bengal gram, whole									
3	Moth beans									
4	Rajmah, brown									
5	Soyabean, brown									
6	Red gram, whole									
7	Amaranth, leaves, green									
8	Colocasia, leaves									
9	Fenugreek, leaves									
10	Drumstick, leaves									
11	Curry leaves									
12	Mint leaves									
13	Gingelly seeds, white									
14	Paneer									
15	Khoa									

Section:8 Nutrition Access during pandemic

No	Question	Response	Instruction
167	What foods do you usually eat (i.e., usual dietary intake/staple foods)? List and describe		See the current FFQ for the details.
168	On average, how many times in a week do you eat these foods?		
169	Was there a time in the pandemic when you were not able to get these foods?	0. No<br>1. Yes<br>2. Don't know	
170	What foods were you mostly unable to get during the pandemic? List and describe		
171	How frequently were the foods not available during the pandemic?	0. Never<br>1. Often<br>2. sometimes	
172	What was the reason?	0. Poverty/low income (chronic)<br>1. Unemployment<br>2. Systemic racism and racial discrimination<br>3. Unavailable near by/in local market<br>4. Unaffordable (so expensive that people do not have enough money to buy it or pay for it)<br>5. Inaccessible (very difficult/impossible to get)<br>6. Others	Where the participant chooses all or first 3 options, ask what she think was the main reason?
173	What was the main reason?	0. Unavailable<br>1. Unaffordable<br>2. Inaccessible<br>3. Others, specify	Where the participant said the foods were unaffordable (i.e., prices increased), ask what the increment were.

No	Question	Response	Instruction
174	What was the price of these foods before the pandemic?		
175	What was the price of these foods during the pandemic?		
176	Was there a time you had to change your dietary habits because the foods you usually eat were unavailable, inaccessible, or too expensive?	0. No<br>1. Yes<br>2. Don't know	
177	What changes in dietary habits did you make?	1. Reduced the quantity of foods I eat<br>2. I reduced the amount meals I eat<br>3. I substituted my usual foods to other types<br>4. I eat when I can<br>5. other	
178	What foods did you eat when the foods you usually eat were not available?		
179	Were you eating a three-square meal a day (i.e., breakfast, lunch and dinner) during pregnancy?	1. Yes-always<br>2. Yes-sometimes<br>3. No	
180	What meals do you almost always (usually) eat during the pandemic?	1. Breakfast, lunch, dinner<br>2. breakfast & lunch<br>3. breakfast & dinner<br>4. lunch & dinner	
