# Supplementary material for: Impact of SARS-CoV-2 infection and mitigation strategy during pregnancy on prenatal outcome, growth and development in early childhood in India: a UKRI GCRF Action Against Stunting Hub protocol paper
Source: BMJ Paediatr Open. 2024 Feb 27;8(Suppl 1):e001900. doi: 10.1136/bmjpo-2023-001900 (PMC10900341; doi:10.1136/bmjpo-2023-001900)
Supplement: Supplementary data [file bmjpo-2023-001900supp002.pdf]

Longitudinal follow-up of babies born to covid-19 positive antenatal mothers in Gujarat - Children

Basic details	
Unique ID (Children)	☐☐ ☐☐ ☐☐☐ ☐☐
Name of the districts	1. Ahmedabad<br>2. Sabarkatha
Maternal status of covid infection during pregnancy	0. No<br>1. Yes
Date of Interview	
Name of RA	

Section:1 Birth Outcome details

Sr.no	Questions	Response	Instructions
1	Birth date of the child	dd/mm/yyyy	
2	Age of the baby	☐☐ In months	*verbal*
3	Gender	1. Male<br>2. Female	
4	Birth weight	______________Kg	
5	Did the baby cry immediately after birth? (recorded/maternal recall)	0. No<br>1. Yes	
6	Did respiration rate normal immediately after birth? (recorded/maternal recall)	0. No<br>1. Yes	
7	Did the baby's feeding reflexes normal immediately after birth? (recorded/maternal recall)	0. No<br>1. Yes	
8	Did the baby develop COVID-related symptoms (cough, cold, fever, breathlessness) within 24 hours of birth?	0. No<br>1. Yes<br>2. Don't know	
9	If yes, record the detail of whether he/she went for confirmatory investigations & if yes, mention the result	0. No<br>1. Yes	
10	Did the child admitted to NICU/PICU?	0. No<br>1. Yes	

Sr.no	Questions	Response	Instructions
11	If yes, the reason for admission		Skip Q:11 if the answer is no in Q:10.
12	Any congenital abnormality in the baby	0. No<br>1. Yes	
13	If yes, please specify		Skip Q:13 if the answer is no in Q:12.

Section:2 Anthropometry & Morbidity Profile

Sr.no	Question	Response	Instruction
14	Current weight in Kg	_________kg	
15	Current Height in cm	_________cm	
16	Current MUAC in cm	_________cm	
17	Head circumference in Cm	_________cm	
18	Does the child have any infection within the past seven days?	0. No<br>1. Yes	
19	If yes, record the details.		
20	Present status of 1st baby	0. Alive and healthy<br>1. Alive with some health issue<br>2. Dead	
21	Specify the condition if alive with some health issue (1st baby).	______________	Skip Q:21 if answer 20 is other than opt.1
22	Any other major health complications to the baby after birth?	1. No<br>2. Yes	
23	If yes, record the details.	________________	
24	Any other H/O hospitalization to date?	0. No<br>1. Yes	
25	If yes cause of hospitalization	______________	Skip Q:25 if the answer is no in Q:24.

Section:2 IYCF Practice & Immunization details

Sr.no	Question	Response	Instructions
26	Initiation of breastfeeding?	1. within an hour of birth<br>2. within 2-4 hours of birth<br>3. Within 24 hours of birth<br>4. After one day<br>5. Within three days<br>6. any Other_______	
27	Did the baby receive the first yellowish breast milk which came out after birth?		
28	Till what age baby was given only breastmilk?	________months	
29	Age eligibility for immunization on the date of the 1st interview	1. less than six weeks<br>2. 6 weeks<br>3. 10 weeks<br>4. 14 weeks<br>5. 9-12 months<br>6. 16 -24 month	
30	If the child's age is less than six weeks	1. BCG<br>2. OPV-0<br>3. Hepatitis birth dose<br>4. Not any	Multi response
31	If the child's age is up to 6 weeks	1. BCG<br>2. OPV-0<br>3. Hepatitis birth dose<br>4. OPV-1<br>5. PENTAVALENT-1<br>6. FIPV-1<br>7. ROTA-1<br>8. PCV-1<br>9. Not any	Multi response
32	If the child's age is up to 10 weeks	1. BCG<br>2. OPV-0<br>3. Hepatitis birth dose<br>4. OPV-1<br>5. PENTAVALENT-1<br>6. FIPV-1<br>7. ROTA-1<br>8. PCV-1<br>9. OPV-2<br>10. PENTAVALENT-2<br>11. ROTA-2<br>12. Not any	Multi response

Sr.no	Question	Response	Instructions
33	If the child's age is up to 14 weeks	1. BCG<br>2. OPV-0<br>3. Hepatitis birth dose<br>4. OPV-1<br>5. PENTAVALENT-1<br>6. FIPV-1<br>7. ROTA-1<br>8. PCV-1<br>9. OPV-2<br>10. PENTAVALENT-2<br>11. ROTA-2<br>12. OPV-3<br>13. PENTAVALENT-3<br>14. FIPV-2<br>15. ROTA-3<br>16. PCV-2<br>17. Not any	Multi response
34	If the child's age is up to 9 - 12 months	1. BCG<br>2. OPV-0<br>3. Hepatitis birth dose<br>4. OPV-1<br>5. PENTAVALENT-1<br>6. FIPV-1<br>7. ROTA-1<br>8. PCV-1<br>9. OPV-2<br>10. PENTAVALENT-2<br>11. ROTA-2<br>12. OPV-3<br>13. PENTAVALENT-3<br>14. FIPV-2<br>15. ROTA-3<br>16. PCV-2<br>17. MR-1<br>18. PCV-BOOSTER<br>19. Not any	Multi response

Sr.no	Question	Response	Instructions
35	If the child's age is up to 16-24 months	20. BCG<br>21. OPV-0<br>22. Hepatitis birth dose<br>23. OPV-1<br>24. PENTAVALENT-1<br>25. FIPV-1<br>26. ROTA-1<br>27. PCV-1<br>28. OPV-2<br>29. PENTAVALENT-2<br>30. ROTA-2<br>31. OPV-3<br>32. PENTAVALENT-3<br>33. FIPV-2<br>34. ROTA-3<br>35. PCV-2<br>36. MR-1<br>37. PCV-BOOSTER<br>38. MR-2<br>39. 2. OPV-BOOSTER<br>40. Not any	
36	Age eligibility for Vaccination during a follow-up visit (FU)	1. less than nine months<br>2. 9-12 months - Full Immunization<br>3. 13 -24 months - Complete immunization	
37	Status of immunization for less than nine months (FU)	1. Received all eligible vaccines with the alignment of the national immunization schedule<br>2. Received but not as per schedule<br>3. Not received all eligible vaccine<br>4. Record not found	PCV is applicable to children born after 20th October 2021 in Gujarat; PCV was introduced on 20th October 2021
38	If received but not as per schedule, record the reasons. (1 FU)	__________	
39	If not received all eligible vaccines/missing some, record the reasons. (FU)		
40	Status of immunization for 9-12 months (a child is eligible for Full Immunization) (1st FU)	1. Received all eligible vaccines with the alignment of the national immunization schedule<br>2. Received but not as per schedule<br>3. Not received all eligible vaccine<br>4. Record not found	Full Immunization: One dose of BCG, Measles/MR/MMR, three doses of OPV, Pentavalent, Rotavirus and PCV, and two doses of IPV.
41	If received but not as per schedule, record the reasons. (1st FU)	____________	

Sr.no	Question	Response	Instructions
42	If not received all eligible vaccines/missing some, record the reasons. (1st FU)	___________	
43	Status of immunization for 13-24 months & above (Complete immunization) (1st FU)	0. Received all eligible vaccines with the alignment of the national immunization schedule<br>1. Received but not as per schedule<br>2. Not received all eligible vaccine Record not found	Complete Immunization: All vaccines described in Q:40 + Second dose of Measles/ MR/MMR, and one booster dose of OPV and DPT
44	If received but not as per schedule, record the reasons. (1st FU)	___________	
45	Consent for a biochemical marker?	1. For mother only<br>2. For baby only<br>3. For mother and baby both<br>4. Will let us know after discussion with family<br>5. Not for any	

Section: 3 Food Frequency Questionnaire

No	Food items	Daily	Four /week	Thrice/ week	Twice/ week	Once/ week	Once/ 15 days	Once/ month	Occasionally /seasonal	Never
**[A]**	**Protein-rich foods**									
1	Bajra									
2	Wheat flour, whole									
3	Bengal gram, dal									
4	Green gram, dal									
5	Lentil, dal									
6	Red gram, dal									
7	Soyabean, white									
8	Groundnut									
9	Egg, poultry, whole, boiled									
10	Chicken, breast									
11	Chicken, liver									
12	Salmon									
13	Tuna									
**[B]**	**Vitamin A and Beta-carotene-rich foods**									

No	Food items	Daily	Four /week	Thrice/ week	Twice/ week	Once/ week	Once/ 15 days	Once/ month	Occasionally /seasonal	Never
1	Egg, poultry, whole, boiled									
2	Chicken, liver									
3	Goat, liver									
4	Amaranth, leaves									
5	Colocasia, leaves									
6	Drumstick, leaves									
7	Fenugreek leaves									
8	Spinach									
9	Mango, ripe, kesar									
10	Sweet potato, brown skin									
11	Carrot, orange									
**[C]**	**Iron-rich foods**									
1	Bengal gram, whole									
2	Cowpea, brown									
3	Lentil, dal									
4	Moth beans									
5	Peas, dry									
6	Fenugreek, leaves									
7	Drumstick, leaves									
8	Cluster beans									
9	Dates, dry, dark brown									
10	Mint leaves									
11	Gingelly seeds, white									
12	Niger seeds, black									
13	Chicken, liver									
14	Beef, liver									
**[D]**	**Calcium-rich foods**									
1	Ragi									
2	Bengal gram, whole									
3	Moth beans									
4	Rajmah, brown									

No	Food items	Daily	Four /week	Thrice/ week	Twice/ week	Once/ week	Once/ 15 days	Once/ month	Occasionally /seasonal	Never
5	Soyabean, brown									
6	Red gram, whole									
7	Amaranth, leaves, green									
8	Colocasia, leaves									
9	Fenugreek, leaves									
10	Drumstick, leaves									
11	Curry leaves									
12	Mint leaves									
13	Gingelly seeds, white									
14	Paneer									
15	Khoa									

Section: 4 ECD questionnaire

Sr.no	Question	Response	Instructions
1	Birth date	dd/mm/yyyy	
2	Assessment date	dd/mm/yyyy	
3	Age eligibility for ECD	1. INTER-NDA<br>2. OX-NDA<br>3. Not applicable	
4	Status of ECD data collection	0. Not Collected<br>1. Collected	
5	If not collected, record the reason.		
7	Relationship of carer to the child		
8	What is the child's native (first) language?		
9	Does the child speak/understand any language other than his/her nativelanguage?		
10	Is the child exposed to any language other than his/her native languagefor more than 30 minutes each day on most days?		
11	What is the language in which the assessment is being conducted?		

The INTERGROWTH-21st Project Neurodevelopment Assessment - The INTER-NDA

No	Item	Observed Performance				
1	**Builds a tower of 5 cubes** *(trials=3, demonstration=3)*	5 cubes	3-4 cubes	2 cubes	No attempt	Unable to assess
2	**Names 4 colours when asked to do so** *(trials=1, demonstration=0)*	Names 4 colours	Names 3 colours	Names 1 or 2 colors	Does not name anycolor	Unable to assess
3	**Matches 3 cubes of same colours when requested todo so** (trials=1, demonstration=1 of one colour)	Matches 3 colours	Matches 2 colours	Matches 1 colour	Does not match any colour	Unable to assess
4	**Hands the examiner one cube when asked to do so (Examiner says "Please give me one cube" & keeps palm open for 5 seconds after child has handed over1 cube)** *(trials=1, demonstration=0)*	Hands only oneblock within 5 seconds	Hands only one block in more than 5 seconds	Hands two or more blocks	Does not handany block / No attempt	Unable to assess
5	**Puts the spoon in the cup when asked to do so** *(trials=5, demonstration=0)*	Puts the spoon in cup in ≤3 trials	Puts the spoon in cup in 4-5 trials	Takes the spoon or the cup but does not complete action	No attempt	Unable to assess
6	**Matches shapes on board** *(trials=5, demonstration=partial – removal only)*	All shapes in ≤3 trials	All shapes with repeated demonstrat ion i.e. 4-5 trials	One or two shapesin 4-5 trials	No attempt	Unable to assess

7	**Matches shapes on rotated board** *(trials=5, demonstration=partial – removal only)*	All shapes in ≤3 trials	All shapes with repeated demonstrat ion i.e. 4-5 trials	One or two shapesin 4-5 trials	No attempt	Unable to assess

8	**Points correctly when asked "Where is thedoor/entrance to the room?"** *(trials=5, demonstration=0)*	Identifies door correctly in ≤3 trials	Identifies door correctly in 4-5 trials	Attempts, but doesnot identify door	No attempt	Unable to assess
9	**Puts a raisin precisely inside a small opening in abottle** *(trials=1, demonstration=1, test both hands)*	Precise release of raisin into bottle witheach hand	Clumsy release, raisin falls out of bottle with one ormore hand	Attempts but unsuccessful release with one ormore hand	No attempt	Unable to assess
10	**Drinks water from cup/bottle/sippy cup when placedin front of child** *(trials=1, demonstration=0; maternal recall if observationnot possible)*	Drinks water fromcup/sippy cup without spilling	Drinks clumsily & spills	Attempts but unsuccess ful	No attempt	Unable to assess
11	**Looks towards an object located across the roomwhen pointed at by the examiner** (trials=5)	Looks or points atobject in ≤3 trials	Looks or points atobject in 4-5 trials	Looks at the wrongobject, or attempts but cannot identify object	No attempt	Unable to assess
12	**Pretends to drink from a toy cup when placed in frontof him/her** *(trials=2, demonstration=1 if not spontaneous on firstattempt)*	Spontaneously	After 1 demonstrati on	Partial attempt after1 demonstratio	No attempt	Unable to assess

				n		
13	**Able to make a cup of tea with the toy tea set whenrequested by examiner (Examiner says "Can you make a cup of tea?")** *(trials=2, demonstration=1 if not spontaneous on firstattempt)*	Spontaneously, withpouring motion	After 1 demonstrati on	Partial attempt after1 demonstratio n	No attempt	Unable to assess
14	**Feeds doll when requested to (Examiner says "Canyou give the dolly some tea?")** *(trials=2, demonstration=1 if not spontaneous on firstattempt)*	Spontaneously	After 1 demonstrati on	Partial attempt after1 demonstratio n	No attempt	Unable to assess
15	**Imitates straight horizontal scribble** *(trials=5, demonstration=5)*	≤3 trials	4-5 trials; with difficulty	Attempts (hold crayon)	Cannot hold crayon	Unable to assess
16	**Identifies glitter bracelet under correct washcloth** *(trials=5, demonstration=0, test both sides)*	Finds bracelet correctly in ≤2 trailson both sides	Find bracelet correctly in 3 trials or on one side only	Find bracelet correctly in 4-5 trialsor on one side only	Does not find bracelet or noattempt	Unable to assess
17	**Correctly identifies object groups using plurals** *(concurrent observation)*	Uses 5 plurals	Uses 3-4 plurals	Uses 1-2 plurals	Does not useany plurals	Unable to assess
18	**Asks for toilet by gesture or verbally** *(maternal recall)*	Always	Occasionally	Partial (only for bowel movement)	Never	Unable to assess

19	**Runs**<br>*(maternal recall)*	Runs steadily	Attempts	Walks only	Walks with suppor t	Unable to assess
20	**Throws a ball very near**<br>*(trials=1, demonstration=1; test both hands)*	Good release	Unsteady release	Attempts	No attempt	Unable to assess
21	**Kicks ball**<br>*(maternal recall)*	Kicks ball with kneeflexed	Runs after ball & attempts kicking it	Walks and touchesball with foot	No attempt	Unable to assess
22	**Climbs upstairs holding rail, 2 feet/stair or in adultfashion**<br>*(maternal recall)*	Climbs stairs alone steadily	Climbs stairs alone unsteadily	Climbs stairs with help (uses railing, holds adult's hand)	No attempt	Unable to assess
23	**Uses 2-4 syllable babble such as dada, mama but notspecifically to anything or any person**<br>*(concurrent observation)*	Spontaneously	Mimics	1 syllabe babble e.g. ba, ma, da	None	Unable to assess
24	**Use two words together**<br>*(concurrent observation)*	Two words, appropriate use	Two words, inappropriate use	One word, appropriate use	No attempt	Unable to assess
25	**Indicates by gesture to say no**<br>*(concurrent observation or maternal recall)*	Indicates verbally orby definite gesture all the time	Indicates verbally orby definite gesture some of the time	Attempts, but incomplet e indication	No attempt	Unable to assess
26	**Use of a pronoun e.g. me, my, she, he, it, I**<br>*(concurrent observation)*	≥1 pronoun incorrect	≥1 pronoun,	Use of proper names but	No use	Unable to

		context	incorrect use	not pronouns		assess
27	**How many words does the child use during theassessment other than mama/dada** *(concurrent observation)*	≥8 words	6-7 words	4-5 words	≤3 words	Unable to assess
28	**How many sentences of 3 words or more does thechild use during the assessment?** *(concurrent observation)*	≥2	1	≥1 two word utteranc e	None	Unable to assess
29	**In how many instances does the child follow on atopic of conversation providing new information?** *(concurrent observation)*	At least one, using ≥2 words, proving correct information	At least one, uses single words, provides correct information	Uses any number of words, provides incorrect information	Does not follow up on conversati ons	Unable to assess

31	**Positive Affect**	Never or rarely	Some of the time	Most of the time
32	**Exploration**	Never or rarely	Some of the time	Most of the time
33	**Ease of engagement**	Never or rarely	Some of the time	Most of the time
34	**Cooperativeness**	Never or rarely	Some of the time	Most of the time
35	**Adaptability to change**	Never or rarely	Some of the time	Most of the time
36	**Distractibility**	Never or rarely	Some of the time	Most of the time
37	**Negative Affect**	Never or rarely	Some of the time	Most of the time

The Oxford Neurodevelopment Assessment for 10 – 14 months

Item No	Item	Observed Performance				
1	Builds a tower of 3 cubes<br>*(Trials = 5, demonstration = 5)*	3 cubes in ≤3 trials	3 cubes in 4-5 trials	2 cubes in 5 trials	No attempt	Unable to assess
2	Takes 3 cubes out of cup (Examiner says “Look, I am putting the cubes into the cup – one, two and three. Can you take them out?”)<br>*(Trials =3, demonstration = 0 but visibly place cubes inside cup before asking child to take them out counting as you go)*	Takes all 3 cubes out of the cup in ≤3trials	Takes 2 cubes out of cup	Takes 1 cube out of cup	No attempt	Unableto assess
3	Puts 3 cubes back in cup (Examiner says ‘Can you put the cubes back in the cup?”)<br>*(Trials = 3, demonstration = 0)*	Puts all 3 cubes inside cup in ≤3 trials	Puts 2 cubes inside cup in ≤3 trials	Puts 1 cube inside cup in ≤3 trials	No attempt	Unableto assess
4	Hands the examiner one cube when asked to do so (Examiner says ‘Please can you give me 1 cube?”)<br>*(Trials = 1, demonstration = 0)*	Precise release of one cube into examiner’s hand	Clumsy release, cube falls out of examiner’s or child’s hand, childgives examiner more than one cube	Attempts but unsuccessful release with oneor more hand	No attempt	Unableto assess

5	Squeeze toy to make sound<br>(trials = 1, demonstration = 2 squeezes followed by 2 squeezes)	Squeezes toy to make sound	Squeezes toy but not hard enough to make sound	Picks up toy and plays with it but does not squeeze it	No attempt	Unable to assess
6	Drop squeeze toy on floor (Examiner says “Uh oh, where’s ducky gone?”)<br>(trials = 5 following verbal prompts, demonstration = 0)	Child looks towards object or tries to retrieves it spontaneously	Child looks towards object or tries to retrieve it in ≤3 prompts	Child starts looking for fallen toy on repeated prompting (4-5 prompts)	Child does not look for toy even on repeated prompting	Unable to assess
7	Finds squeeze toy under cup (Examiner hides toy under cup and says ‘Where’s ducky gone now?”)<br>(trials = 1, demonstration = 0; verbal prompts = maximum 3)	Child retrieves toy spontaneously by inverting cup	Child pushes cup or points to cup but does not retrieve toy	Child looks at cup but does not touch it	No attempt	Unable to assess
8	Ask child to give squeeze toy to mummy<br>(trials = 5, demonstration = 0)	Child gives toy to mother in in ≤3 trials	Child gives toy to mother with repeated prompting i.e. 4-5 trials	Child does not give toy to mother or gives it and takes it back	No attempt	Unable to assess

9	Identifies spoon out of 5 objects. Examiner says, "Which one is the spoon?"<br>(trials = 5, demonstration = 0)	Child correctly identifies spoon in ≤3 trials	Child identifies spoon with repeated prompting<br>i.e. 4-5 trials	Child identifies an object but it is not the spoon	No attempt	Unable to asses
10	Identifies sock and toothbrush out of 5 objects. Examiner says, "Show me the sock and the toothbrush".<br>(trials = 3, demonstration = 0)	Child correctly identifies sock and toothbrush correctly	Child identifies one object correctly	Child attempts to identify objects but none<br>are correct	No attempt	Unable to asses
11	Pretends to drink from a toy tea cup when placed in front of him/her (Trials = 2, demonstration = 1 if not spontaneous<br>on first attempt)	Spontaneously	After 1 demonstration	Partial attempt after 1 demonstration	No attempt	Unable To assess
12	Lifts empty cup by handle<br>(if not concurrently observed with item above; demonstration=1, trial = 1)	Lifts cup to mouth by handle using one hand with pincer grasp	Lifts cup to mouth using one hand with full hand grasp	Lifts cup to mouth using both hands – full grasp	Touches cup but does not lift it or no attempt	Unable to assess

13	Feeds doll when requested to. Examiner says, “Can you feed dolly some tea?”<br>(Trials = 2, demonstration = 1 if not spontaneous on first attempt)	Spontaneously	After 1 demonstration	Partial attempt after 1 demonstration	No attempt	Unable to assess
14	Matches shapes in puzzle (objects in 1 line)<br>(Trials = 5, demonstration = 0)	All shapes in ≤3 trials	All shapes in 4-5 trials	1 or 2 shapes	No attempt	Unable to asses
15	Matches shapes on rotated puzzle (objects in 1 line)<br>(Trials = 5, demonstration = 0)	All shapes in ≤3 trials	All shapes in 4-5 trials	1 or 2 shapes	No attempt	Unable to asses
16	Unscrews lid of box to retrieve raisins<br>(Trials = 2, demonstration=1 if first attempt unsuccessful)	Unscrews lid in first attempt	Unscrews lid after demonstration	Attempts but unsuccessful	No attempt	Unable to asses

17	Puts a raisin precisely inside a small opening in a box (Trials =1, demonstration = 1, test both hands)	Precise release of raisin into box with each hand	Clumsy release, raisin falls out of bottle with one or more hand	Attempts but unsuccessful release with one or more hand	No attempt	Unable to assess
18	Thumb-finger tip grasp – grasps raisin between thumb and index finger (Observed during item above, trials = 0, demonstration = 0)	Precisely grasps raisin between thumb & index finger on both	Clumsily grasps raisin between thumb & index finger on either	Grasps the raisin with fingers on one or both hands	Tries to grasp raisin with palm or no attempt	Unable to assess
		hands	hand or both hands			
19	Child imitates 4 different consonant-vowel combinations e.g. 'dolly' 'baby' 'lorry' 'happy' 'cookie' (Trials = 4; can ask child to repeat after mother)	Imitates 3-4 consonant-vowel combinations correctly	Imitates 1-2 consonant-vowel combinations correctly	Imitates but not correctly	No attempt	Unable to assess

20	Responds to name by interrupting activity (scribbling)<br>(Trials = 3, demonstration = 0)	Immediately on calling name when engaged in an activity	Only when mother calls child's name when child is involved in an activity	On repeated calling i.e. 2-3 trials	Does not respond to name	Unable to assess
21	Responds to 'no-no' e.g. stops reaching for an object (more crayons) when you say no-no (Trials = 3, demonstration = 0)	Child stops immediately (may or may not continue reaching after a few seconds)	Child stops on repeating no-no 2- 3 times (may or may not continue reaching after a few seconds)	Child does not stop reaching but looks up	Child does not react to no-no	Unable to assess
22	Transfers ball from one hand to another (Trials =1, demonstration = 1)	Freely transfers ball from right to left and left to right hands	Transfers in one direction only	Unable to release ball during 1 or more transfers	No attempt	Unable to assess
23	Walks sideways with support<br>(Concurrent observation or maternal recall)	Walks 3-4 steps with stepping movements	Walks 1-2 steps with stepping movements	Stands only (with/without support)	Stands momentaril y or no attempt	Unable to assess

24	Stands alone for >3 seconds when placed in that position<br>(Concurrent observation/trials = 1)	Stands without support for >3 seconds	Stands without support for <1-3 seconds and then falls/reaches for support	Attempts but cannot stand at all without support	No attempt	Unable to assess
25	Walks alone<br>(Concurrent observation/maternal recall)	Walks 5 steps unsupported with stepping movements	Walks 1-3 steps unsupported with stepping movements	Walks forward supported by an adult	Cannot walk even if supported	Unable to assess
26	Raises self from sitting to standing position<br>(Concurrent observation/maternal recall)	Can stand up himself/herself with support and stay standing for 5 seconds (chair/table/adult's hand)	Can stand up himself/herself with support but cannot stay standing for 5 seconds (chair/table/adu lt's hand)	Tries to stand up with support but not able to stand up completely	Cannot stand up from sitting position at all,– or no attempt	Unable to assess
27	Two 2-4 syllable babble such as dada, mama but not specifically to any person or any thing<br>(Concurrent observation)	Spontaneously	Mimics	1 syllable bable e.g. ma, da, pa	None	Unable to assess

28	Uses one meaningful word (Concurrent observation)	Spontaneously, and in correct context	Spontaneously, but in incorrect context	Mimics on hearing	Does not use words	Unable to assess
29	Uses two meaningful words together (Concurrent observation)	Spontaneously, and in correct context	Spontaneously, but in incorrect context	Mimics on hearing	Does not use words	Unable to assess
30	Combines word and gesture when asked (Do not demonstrate, trials = 3, use different examples each time)	Combines word and gesture completely and appropriately	Combines word and gesture completely but inappropriately	Combines word and gesture incompletely but inappropriately	Does not combine word and gesture	Unable to assess
31	Positive affect		Never or rarely	Some of the time	Most of the time	
32	Exploration		Never or rarely	Some of the time	Most of the time	
33	Ease of engagement		Never or rarely	Some of the time	Most of the time	
34	Cooperativeness		Never or rarely	Some of the time	Most of the time	
35	Adaptability to change		Never or rarely	Some of the time	Most of the time	
36	Distractibility		Never or rarely	Some of the time	Most of the time	
37	Negative Affect		Never or rarely	Some of the time	Most of the time	
