# Supplementary material for: Impact of SARS-CoV-2 infection and mitigation strategy during pregnancy on prenatal outcome, growth and development in early childhood in India: a UKRI GCRF Action Against Stunting Hub protocol paper
Source: BMJ Paediatr Open. 2024 Feb 27;8(Suppl 1):e001900. doi: 10.1136/bmjpo-2023-001900 (PMC10900341; doi:10.1136/bmjpo-2023-001900)
Supplement: Author's manuscript [file bmjpo-2023-001900.draft_revisions.pdf]

BMJ Paediatrics Open

BMJ Paediatrics Open is committed to open peer review. As part of this commitment we make the peer review history of every article we publish publicly available.

When an article is published we post the peer reviewers' comments and the authors' responses online. We also post the versions of the paper that were used during peer review. These are the versions that the peer review comments apply to.

The versions of the paper that follow are the versions that were submitted during the peer review process. They are not the versions of record or the final published versions. They should not be cited or distributed as the published version of this manuscript.

BMJ Paediatrics Open is an open access journal and the full, final, typeset and author-corrected version of record of the manuscript is available on our site with no access controls, subscription charges or pay-per-view fees (http://bmjpaedsopen.bmj.com).

If you have any questions on BMJ Paediatrics Open's open peer review process please email info.bmjpo@bmj.com

BMJ Paediatrics Open

Impact of SARS-CoV-2 infection and mitigation strategy during pregnancy on prenatal outcome, growth and development in early childhood in India: a UKRI GCRF Action Against Stunting Hub protocol paper

Journal:	*BMJ Paediatrics Open*
Manuscript ID	bmjpo-2023-001900
Article Type:	Protocol
Date Submitted by the Author:	09-Feb-2023

Complete List of Authors:	Jobarteh, Modou; London School of Hygiene & Tropical Medicine, Epidemiology & Population Health<br>Saxena, Deepak B.; Indian Institute of Public Health Gandhinagar, Department of Epidemiology<br>Kulkarni, Bharati; National Institute of Nutrition<br>Shah, Komal; Indian Institute of Public Health Gandhinagar, Department of Epidemiology<br>B, Santosh Kumar; National Institute of Nutrition, Clinical Division<br>Shah, Priyanka; Indian Institute of Public Health Gandhinagar, Department of Epidemiology<br>Memon, Farjana; Indian Institute of Public Health Gandhinagar, Epidemiology<br>Chilumula, Monica; National Institute of Nutrition, Maternal and Child Health, Nutrition<br>Palepu, Dharani; National Institute of Nutrition, Clinical Division<br>Selvaraj, Kiruthika; National Institute of Nutrition<br>Dasi, Teena; National Institute of Nutrition<br>Madhari, Radhika; National Institute of Nutrition<br>Calvo-Urbano, Beatriz; University of London, Department of Pathobiology and Population Sciences<br>Dockrell, Julie; University of London Institute of Education Faculty of Children and Learning<br>Antalek, Catherine; University of London Institute of Education Faculty of Children and Health<br>Davies-Kershaw, Hilary; LSHTM, Department of Population Health<br>Ferguson, Elaine; London School of Hygiene and Tropical Medicine Faculty of Epidemiology and Population Health<br>Heffernan, Claire; London International Development Centre, Department of Pathobiology and Population Sciences; London School of Hygiene and Tropical Medicine Faculty of Epidemiology and Population Health

Keywords:	COVID-19, Growth

SCHOLARONE™
Manuscripts

I, the Submitting Author has the right to grant and does grant on behalf of all authors of the Work (as defined in the below author licence), an exclusive licence and/or a non-exclusive licence for contributions from authors who are: i) UK Crown employees; ii) where BMJ has agreed a CC-BY licence shall apply, and/or iii) in accordance with the terms applicable for US Federal Government officers or employees acting as part of their official duties; on a worldwide, perpetual, irrevocable, royalty-free basis to BMJ Publishing Group Ltd ("BMJ") its licensees and where the relevant Journal is co-owned by BMJ to the co-owners of the Journal, to publish the Work in this journal and any other BMJ products and to exploit all rights, as set out in our licence.

The Submitting Author accepts and understands that any supply made under these terms is made by BMJ to the Submitting Author unless you are acting as an employee on behalf of your employer or a postgraduate student of an affiliated institution which is paying any applicable article publishing charge ("APC") for Open Access articles. Where the Submitting Author wishes to make the Work available on an Open Access basis (and intends to pay the relevant APC), the terms of reuse of such Open Access shall be governed by a Creative Commons licence – details of these licences and which Creative Commons licence will apply to this Work are set out in our licence referred to above.

Other than as permitted in any relevant BMJ Author's Self Archiving Policies, I confirm this Work has not been accepted for publication elsewhere, is not being considered for publication elsewhere and does not duplicate material already published. I confirm all authors consent to publication of this Work and authorise the granting of this licence.

Confidential: For Review Only

Impact of SARS-CoV-2 infection and mitigation strategy during pregnancy on prenatal outcome, growth and development in early childhood in India: a UKRI GCRF Action Against Stunting Hub protocol paper

Modou L. Jobarteh[1], Deepak B. Saxena[2], Bharati Kulkarni[3], Komal Shah[2], Santhosh Kumar Banjara[3], Priyanka Shah[2], Farzana Memon[2], Monica Chilumula[3], Dharani Pratyusha[3], Kiruthika Selvaraj[3], Teena Dasi[3], Radhika Madhari[3], Beatriz Calvo[4], Julie Dockrell[5], Catherine Antalek[5], Hilary Davies-Kershaw[6], Elaine Ferguson[6] and Claire Heffernan[1]

[1] UKRI GCRF Action Against Stunting Hub, London School of Hygiene and Tropical Medicine, 20 Bloomsbury Square, WC1A 2NS, London

[2] Department of Epidemiology, Indian Institute of Public Health, Gandhinagar, Gujarat, India

[3] Clinical Division, ICMR-National Institute of Nutrition, Jamai-Osmania PO, Hyderabad, India

[4] Royal Veterinary College, Royal College Street, NW1 0TU, London

[5] UCL Institute of Education, University College London, 25 Woburn Square, WC1H 0AL, London

[6] Department of Population Health, London School of Hygiene and Tropical Medicine, Keppel Street, WC1E 7HT, London

Address all correspondence to:

Dr Modou L. Jobarteh; email: modou.jobarteh@lshtm.ac.uk

Key words: COVID-19, Pandemic, SARS-CoV-2, India, Early Child development

Confidential: For Review Only

ABSTRACT

Introduction

COVID-19 pandemic has offset some of the gains achieved in global health, particularly in relation to maternal, child health and nutrition. As pregnancy is a period of plasticity where insults acting on maternal environment have far-reaching consequences, the pandemic has had a significant impact on prenatal outcomes, intrauterine and postnatal development of infants. This research will investigate both the direct and indirect impact of COVID-19 pandemic during pregnancy on prenatal outcomes, growth and development in early childhood.

Methods and analysis

Community and hospital data in Hyderabad and Gujarat, India will be used to recruit women who were pregnant during the COVID-19 pandemic and contracted SARS-CoV-2 (severe acute respiratory syndrome coronavirus 2) infection. In comparison to women who were pregnant around the same time and did not contract the virus, the study will investigate the impact of the pandemic on access to healthcare, diet, nutrition, mental health, and prenatal outcomes in 712 women (356 per study arm). Children born to the women will be followed prospectively for 18 months period to investigate the impact of the pandemic on nutrition, health, growth, and neurocognition in early childhood.

Ethics and Dissemination

Ethics approval of the study will be sought from the institutional ethics committees of the Indian Institute of Public Health Gandhinagar (IIPHG), Indian Council of Medical Research (ICMR)-National Institute of Nutrition (NIN), and London School of Hygiene and Tropical Medicine (LSHTM). The findings of the study will be disseminated to policy and research communities through engagements, scientific conferences, seminars, and open-access, peer-reviewed publication.

Confidential: For Review Only

SUMMARY BOX

What is already known about topic?

- COVID-19 pandemic has caused unprecedented disruptions to health, food systems, economies, and the livelihoods of millions of people.
- The pandemic has also contributed to the morbidity and mortality of millions of people globally, and caused unusually higher adverse maternal and birth outcomes, including maternal death, stillbirth, and depression.

What this study adds?

- This study will improve understanding of the longterm impact of SARS-CoV-2 infection and public lockdown during pregnancy on child growth and development.
- It will investigate the impact of the pandemic on access to healthcare, food, and nutrition during pregnancy, and maternal mental health.

How this study might affect research, practice or policy

- This study will be support post-pandemic recovery and resilience efforts and help guide the development of evidence-based approaches and strategies to protect women and children.

Confidential: For Review Only

INTRODUCTION

India has an enduring commitment to improve the nutrition and health of its population [1]. Globally, India has one of the highest burden of undernutrition, with 34.7% of children under-5 years of age having stunted growth and 51.4% of women of reproductive age (i.e., 15 to 49 years) being affected by anaemia [2], which are directly attributable to factors such as inadequate dietary intakes of nutritious foods, infectious diseases, poor hand hygiene and sanitation [3]. These figures and other indicators of maternal and child undernutrition and mortality are likely to have increased with the emergence of the coronavirus (COVID-19) pandemic [4]. The exacerbation is likely due to disruptions to food systems, earnings and nutrition programmes affecting access, affordability of foods and dietary intakes [5-7].

India has been severely impacted by the pandemic with more than half a million (> 530,000 as of January 2023) recorded deaths due to COVID-19 [8]. The second wave of the pandemic in India, which started around March 2021, has been the deadliest so far, affecting many sectors of the country including health systems, the economy and food systems [9]. In India and other affected countries, mitigation strategies such as mandatory face covering, public lockdown and curfews were implemented to curb the spread of the virus, minimise severe forms of the disease, hospital admission and death [10]. Whilst these mitigation strategies are necessary to reduce high infection rates, its introduction can have unintended consequences on high-risk groups such as pregnant women and offspring.

Research has revealed that pregnancy outcomes have worsened worldwide during the pandemic, with an increase in maternal deaths, stillbirths, and maternal depression [11,12], and with adverse effect on early childhood neurocognitive development [13]. During the second wave of the pandemic, hospitals across India reported an unusually high spike in maternal mortality and stillbirths [14]. This was attributed to disruptions in MCH (Maternal and Child Health) services, in part due to pregnant women not attending routine antenatal clinics for fear of contracting the virus and hospitals struggled to support pregnant women who contracted the virus [14].

Confidential: For Review Only

This study will investigate the impact of SARS-CoV-2 infection and public lockdown during pregnancy on growth and development in early childhood in Gujarat and Hyderabad, India. Currently, the data on the impact of SAR-CoV-2 infection or public lockdown during pregnancy on childhood growth and development is very limited. As a result, there is high public health interest to establish research cohorts that can provide an understanding of the long-term impacts of COVID-19 pandemic during pregnancy.

The evidence generated from this study will support post-pandemic recovery and resilience efforts and help support the development of evidence-based approaches and strategies to protect women and children.

Confidential: For Review Only

103 METHODS AND ANALYSIS

104

105 **Study design**

106 This cohort study is designed to longitudinally assess the impact of COVID-19 pandemic on growth
107 and development in early childhood. The study will comprise of two groups of dyads (mother-baby
108 pairs) through recruiting: *a*) women who were pregnant during the COVID-19 pandemic in India and
109 had SARS-CoV-2 infection during the pregnancy, and *b*) women who were pregnant around the same
110 time and had no known SARS-CoV-2 infection (i.e., were tested negative) during the pregnancy.
111 Mother-baby pairs in the two groups will be recruited postnatally, between 0-24 months after birth,
112 and prospectively followed for 18 months period. The groups will be matched for age, sex (of the
113 child) and location (community, village, town, or city) to facilitate unbiased comparison. Study
114 specific assessments will be conducted at enrolment into the study (i.e., within one week of an
115 informed consent) and 6, 12 and 18 months later to investigate the impact of SARS-CoV-2 infection
116 and public lockdown during pregnancy on maternal, birth and the development trajectory of children
117 in the two groups. The conceptual framework of the study is elaborated in **Figure 1**.

118

119 **Outcomes**

120 The primary outcome of this study is growth and development in early childhood, which includes
121 anthropometry (i.e., length, weight, MUAC – Mid Upper Arm Circumference, head circumference,
122 skin fold thickness, and associated Z-scores (including LAZ – length of age Z-score and WLZ –
123 weight of length Z-score)) and neurodevelopment (i.e., cognition, motor, and social development)
124 during the 18 months follow up period. Other outcomes of interest are availability, access and
125 affordability of foods, healthcare and maternal mental health during pregnancy, birth/pregnancy
126 outcome, child morbidity, nutritional status, biochemical makers of inflammation, stress, growth
127 hormone, plasma ACE-2, TMPRSS2, etc. (**Table 1)**

128

129 **Recruitment**

Confidential: For Review Only

The study will be conducted in Ahmedabad and Sabarkantha regions of Gujarat, and Hyderabad, Telangana, India. In Gujarat, hospital and community records of women who had COVID-19 infection (confirmed through PCR – polymerase chain reaction – test) during pregnancy will be used to generate the contacts of women to approach for recruitment. In Hyderabad, women recruited in an existing observational study and tested for COVID-19 infection during pregnancy will be identified for inclusion. Women identified for the study will be approached by study staff. At the first point of contact, study staff will use a brief eligibility questionnaire to confirm the identity and eligibility of the woman. The criteria for eligibility are:

- Women aged 18-45 years
- Pregnant during the coronavirus pandemic in India
- Had a coronavirus test during pregnancy
- Singleton pregnancy
- Baby aged between 0-24 months
- Currently living in and agrees to continue living in areas of Gujarat and Hyderabad during the study period

Once eligibility is confirmed, staff will use the participant information sheet to explain the details of the study procedures. Women will be given up to 48 hours to decide on participation. Informed consent will be obtained through fingerprint or signature, and women who cannot read or write (illiterate) will require the presence of an impartial witness during the consenting process, who must countersign the consent form. In addition, a control group comprising of women who tested negative for SARS-CoV-2 during pregnancy will be recruited alongside the cases. Case and controls will be matched for age and sex of the child, and address (location). Dyads (mother-baby) pairs recruited into the study will be assigned a unique study identification number (study ID) which will be used in all future analysis.

Data and sample collection

Confidential: For Review Only

Maternal Questionnaire

At enrolment, a structured questionnaire will be used to understand the impact of COVID-19 pandemic on access to food, nutrition, healthcare, morbidity, and mental health of women during pregnancy. It will include questions on the participant's socio-economic and health status, including marital status, ethnicity, caste, household size, level of education, employment, sources of income, household water sources, energy type, material possessions, and whether they currently have or had been diagnosed with diseases such as diabetes, hypertension, stroke, or cancer.

The dietary habits of the women during the pandemic will be explored. Participants usual dietary intakes of foods (including staple foods) and sources of these foods will be investigated. We will determine whether the availability, accessibility, affordability, and consumption of the foods have changed during the pandemic. Where changes in dietary habits due to the pandemic are reported, prompts will be asked to establish the extent of the changes. For example, if the affordability of basic foods increase, we will ask how much the increment were, and if changes in food consumption are reported, we will find out whether the changes only relate to the quantity of foods/meals consumed or whether the usual foods/diets were substituted for other foods to facilitate satiety and nutrition.

Participants will be asked whether they visited antenatal clinics during pregnancy, and whether access to the clinics and/or services provided by the clinics were disrupted by the pandemic. Questions will be asked about reasons for non-attendance of antenatal clinics. Where antenatal services were provided during the pandemic, we will explore the level of care received and whether it matches the standard antennal care in India. Furthermore, participants will be asked whether they have received counselling on COVID-19 during pregnancy, and whether the counselling has helped them understand the disease, its impact on pregnancy, vaccination, and misinformation.

The impact of the pandemic on components of their mental health such as stress, anxiety, fatigue, depression, and insomnia will be investigated. We will further investigate the risk factors to the mental health of the women during the pandemic; whether it was related to bereavement, isolation, lockdown, worry/fear of getting infected with COVID-19, fear of losing their unborn child, fear of dying, fear of

Confidential: For Review Only

losing a family member, fear of not having enough food, loss of income and inability to attend antenatal clinics. Women can give as many self-reported underlying risk factors as applicable, but we will seek to establish their top three risk factors.

Pregnancy/birth outcome

Ante-natal cards, hospital records and interviews will be used to collect data on pregnancy outcomes such as pre-eclampsia, gestational diabetes, gestational age, preterm birth, congenital deformities, and delivery type (vaginal – natural or artificially induced labour, and caesarean section). In addition, health records of neonatal outcomes such as birth weight, placenta weight, infant length, head circumference, MUAC will be collected.

Breastfeeding practice

At enrolment, a questionnaire will be used to explore whether breastfeeding practices during the pandemic might have differ from standard practice. The women will be asked whether early initiation of breastfeeding (usually within one hour of birth of the baby to benefit from colostrum breastmilk) was conducted soon after the birth of the baby. Mothers will be asked whether the baby is currently breastfeeding, and if not, when was the baby weaned and age at introduction of complementary feeds including drinks and/or water were introduced. This questionnaire will provide understanding of the behaviours around adherence to common breastfeeding practices such as exclusive breastfeeding under 6 months, continued breastfeeding at 1 year, and the stage of introduction of solid, semi solid, and soft food.

Biochemical analyses

Biomarkers of inflammation, growth, nutrition, SARS-CoV-2 receptor proteins and COVID-19 antibody will be investigated in mothers and children. These biomarkers will facilitate understanding of the long-term/residual effect of the pandemic on critical physiology, susceptibility to the virus, and inter/counteraction between the physiological markers (e.g., between nutrients such as vitamin D, zinc, iron, etc., and markers of SARS-CoV-2 entry into host cell/severity such as ACE2, and TMPRSS2).

Confidential: For Review Only

A nurse trained in phlebotomy will collect 5 mL of venous blood samples from the children at enrolment and at 6 months follow-up visit, and 10 mL of blood sample from the mothers at enrolment only. The samples will be used to estimate nutritional status (i.e., Fe, Zn, folate, Cu, vitamin A, B-vitamins, etc.), growth factors (IGF-1 (insulin-like growth factor-1), IGF-2, IGFBP-3 (insulin-like growth factor binding proteins-3)) inflammation markers (CRP, AGP, cytokines (IL1, IL6, IL10, TNF alpha) and markers related to COVID-19 susceptibility (ACE 2, TMPRSS2, tryptophan, serotonin) and COVID-19 antibody.

Anthropometry

Growth of children will be assessed through anthropometric measurements of weight, length, knee-to-heel length, head circumference, MUAC and skin fold thickness (biceps, triceps, and sub-scapular) at enrolment and follow-up visits. All measurements will be conducted using equipment that are regularly calibrated. Measurements will be conducted in triplicate. The measurements will be used to determine z-scores using the WHO Growth Standards for length-for-age (LAZ), weight-for-length (WLZ), triceps-for-age and sub-scapular-for-age, and to classify the children as stunted or not-stunted (LAZ<-2SD) and wasted or not wasted (WLZ<-2SD). The linear growth rate of children will also be determined

In addition, the height, weight, and MUAC of mothers will be measured only at enrolment and BMI (body mass index) calculated. Where possible, secondary data on weights of the women during pregnancy will be recorded.

Neurocognitive development

Age-appropriate measurements will be used to investigate the impact of the pandemic on cognitive, motor, language, emotional, and social development of children. Three methods will be used to measure both the environment in which childhood development is taking place and the development itself.

Confidential: For Review Only

Home Observation for Measurement of the Environment (HOME) [15] will be used to assess the developmental environment at both visits. HOME assesses the emotional support and cognitive stimulation children receive through their home environment, planned events, and family surroundings. HOME contains both observational (by an assessor) and parent/caregiver reported questionnaire items. The observational items of the assessment cover domains such as the quality of parent-child interactions, cleanliness, and order of the home, and distinct features of the dwelling. Self-report items for parents include questions about their child's activities over the past days or weeks, discipline, and parent-child interactions.

Caregiver Reported Early Development Instrument (CREDI) [16] and Oxford Neurodevelopment Assessment (OX-NDA) [17] for infants will be used to assess early childhood neurocognitive development. CREDI measures development in five inter-related domains (motor, language, cognition, social-emotional, and mental health). CREDI will be administered by assessors to parents/caregivers to report milestones and behaviours that are easy for caregivers to understand, observe, and describe. OX-NDA measures several areas of development including cognition, fine and gross motor skills, expressive and receptive language, behaviour (positive, negative, and global), executive function, empathy, problem solving, attention and social-emotional reactivity. OX-NDA assessment consists mainly of observation items which will be completed primarily by an assessor with some questions completed by the parent/caregiver.

Dietary assessment

Multi-pass, quantitative 24-hour dietary recall method will be used to estimate the food and nutrient intake of mothers and children at enrolment and follow-up visits. Staff trained in the multi-pass method will visit the homes of participants to complete the dietary questionnaire. The dietary intake of mothers and children will be assessed separately. A dietary application will be used to enter recalls of all the foods and drinks mothers and children consumed in the preceding 24 hours. Food portion sizes will be estimated using real foods/food models whenever feasible, photo album, cost-equivalent or number

Confidential: For Review Only

(discrete commercial food such as biscuits) or size (small, medium, large). Food intake data will be translated into intakes of energy and nutrients using the Indian food composition table [18]

Health and morbidity questionnaire

Health and morbidity assessment questionnaire will be used to investigate the burden of disease in mothers and children. At enrolment and follow-up visits, mothers will be asked whether she or her child have had any illness over the last preceding months, and if any medication was administered due to the illness. In case of a hospital admission, information on diagnosis and treatments received while at hospital will be collected. In addition, records of childhood vaccination the children have received will be collected.

Analysis plan

Sample size calculations were based on length-for-age z-score (LAZ) at 18 months of childhood and conducted using STATA with the command "power two means -1.5 -2.0, sd1(1.5) sd2(1.5) power (0.8) alpha (0.05)". The power of the study and the significance level were set at 80% and 5%, respectively. Mean LAZ in children born to non-infected women was assumed to be -1.5 and we were interested in assessing a minimum difference of 0.5 in LAZ between children in the infected and uninfected groups. The standard deviation of LAZ in both groups was assumed to be 1.5. After considering an attrition rate of 20% the final sample size was calculated at 712 (i.e., 356 participants per group).

Characteristics of pregnant women at the time of recruitment will be summarised and presented by cohort. The summaries will include medians and interquartile ranges in the case of continuous variables, and the number of individuals per category and their percentages in the case of categorical variables. The WHO R package (anthro) [19] will be used to calculate LAZ and WLZ at enrolment and at 6 months follow-up. The analyses will be carried out in R using a Bayesian framework by means of JAGS [20] or Stan [21]. Predictor variables will be standardised to facilitate model convergence and the selection of prior distributions. Relatively flat prior distributions compatible with the range of expected values will be selected. The parameters' posterior distributions, mean and 95% credible intervals will be computed

Confidential: For Review Only

and compared. Models will be compared with tools such as the Deviance Information Criterion (DIC) and their Widely Applicable Information Criterion (WAIC).

Generalised linear mixed models (GLMM) will be utilised to evaluate the impact of COVID-19 on children's quantitative outcome variables. GLMMs can account for repeated measures per child over time, by modelling children as random effects. COVID-19 infection status during pregnancy will be modelled as an index variable. Adjusted and unadjusted models will be derived. The latter will include sex of child and study site. Furthermore, the effect of other covariates on children's outcome variables will be investigated by means of univariate and multivariate regression modelling. These covariates include household socioeconomic status, maternal level of education, age, height, weight, parity, as well as selected indicators of access to healthcare, food and nutrition, and mental health.

In addition, index variables will be included in the analysis to independently evaluate the impact of SARS-CoV-2 infection and lockdown during pregnancy on outcomes in childhood. The index variables can take four possible values depending on whether the pregnant woman was COVID-19 positive (c+) or negative (c-) and whether she was exposed (m+) or not (m-) to lockdown during pregnancy: c+m+, c+m-, c-m+, c-m-. Comparisons (i.e., contrasts) of the posterior distributions for each of the four cases will enable us to assess the effect associated with each combination of exposures.

Furthermore, generalised mixed models will be used to evaluate the impact of COVID-19 infection on the women's biochemical measurements. These models will be adjusted by gestational age, maternal age, BMI, etc. COVID-19 infectious status during pregnancy will be modelled as an index variable. The impact of COVID-19 on birth/pregnancy outcomes will be assessed by means of logistic regression.

Patient and public partnership strategy

The study proposal was devised by stakeholders in India including regional government, hospital, and public health officials. A range of community engagement meetings will take place before the recruitment of study participants to inform the communities about the study and its potential impact.

Confidential: For Review Only

The meetings will also provide an avenue to address any concerns or questions regarding the study protocol. Study participants and the public will be involved in the dissemination of the study's findings through community discussion or engagement events

Confidential: For Review Only

ETHICS AND DISSEMINATION

Approval for the study was obtained from the ethics committees of the State Health System Resource Centre, Gandhinagar, Gujarat, India (reference no: SHSRC/2021/2185), Indian Council of Medical Research (ICMR), National Institution of Nutrition, Hyderabad, India (reference no: EC/NEW/INST/2021/1206) and the ethics committee of London School of Hygiene and Tropical Medicine (reference no:72848). The results of the study will be disseminated through public engagement meetings, presentation at conferences, seminars, meetings, and publication in peer-reviewed, open access journal. The results will also be deposited on an open access data depository.

Confidential: For Review Only

Table 1: Assessments to be conducted in mothers and children at enrolment and follow-up visits

Timeline	**Mother**	**Child**
Enrolment	*Questionnaire*: Household conditions<br>*Questionnaire*: Health and nutrition during pregnancy[1]<br>*Anthropometry:* Weight, Height, MUAC, and BMI<br>*Health Records*: SARS-CoV-2 test, date, variant, vaccination status[1]<br>*Blood*: Mineral, vitamins, inflammation, IGF, ACE-2, TMPRSS2<br>*Diet:* Usual dietary intake using 2 non-consecutive 24HDR	*Questionnaire*: Breastfeeding practice<br>*Anthropometry*: Weight, Length, MUAC, Head circumference, etc<br>*Questionnaires*: Cognition (HOME, CREDI, OX-NDA)<br>*Blood*: Minerals, vitamins, inflammation, IGF, ACE-2, TMPRSS-2<br>*Diet*: Usual dietary intake using 2 non-consecutive 24HR<br>*Questionnaire*: Health and morbidity<br>*Health records*: Birth outcomes (preterm, SGA, LBW, etc) ‡
6, 12 and 18 months visits	*Questionnaire*: Health and morbidity<br>*Diet:* Usual dietary intake using 2 non-consecutive 24HDR	*Questionnaire*: Breastfeeding practice<br>*Anthropometry*: Weight, Length, MUAC, etc<br>*Questionnaires*: Cognition (HOME, CREDI, OX-NDA)<br>*Blood*: Minerals, vitamins, inflammation, IGF, ACE-2, TMPRSS-2*<br>*Diet*: Usual dietary intake using 2 non-consecutive 24HDR

‡These are mostly retrospective data. The data will be obtained through ante-natal cards, hospital records and verbal interviews with the mothers.

*Blood samples will be collected only at enrolment and 6 months visits

Confidential: For Review Only

REFERENCE:

1. Haddad, L. Lifting the Curse: Overcoming Persistent Undernutrition in India. *IDS Bulletin* **40**, 1-8 (2009).
2. Ssentongo, P.*, et al.* Global, regional and national epidemiology and prevalence of child stunting, wasting and underweight in low- and middle-income countries, 2006–2018. *Scientific Reports* **11**, 5204 (2021).
3. Black, R.E.*, et al.* Maternal and child undernutrition: global and regional exposures and health consequences. *The Lancet* **371**, 243-260 (2008).
4. Osendarp, S.*, et al.* The COVID-19 crisis will exacerbate maternal and child undernutrition and child mortality in low- and middle-income countries. *Nature Food* **2**, 476-484 (2021).
5. Aday, S. & Aday, M.S. Impact of COVID-19 on the food supply chain. *Food Quality and Safety* **4**, 167-180 (2020).
6. Laborde, D., Herforth, A., Headey, D. & de Pee, S. COVID-19 pandemic leads to greater depth of unaffordability of healthy and nutrient-adequate diets in low- and middle-income countries. *Nature Food* **2**, 473-475 (2021).
7. Picchioni, F., Goulao, L.F. & Roberfroid, D. The impact of COVID-19 on diet quality, food security and nutrition in low and middle income countries: A systematic review of the evidence. *Clin Nutr* **41**, 2955-2964 (2022).
8. Worldometer. COVID-19 Coronavirus pandemic. *Available online at: https://www.worldometers.info/coronavirus/ (accessed Januray 2022).*
9. Singh, J., Rahman, S.A., Ehtesham, N.Z., Hira, S. & Hasnain, S.E. SARS-CoV-2 variants of concern are emerging in India. *Nature Medicine* **27**, 1131-1133 (2021).
10. O'Reilly, K.M.*, et al.* Effective transmission across the globe: the role of climate in COVID-19 mitigation strategies. *The Lancet Planetary Health* **4**, e172 (2020).
11. Chmielewska, B.*, et al.* Effects of the COVID-19 pandemic on maternal and perinatal outcomes: a systematic review and meta-analysis. *The Lancet Global Health* **9**, e759-e772 (2021).
12. Stock, S.J.*, et al.* SARS-CoV-2 infection and COVID-19 vaccination rates in pregnant women in Scotland. *Nature Medicine* (2022).
13. Shuffrey, L.C.*, et al.* Association of Birth During the COVID-19 Pandemic With Neurodevelopmental Status at 6 Months in Infants With and Without In Utero Exposure to Maternal SARS-CoV-2 Infection. *JAMA Pediatrics*, e215563-e215563 (2022).
14. Srivastava, K. Covid-19: Why has India had a spike in stillbirths? *BMJ* **374**, n2133 (2021).
15. Bradley, R.H. & Caldwell, B.M. Home observation for measurement of the environment: A revision of the preschool scale. *American Journal of Mental Deficiency* **84**, 235-244 (1979).
16. McCoy, D.C., Waldman, M. & Fink, G. Measuring early childhood development at a global scale: Evidence from the Caregiver-Reported Early Development Instruments. *Early Childhood Research Quarterly* **45**, 58-68 (2018).
17. Fernandes, M.*, et al.* The INTERGROWTH-21st Project Neurodevelopment Package: a novel method for the multi-dimensional assessment of neurodevelopment in pre-school age children. *PLoS One* **9**, e113360 (2014).

Confidential: For Review Only

18. T. Longvah, R.A., K. Bhaskarachary, K. Venkaiah. Indian Food Composition Tables. *Accessible from* *http://www.ifct2017.com/* (2017).

19. Schumacher, D. anthro: Computation of the WHO Child Growth Standards. R package version 1.0.0.9000. *https://github.com/worldhealthorganization/anthro* (Accessed: March 2022).

20. Plummer, M. JAGS: A program for analysis of Bayesian graphical models using Gibbs sampling. in *Proceedings of the 3rd international workshop on distributed statistical computing*, Vol. 124 1-10 (Vienna, Austria., 2003).

21. Team, S.D. Stan Modeling Language Users Guide and Reference Manual, 2.29. *https://mc-stan.org/users/documentation/* (Accessed: March 2022).

Confidential: For Review Only

CONTRIBUTIONS

The author's contribution to writing the manuscript were as follows: MLJ wrote the first draft and was responsible for compiling the content of the manuscript for submission. CH, DBS, and BK developed the concept of the study. MLJ, CH, DBS, BK, KS, PS, JD, CA, and SK contributed to writing the original study protocol which form the basis of this manuscript. MC, KS, TD, DP FM, RM contributed to development of the protocol and editing the manuscript. HDK and EF developed the methods for anthropometry and dietary intake assessment and contributed to writing the manuscript. BC and MLJ developed and wrote the analysis plan. All authors contributed to the review of the manuscript for publication.

FUNDING STATEMENT

The work was supported by UK Research and Innovation (UKRI) under its Global Challenge Research Fund (GCRF). Funding reference: MR/S01313X/1

COMPETING INTEREST STATEMENT

Authors declared no competing or conflict of interest

Confidential: For Review Only

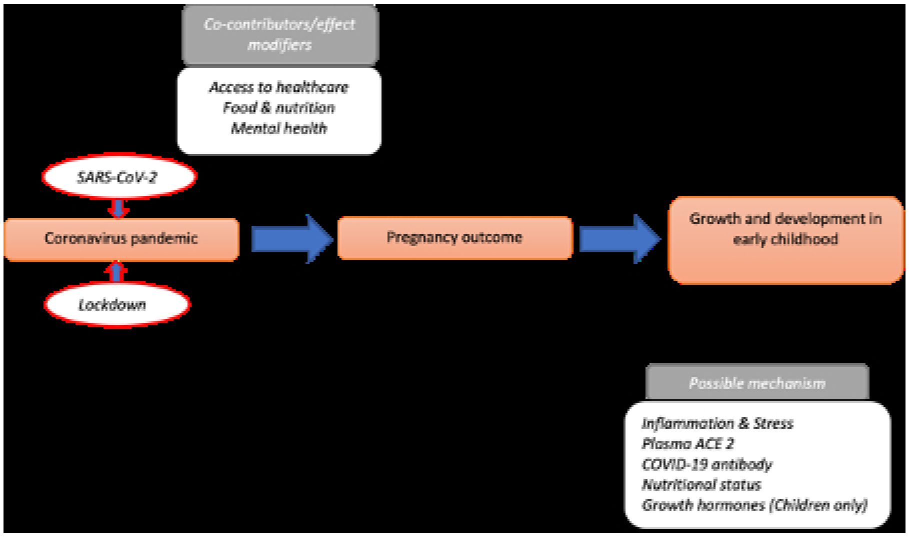

Figure 1: A conceptual framework of the study

The study will investigate the impact of the pandemic (including independently SAR-CoV-2 infection and public lockdown) on pregnancy outcomes and growth and development in early childhood. In addition, the impact of the pandemic on access to healthcare, food and nutrition and mental health in the women and their role, as co-contributor/effect modifiers, in pregnancy and childhood outcomes will be investigated. Furthermore, the study will evaluate possible explanations of the underlying mechanisms through investigation of biomarkers involved in inflammation, stress, plasma ACE2 (angiotensin converting enzyme 2), nutritional status, etc., in mothers and children.

159x93mm (72 x 72 DPI)

BMJ Paediatrics Open

Impact of SARS-CoV-2 infection and mitigation strategy during pregnancy on prenatal outcome, growth and development in early childhood in India: a UKRI GCRF Action Against Stunting Hub protocol paper

Journal:	*BMJ Paediatrics Open*
Manuscript ID	bmjpo-2023-001900.R1
Article Type:	Protocol
Date Submitted by the Author:	14-Apr-2023

Complete List of Authors:	Jobarteh, Modou; London School of Hygiene & Tropical Medicine, Epidemiology & Population Health<br>Saxena, Deepak B.; Indian Institute of Public Health Gandhinagar, Department of Epidemiology<br>Kulkarni, Bharati; National Institute of Nutrition<br>Shah, Komal; Indian Institute of Public Health Gandhinagar, Department of Epidemiology<br>Banjara, Santosh Kumar; National Institute of Nutrition, Clinical Division<br>Shah, Priyanka; Indian Institute of Public Health Gandhinagar, Department of Epidemiology<br>Memon, Farjana; Indian Institute of Public Health Gandhinagar, Epidemiology<br>Chilumula, Monica; National Institute of Nutrition, Maternal and Child Health, Nutrition<br>Palepu, Dharani; National Institute of Nutrition, Clinical Division<br>Selvaraj, Kiruthika; National Institute of Nutrition<br>Dasi, Teena; National Institute of Nutrition<br>Madhari, Radhika; National Institute of Nutrition<br>Calvo-Urbano, Beatriz; University of London, Department of Pathobiology and Population Sciences<br>Dockrell, Julie; University of London Institute of Education Faculty of Children and Learning<br>Antalek, Catherine; University of London Institute of Education Faculty of Children and Health<br>Davies-Kershaw, Hilary; LSHTM, Department of Population Health<br>Ferguson, Elaine; London School of Hygiene and Tropical Medicine Faculty of Epidemiology and Population Health<br>Heffernan, Claire; London International Development Centre, Department of Pathobiology and Population Sciences; London School of Hygiene and Tropical Medicine Faculty of Epidemiology and Population Health

Keywords:	COVID-19, Growth

I, the Submitting Author has the right to grant and does grant on behalf of all authors of the Work (as defined in the below author licence), an exclusive licence and/or a non-exclusive licence for contributions from authors who are: i) UK Crown employees; ii) where BMJ has agreed a CC-BY licence shall apply, and/or iii) in accordance with the terms applicable for US Federal Government officers or employees acting as part of their official duties; on a worldwide, perpetual, irrevocable, royalty-free basis to BMJ Publishing Group Ltd ("BMJ") its licensees and where the relevant Journal is co-owned by BMJ to the co-owners of the Journal, to publish the Work in this journal and any other BMJ products and to exploit all rights, as set out in our licence.

The Submitting Author accepts and understands that any supply made under these terms is made by BMJ to the Submitting Author unless you are acting as an employee on behalf of your employer or a postgraduate student of an affiliated institution which is paying any applicable article publishing charge ("APC") for Open Access articles. Where the Submitting Author wishes to make the Work available on an Open Access basis (and intends to pay the relevant APC), the terms of reuse of such Open Access shall be governed by a Creative Commons licence – details of these licences and which Creative Commons licence will apply to this Work are set out in our licence referred to above.

Other than as permitted in any relevant BMJ Author's Self Archiving Policies, I confirm this Work has not been accepted for publication elsewhere, is not being considered for publication elsewhere and does not duplicate material already published. I confirm all authors consent to publication of this Work and authorise the granting of this licence.

Confidential: For Review Only

Impact of SARS-CoV-2 infection and mitigation strategy during pregnancy on prenatal outcome, growth and development in early childhood in India: a UKRI GCRF Action Against Stunting Hub protocol paper

Modou L. Jobarteh[1], Deepak B. Saxena[2], Bharati Kulkarni[3], Komal Shah[2], Santhosh Kumar Banjara[3], Priyanka Shah[2], Farzana Memon[2], Monica Chilumula[3], Dharani Pratyusha[3], Kiruthika Selvaraj[3], Teena Dasi[3], Radhika Madhari[3], Beatriz Calvo[4], Julie Dockrell[5], Catherine Antalek[5], Hilary Davies-Kershaw[6], Elaine Ferguson[6] and Claire Heffernan[1]

[1] UKRI GCRF Action Against Stunting Hub, London School of Hygiene and Tropical Medicine, 20 Bloomsbury Square, WC1A 2NS, London

[2] Department of Epidemiology, Indian Institute of Public Health, Gandhinagar, Gujarat, India

[3] Clinical Division, ICMR-National Institute of Nutrition, Jamai-Osmania PO, Hyderabad, India

[4] Royal Veterinary College, Royal College Street, NW1 0TU, London

[5] UCL Institute of Education, University College London, 25 Woburn Square, WC1H 0AL, London

[6] Department of Population Health, London School of Hygiene and Tropical Medicine, Keppel Street, WC1E 7HT, London

Address all correspondence to:

Dr Modou L. Jobarteh; email: modou.jobarteh@lshtm.ac.uk

Key words: COVID-19, Pandemic, SARS-CoV-2, India, Early Child development

Confidential: For Review Only

ABSTRACT

Introduction

COVID-19 pandemic has offset some of the gains achieved in global health, particularly in relation to maternal, child health and nutrition. As pregnancy is a period of plasticity where insults acting on maternal environment have far-reaching consequences, the pandemic has had a significant impact on prenatal outcomes, intrauterine and postnatal development of infants. This research will investigate both the direct and indirect impact of COVID-19 pandemic during pregnancy on prenatal outcomes, growth and development in early childhood.

Methods and analysis

Community and hospital data in Hyderabad and Gujarat, India will be used to recruit women who were pregnant during the COVID-19 pandemic and contracted SARS-CoV-2 (severe acute respiratory syndrome coronavirus 2) infection. In comparison to women who were pregnant around the same time and did not contract the virus, the study will investigate the impact of the pandemic on access to healthcare, diet, nutrition, mental health, and prenatal outcomes in 712 women (356 per study arm). Children born to the women will be followed prospectively for 18 months period to investigate the impact of the pandemic on nutrition, health, growth, and neurocognition in early childhood.

Ethics and Dissemination

Ethics approval was granted from the institutional ethics committees of the Indian Institute of Public Health Gandhinagar (IIPHG) (SHSRC/2021/2185), Indian Council of Medical Research (ICMR)-National Institute of Nutrition (NIN) (EC/NEW/INST/2021/1206), and London School of Hygiene and Tropical Medicine (LSHTM) (72848). The findings of the study will be disseminated to policy and research communities through engagements, scientific conferences, seminars, and open-access, peer-reviewed publication.

Confidential: For Review Only

SUMMARY BOX

What is already known about topic?

- COVID-19 pandemic has caused unprecedented disruptions to health, food systems, economies, and the livelihoods of millions of people.
- The pandemic has also contributed to the morbidity and mortality of millions of people globally, and caused unusually higher adverse maternal and birth outcomes, including maternal death, stillbirth, and depression.

What this study adds?

- This study will improve understanding of the longterm impact of SARS-CoV-2 infection and public lockdown during pregnancy on child growth and development.
- It will investigate the impact of the pandemic on access to healthcare, food, and nutrition during pregnancy, and maternal mental health.

How this study might affect research, practice or policy

- This study will be support post-pandemic recovery and resilience efforts and help guide the development of evidence-based approaches and strategies to protect women and children.

Confidential: For Review Only

INTRODUCTION

India has an enduring commitment to improve the nutrition and health of its population [1]. Globally, India has one of the highest burden of undernutrition, with 34.7% of children under-5 years of age having stunted growth and 51.4% of women of reproductive age (i.e., 15 to 49 years) being affected by anaemia [2], which are directly attributable to factors such as inadequate dietary intakes of nutritious foods, infectious diseases, poor hand hygiene and sanitation [3]. These figures and other indicators of maternal and child undernutrition and mortality are likely to have increased with the emergence of the coronavirus (COVID-19) pandemic [4]. The exacerbation is likely due to disruptions to food systems, earnings and nutrition programmes affecting access, affordability of foods and dietary intakes [5-7].

India has been severely impacted by the pandemic with more than half a million (> 530,000 as of January 2023) recorded deaths due to COVID-19 [8]. The second wave of the pandemic in India, which started around March 2021, has been the deadliest so far, affecting many sectors of the country including health systems, the economy and food systems [9]. In India and other affected countries, mitigation strategies such as mandatory face covering, public lockdown and curfews were implemented to curb the spread of the virus, minimise severe forms of the disease, hospital admission and death [10]. Whilst these mitigation strategies are necessary to reduce high infection rates, its introduction can have unintended consequences on high-risk groups such as pregnant women and offspring.

Research has revealed that pregnancy outcomes have worsened worldwide during the pandemic, with an increase in maternal deaths, stillbirths, and maternal depression [11,12], and with adverse effect on early childhood neurocognitive development [13]. During the second wave of the pandemic, hospitals across India reported an unusually high spike in maternal mortality and stillbirths [14]. This was attributed to disruptions in MCH (Maternal and Child Health) services, in part due to pregnant women not attending routine antenatal clinics for fear of contracting the virus and hospitals struggled to support pregnant women who contracted the virus [14].

Confidential: For Review Only

This study will investigate the impact of SARS-CoV-2 infection and public lockdown during pregnancy on growth and development in early childhood in Gujarat and Hyderabad, India. Currently, the data on the impact of SAR-CoV-2 infection or public lockdown during pregnancy on childhood growth and development is very limited. As a result, there is high public health interest to establish research cohorts that can provide an understanding of the long-term impacts of COVID-19 pandemic during pregnancy.

The evidence generated from this study will support post-pandemic recovery and resilience efforts and help support the development of evidence-based approaches and strategies to protect women and children.

Confidential: For Review Only

METHODS AND ANALYSIS

Study design

This cohort study is designed to longitudinally assess the impact of COVID-19 pandemic on growth and development in early childhood. The study will comprise of two groups of dyads (mother-baby pairs) through recruiting: *a*) women who were pregnant during the COVID-19 pandemic in India and had SARS-CoV-2 infection during the pregnancy, and *b*) women who were pregnant around the same time and had no known SARS-CoV-2 infection (i.e., were tested negative) during the pregnancy. Mother-baby pairs in the two groups will be recruited postnatally, between 0-24 months after birth, and prospectively followed for 18 months period. Hospital and community records will be used to match the groups (cases and controls) for age, sex (of the child) and location (community, village, town, or city) to facilitate unbiased comparison. Study specific assessments will be conducted at enrolment into the study (i.e., within one week of an informed consent) and 6, 12 and 18 months later to investigate the impact of SARS-CoV-2 infection and public lockdown during pregnancy on maternal, birth and the development trajectory of children in the two groups. The conceptual framework of the study is elaborated in **Figure 1**.

Outcomes

The primary outcome of this study is growth and development in early childhood, which includes anthropometry (i.e., length, weight, MUAC – Mid Upper Arm Circumference, head circumference, skin fold thickness, and associated Z-scores (including LAZ – length of age Z-score and WLZ – weight of length Z-score)) and neurodevelopment (i.e., cognition, motor, and social development) during the 18 months follow up period. Other outcomes of interest are availability, access and affordability of foods, healthcare and maternal mental health during pregnancy, birth/pregnancy outcome, child morbidity, nutritional status, biochemical makers of inflammation, stress, growth hormone, plasma ACE-2, and TMPRSS2 (**Table 1**).

Recruitment

Confidential: For Review Only

The study will be conducted in Ahmedabad and Sabarkantha regions of Gujarat, and Hyderabad, Telangana, India. In Gujarat, hospital and community records of women who had COVID-19 infection (confirmed through PCR – polymerase chain reaction – test) during pregnancy will be used to generate the contacts of women to approach for recruitment. In Hyderabad, women recruited in an existing observational study and tested for COVID-19 infection during pregnancy will be identified for inclusion. Women identified for the study will be approached by study staff. At the first point of contact, study staff will use a brief eligibility questionnaire to confirm the identity and eligibility of the woman. The criteria for eligibility are:

- Women aged 18-45 years
- Pregnant during the coronavirus pandemic in India
- Had a coronavirus test during pregnancy
- Singleton pregnancy
- Baby aged between 0-24 months
- Currently living in and agrees to continue living in areas of Gujarat and Hyderabad during the study period

Once eligibility is confirmed, staff will use the participant information sheet to explain the details of the study procedures. Women will be given up to 48 hours to decide on participation. Informed consent will be obtained through fingerprint or signature, and women who cannot read or write (illiterate) will require the presence of an impartial witness during the consenting process, who must countersign the consent form. In addition, a control group comprising of women who tested negative for SARS-CoV-2 during pregnancy will be recruited alongside the cases. Case and controls will be matched for age and sex of the child, and address (location). Dyads (mother-baby) pairs recruited into the study will be assigned a unique study identification number (study ID) which will be used in all future analysis.

<u>Data and sample collection</u>

Confidential: For Review Only

Maternal Questionnaire

At enrolment, a structured questionnaire will be used to understand the impact of COVID-19 pandemic on access to food, nutrition, healthcare, morbidity, and mental health of women during pregnancy. It will include questions on the participant's socio-economic and health status, including marital status, ethnicity, caste, household size, level of education, employment, sources of income, household water sources, energy type, material possessions, and whether they currently have or had been diagnosed with diseases such as diabetes, hypertension, stroke, or cancer.

A non-quantitative FFQ (food frequency questionnaire) will be used to explore dietary habits of the women during the pandemic. Participants usual dietary intakes of foods (including staple foods) and sources of these foods will be investigated. We will determine whether the availability, accessibility, affordability, and consumption of the foods have changed during the pandemic. Where changes in dietary habits due to the pandemic are reported, prompts will be asked to establish the extent of the changes. For example, if the affordability of basic foods increase, we will ask how much the increment were, and if changes in food consumption are reported, we will find out whether the changes only relate to the quantity of foods/meals consumed or whether the usual foods/diets were substituted for other foods to facilitate satiety and nutrition.

Participants will be asked whether they visited antenatal clinics during pregnancy, and whether access to the clinics and/or services provided by the clinics were disrupted by the pandemic. Questions will be asked about reasons for non-attendance of antenatal clinics. Where antenatal services were provided during the pandemic, we will explore the level of care received and whether it matches the standard antennal care in India. Furthermore, participants will be asked whether they have received counselling on COVID-19 during pregnancy, and whether the counselling has helped them understand the disease, its impact on pregnancy, vaccination, and misinformation.

The impact of the pandemic on maternal mental health will also be investigated. A qualitative questionnaire will be developed and validated in the population (non-study participants) prior its use.

Confidential: For Review Only

The questionnaire will ask mothers about the impact of the pandemic during pregnancy on components of their mental health including stress, anxiety, fatigue, depression, and insomnia. We will further investigate the risk factors to the mental health of the women during the pandemic; whether it was related to bereavement, isolation, lockdown, worry/fear of getting infected with COVID-19, fear of losing their unborn child, fear of dying, fear of losing a family member, fear of not having enough food, loss of income and inability to attend antenatal clinics. Women can give as many self-reported underlying risk factors as applicable, but we will seek to establish their top three risk factors.

Pregnancy/birth outcome

Ante-natal cards, hospital records and interviews will be used to collect data on pregnancy outcomes such as pre-eclampsia, gestational diabetes, gestational age, preterm birth, congenital deformities, and delivery type (vaginal – natural or artificially induced labour, and caesarean section). In addition, health records of neonatal outcomes such as birth weight, placenta weight, infant length, head circumference, MUAC will be collected.

Breastfeeding practice

At enrolment, a questionnaire will be used to explore whether breastfeeding practices during the pandemic might have differ from standard practice. The women will be asked whether early initiation of breastfeeding (usually within one hour of birth of the baby to benefit from colostrum breastmilk) was conducted soon after the birth of the baby. Mothers will be asked whether the baby is currently breastfeeding, and if not, when was the baby weaned and age at introduction of complementary feeds including drinks and/or water were introduced. This questionnaire will provide understanding of the behaviours around adherence to common breastfeeding practices such as exclusive breastfeeding under 6 months, continued breastfeeding at 1 year, and the stage of introduction of solid, semi solid, and soft food.

Biochemical analyses

Confidential: For Review Only

Biomarkers of inflammation, growth, nutrition, SARS-CoV-2 receptor proteins and COVID-19 antibody will be investigated in mothers and children. These biomarkers will facilitate understanding of the long-term/residual effect of the pandemic on critical physiology, susceptibility to the virus, and inter/counteraction between the physiological markers (including between nutrients, vitamin D, zinc, iron and markers of SARS-CoV-2 entry into host cell/severity such as ACE2, and TMPRSS2).

A nurse trained in phlebotomy will collect 5 mL of venous blood samples from the children at enrolment and at 6 months follow-up visit, and 10 mL of blood sample from the mothers at enrolment only. The samples will be used to estimate nutritional status (Fe, Zn, folate, Cu, vitamin A, and B-vitamins), growth factors (IGF-1 (insulin-like growth factor-1), IGF-2, IGFBP-3 (insulin-like growth factor binding proteins-3)) inflammation markers (CRP, AGP, cytokines (IL1, IL6, IL10, TNF alpha) and markers related to COVID-19 susceptibility (ACE 2, TMPRSS2, tryptophan, serotonin) and COVID-19 antibody.

Anthropometry

Growth of children will be assessed through anthropometric measurements of weight, length, knee-to-heel length, head circumference, MUAC and skin fold thickness (biceps, triceps, and sub-scapular) at enrolment and follow-up visits. All measurements will be conducted using equipment that are regularly calibrated. Measurements will be conducted in triplicate. The measurements will be used to determine z-scores using the WHO Growth Standards for length-for-age (LAZ), weight-for-length (WLZ), triceps-for-age and sub-scapular-for-age, and to classify the children as stunted or not-stunted (LAZ<-2SD) and wasted or not wasted (WLZ<-2SD). The linear growth rate of children will also be determined

In addition, the height, weight, and MUAC of mothers will be measured only at enrolment and BMI (body mass index) calculated. Where possible, secondary data on weights of the women during pregnancy will be recorded.

Neurocognitive development

Confidential: For Review Only

Age-appropriate measurements will be used to investigate the impact of the pandemic on cognitive, motor, language, emotional, and social development of children. Three methods will be used to measure both the environment in which childhood development is taking place and the development itself.

Home Observation for Measurement of the Environment (HOME) [15] will be used to assess the developmental environment at both visits. HOME assesses the emotional support and cognitive stimulation children receive through their home environment, planned events, and family surroundings. HOME contains both observational (by an assessor) and parent/caregiver reported questionnaire items. The observational items of the assessment cover domains such as the quality of parent-child interactions, cleanliness, and order of the home, and distinct features of the dwelling. Self-report items for parents include questions about their child's activities over the past days or weeks, discipline, and parent-child interactions.

Caregiver Reported Early Development Instrument (CREDI) [16] and Oxford Neurodevelopment Assessment (OX-NDA) [17] for infants will be used to assess early childhood neurocognitive development. CREDI measures development in five inter-related domains (motor, language, cognition, social-emotional, and mental health). CREDI will be administered by assessors to parents/caregivers to report milestones and behaviours that are easy for caregivers to understand, observe, and describe. OX-NDA measures several areas of development including cognition, fine and gross motor skills, expressive and receptive language, behaviour (positive, negative, and global), executive function, empathy, problem solving, attention and social-emotional reactivity. OX-NDA assessment consists mainly of observation items which will be completed primarily by an assessor with some questions completed by the parent/caregiver.

Dietary assessment

Multi-pass, quantitative 24-hour dietary recall method will be used to estimate the food and nutrient intake of mothers and children at enrolment and follow-up visits. Staff trained in the multi-pass method

Confidential: For Review Only

will visit the homes of participants to complete the dietary questionnaire. The dietary intake of mothers and children will be assessed separately. A dietary application will be used to enter recalls of all the foods and drinks mothers and children consumed in the preceding 24 hours. Food portion sizes will be estimated using real foods/food models whenever feasible, photo album, cost-equivalent or number (discrete commercial food such as biscuits) or size (small, medium, large). Food intake data will be translated into intakes of energy and nutrients using the Indian food composition table [18]

Health and morbidity questionnaire

Health and morbidity assessment questionnaire will be used to investigate the burden of disease in mothers and children. At enrolment and follow-up visits, mothers will be asked whether she or her child have had any illness over the last preceding months, and if any medication was administered due to the illness. In case of a hospital admission, information on diagnosis and treatments received while at hospital will be collected. In addition, records of childhood vaccination the children have received will be collected.

Analysis plan

Sample size calculations were based on length-for-age z-score (LAZ) at 18 months of childhood and conducted using STATA with the command *"power two means -1.5 -2.0, sd1(1.5) sd2(1.5) power (0.8) alpha (0.05)"*. The power of the study and the significance level were set at 80% and 5%, respectively. Mean LAZ in children born to non-infected women was assumed to be -1.5 and we were interested in assessing a minimum difference of 0.5 in LAZ between children in the infected and uninfected groups. The standard deviation of LAZ in both groups was assumed to be 1.5. After considering an attrition rate of 20% the final sample size was calculated at 712 (i.e., 356 participants per group).

Characteristics of pregnant women at the time of recruitment will be summarised and presented by cohort. The summaries will include medians and interquartile ranges in the case of continuous variables, and the number of individuals per category and their percentages in the case of categorical variables. The WHO R package (anthro) [19] will be used to calculate LAZ and WLZ at enrolment and at 6 months

Confidential: For Review Only

follow-up. The analyses will be carried out in R using a Bayesian framework by means of JAGS [20] or Stan [21]. Predictor variables will be standardised to facilitate model convergence and the selection of prior distributions. The parameters' posterior distributions, mean and 95% credible intervals will be computed and compared. Models will be compared with tools such as the Deviance Information Criterion (DIC) and their Widely Applicable Information Criterion (WAIC).

Generalised linear mixed models (GLMM) will be utilised to evaluate the impact of COVID-19 on children's quantitative outcome variables. GLMMs can account for repeated measures per child over time, by modelling children as random effects. COVID-19 infection status during pregnancy will be modelled as an index variable. Adjusted and unadjusted models will be derived. The latter will include sex of child and study site. Furthermore, the effect of other covariates on children's outcome variables will be investigated by means of univariate and multivariate regression modelling. These covariates include household socioeconomic status, maternal level of education, age, height, weight, parity, as well as selected indicators of access to healthcare, food and nutrition, and mental health.

In addition, index variables will be included in the analysis to independently evaluate the impact of SARS-CoV-2 infection and lockdown during pregnancy on outcomes in childhood. The index variables can take four possible values depending on whether the pregnant woman was COVID-19 positive (c+) or negative (c-) and whether she was exposed (m+) or not (m-) to lockdown during pregnancy: c+m+, c+m-, c-m+, c-m-. Comparisons (i.e., contrasts) of the posterior distributions for each of the four cases will enable us to assess the effect associated with each combination of exposures.

Furthermore, generalised mixed models will be used to evaluate the impact of COVID-19 infection on the women's biochemical measurements. These models will be adjusted by gestational age, maternal age, BMI, etc. COVID-19 infectious status during pregnancy will be modelled as an index variable. The impact of COVID-19 on birth/pregnancy outcomes will be assessed by means of logistic regression.

Patient and public partnership strategy

Confidential: For Review Only

The study proposal was devised by stakeholders in India including regional government, hospital, and public health officials. A range of community engagement meetings will take place before the recruitment of study participants to inform the communities about the study and its potential impact. The meetings will also provide an avenue to address any concerns or questions regarding the study protocol. Study participants and the public will be involved in the dissemination of the study's findings through community discussion or engagement events

Confidential: For Review Only

ETHICS AND DISSEMINATION

Approval for the study was obtained from the ethics committees of the State Health System Resource Centre, Gandhinagar, Gujarat, India (reference no: SHSRC/2021/2185), Indian Council of Medical Research (ICMR), National Institution of Nutrition, Hyderabad, India (reference no: EC/NEW/INST/2021/1206) and the ethics committee of London School of Hygiene and Tropical Medicine (reference no:72848). The results of the study will be disseminated through public engagement meetings, presentation at conferences, seminars, meetings, and publication in peer-reviewed, open access journal. The results will also be deposited on an open access data depository.

Confidential: For Review Only

348

349 **Table 1: Assessments to be conducted in mothers and children at enrolment and follow-up visits**

Timeline	**Mother**	**Child**
Enrolment	*Questionnaire*: Household conditions<br>*Questionnaire*: Health and nutrition during pregnancy[1]<br>*Anthropometry:* Weight, Height, MUAC, and BMI<br>*Health Records*: SARS-CoV-2 test, date, variant, vaccination status[1]<br>*Blood*: Mineral, vitamins, inflammation, IGF, ACE-2, TMPRSS2<br>*Diet:* Usual dietary intake using 2 non-consecutive 24HDR	*Questionnaire*: Breastfeeding practice<br>*Anthropometry*: Weight, Length, MUAC, Head circumference, etc<br>*Questionnaires*: Cognition (HOME, CREDI, OX-NDA)<br>*Blood*: Minerals, vitamins, inflammation, IGF, ACE-2, TMPRSS-2<br>*Diet*: Usual dietary intake using 2 non-consecutive 24HR<br>*Questionnaire*: Health and morbidity<br>*Health records*: Birth outcomes (preterm, SGA, LBW, etc)[1]
6, 12 and 18 months visits	*Questionnaire*: Health and morbidity<br>*Diet:* Usual dietary intake using 2 non-consecutive 24HDR	*Questionnaire*: Breastfeeding practice<br>*Anthropometry*: Weight, Length, MUAC, etc<br>*Questionnaires*: Cognition (HOME, CREDI, OX-NDA)<br>*Blood*: Minerals, vitamins, inflammation, IGF, ACE-2, TMPRSS-2<br>*Diet*: Usual dietary intake using 2 non-consecutive 24HDR

350

351 [1]These are mostly retrospective data. The data will be obtained through ante-natal cards, hospital records and verbal interviews with the mothers.

352 Blood samples will be collected only at enrolment and 6 months visits.

353

354

355

356

357

Confidential: For Review Only

REFERENCE:

1. Haddad, L. Lifting the Curse: Overcoming Persistent Undernutrition in India. *IDS Bulletin* **40**, 1-8 (2009).
2. Ssentongo, P., *et al.* Global, regional and national epidemiology and prevalence of child stunting, wasting and underweight in low- and middle-income countries, 2006–2018. *Scientific Reports* **11**, 5204 (2021).
3. Black, R.E., *et al.* Maternal and child undernutrition: global and regional exposures and health consequences. *The Lancet* **371**, 243-260 (2008).
4. Osendarp, S., *et al.* The COVID-19 crisis will exacerbate maternal and child undernutrition and child mortality in low- and middle-income countries. *Nature Food* **2**, 476-484 (2021).
5. Aday, S. & Aday, M.S. Impact of COVID-19 on the food supply chain. *Food Quality and Safety* **4**, 167-180 (2020).
6. Laborde, D., Herforth, A., Headey, D. & de Pee, S. COVID-19 pandemic leads to greater depth of unaffordability of healthy and nutrient-adequate diets in low- and middle-income countries. *Nature Food* **2**, 473-475 (2021).
7. Picchioni, F., Goulao, L.F. & Roberfroid, D. The impact of COVID-19 on diet quality, food security and nutrition in low and middle income countries: A systematic review of the evidence. *Clin Nutr* **41**, 2955-2964 (2022).
8. Worldometer. COVID-19 Coronavirus pandemic. *Available online at: https://www.worldometers.info/coronavirus/ (accessed Januray 2022).*
9. Singh, J., Rahman, S.A., Ehtesham, N.Z., Hira, S. & Hasnain, S.E. SARS-CoV-2 variants of concern are emerging in India. *Nature Medicine* **27**, 1131-1133 (2021).
10. O'Reilly, K.M., *et al.* Effective transmission across the globe: the role of climate in COVID-19 mitigation strategies. *The Lancet Planetary Health* **4**, e172 (2020).
11. Chmielewska, B., *et al.* Effects of the COVID-19 pandemic on maternal and perinatal outcomes: a systematic review and meta-analysis. *The Lancet Global Health* **9**, e759-e772 (2021).
12. Stock, S.J., *et al.* SARS-CoV-2 infection and COVID-19 vaccination rates in pregnant women in Scotland. *Nature Medicine* (2022).
13. Shuffrey, L.C., *et al.* Association of Birth During the COVID-19 Pandemic With Neurodevelopmental Status at 6 Months in Infants With and Without In Utero Exposure to Maternal SARS-CoV-2 Infection. *JAMA Pediatrics*, e215563-e215563 (2022).
14. Srivastava, K. Covid-19: Why has India had a spike in stillbirths? *BMJ* **374**, n2133 (2021).
15. Bradley, R.H. & Caldwell, B.M. Home observation for measurement of the environment: A revision of the preschool scale. *American Journal of Mental Deficiency* **84**, 235-244 (1979).
16. McCoy, D.C., Waldman, M. & Fink, G. Measuring early childhood development at a global scale: Evidence from the Caregiver-Reported Early Development Instruments. *Early Childhood Research Quarterly* **45**, 58-68 (2018).
17. Fernandes, M., *et al.* The INTERGROWTH-21st Project Neurodevelopment Package: a novel method for the multi-dimensional assessment of neurodevelopment in pre-school age children. *PLoS One* **9**, e113360 (2014).

Confidential: For Review Only

18. T. Longvah, R.A., K. Bhaskarachary, K. Venkaiah. Indian Food Composition Tables. *Accessible from* http://www.ifct2017.com/ (2017).

19. Schumacher, D. anthro: Computation of the WHO Child Growth Standards. R package version 1.0.0.9000. *https://github.com/worldhealthorganization/anthro* (Accessed: March 2022).

20. Plummer, M. JAGS: A program for analysis of Bayesian graphical models using Gibbs sampling. in *Proceedings of the 3rd international workshop on distributed statistical computing*, Vol. 124 1-10 (Vienna, Austria., 2003).

21. Team, S.D. Stan Modeling Language Users Guide and Reference Manual, 2.29. *https://mc-stan.org/users/documentation/* (Accessed: March 2022).

Confidential: For Review Only

CONTRIBUTIONS

The author's contribution to writing the manuscript were as follows: MLJ wrote the first draft and was responsible for compiling the content of the manuscript for submission. CH, DBS, and BK developed the concept of the study. MLJ, CH, DBS, BK, KS, PS, JD, CA, and SK contributed to writing the original study protocol which form the basis of this manuscript. MC, KS, TD, DP FM, RM contributed to development of the protocol and editing the manuscript. HDK and EF developed the methods for anthropometry and dietary intake assessment and contributed to writing the manuscript. BC and MLJ developed and wrote the analysis plan. All authors contributed to the review of the manuscript for publication.

FUNDING STATEMENT

The work was supported by UK Research and Innovation (UKRI) under its Global Challenge Research Fund (GCRF). Funding reference: MR/S01313X/1

COMPETING INTEREST STATEMENT

Authors declared no competing or conflict of interest

Confidential: For Review Only

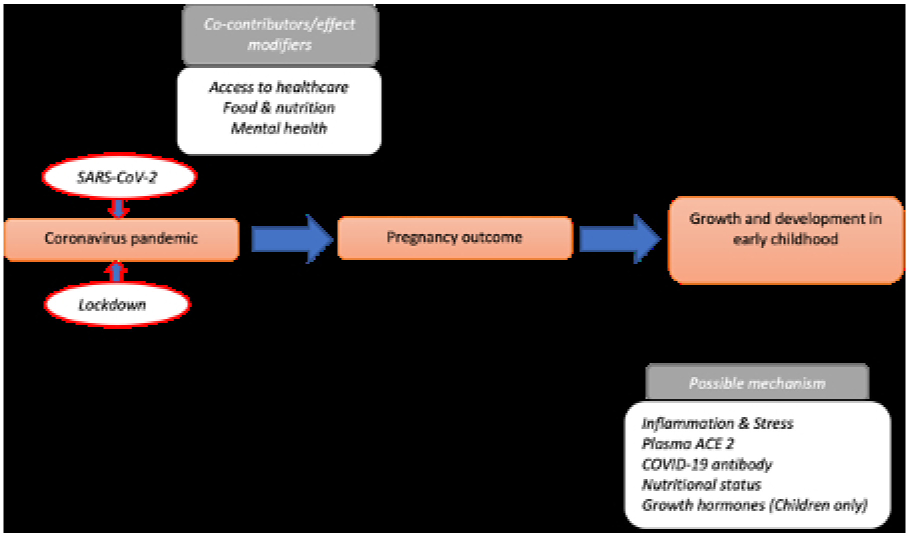

Figure 1: A conceptual framework of the study

The study will investigate the impact of the pandemic (including independently SAR-CoV-2 infection and public lockdown) on pregnancy outcomes and growth and development in early childhood. In addition, the impact of the pandemic on access to healthcare, food and nutrition and mental health in the women and their role, as co-contributor/effect modifiers, in pregnancy and childhood outcomes will be investigated. Furthermore, the study will evaluate possible explanations of the underlying mechanisms through investigation of biomarkers involved in inflammation, stress, plasma ACE2 (angiotensin converting enzyme 2), nutritional status, etc., in mothers and children.

159x93mm (72 x 72 DPI)

BMJ Paediatrics Open

Impact of SARS-CoV-2 infection and mitigation strategy during pregnancy on prenatal outcome, growth and development in early childhood in India: a UKRI GCRF Action Against Stunting Hub protocol paper

Journal:	*BMJ Paediatrics Open*
Manuscript ID	bmjpo-2023-001900.R2
Article Type:	Protocol
Date Submitted by the Author:	07-Jun-2023

Complete List of Authors:	Jobarteh, Modou; London School of Hygiene & Tropical Medicine, Epidemiology & Population Health<br>Saxena, Deepak B.; Indian Institute of Public Health Gandhinagar, Department of Epidemiology<br>Kulkarni, Bharati; National Institute of Nutrition<br>Shah, Komal; Indian Institute of Public Health Gandhinagar, Department of Epidemiology<br>Banjara, Santosh Kumar; National Institute of Nutrition, Clinical Division<br>Shah, Priyanka; Indian Institute of Public Health Gandhinagar, Department of Epidemiology<br>Memon, Farjana; Indian Institute of Public Health Gandhinagar, Epidemiology<br>Chilumula, Monica; National Institute of Nutrition, Maternal and Child Health, Nutrition<br>Palepu, Dharani; National Institute of Nutrition, Clinical Division<br>Selvaraj, Kiruthika; National Institute of Nutrition<br>Dasi, Teena; National Institute of Nutrition<br>Madhari, Radhika; National Institute of Nutrition<br>Calvo-Urbano, Beatriz; University of London, Department of Pathobiology and Population Sciences<br>Dockrell, Julie; University of London Institute of Education Faculty of Children and Learning<br>Antalek, Catherine; University of London Institute of Education Faculty of Children and Health<br>Davies-Kershaw, Hilary; LSHTM, Department of Population Health<br>Ferguson, Elaine; London School of Hygiene and Tropical Medicine Faculty of Epidemiology and Population Health<br>Heffernan, Claire; London International Development Centre, Department of Pathobiology and Population Sciences; London School of Hygiene and Tropical Medicine Faculty of Epidemiology and Population Health

Keywords:	COVID-19, Growth

I, the Submitting Author has the right to grant and does grant on behalf of all authors of the Work (as defined in the below author licence), an exclusive licence and/or a non-exclusive licence for contributions from authors who are: i) UK Crown employees; ii) where BMJ has agreed a CC-BY licence shall apply, and/or iii) in accordance with the terms applicable for US Federal Government officers or employees acting as part of their official duties; on a worldwide, perpetual, irrevocable, royalty-free basis to BMJ Publishing Group Ltd ("BMJ") its licensees and where the relevant Journal is co-owned by BMJ to the co-owners of the Journal, to publish the Work in this journal and any other BMJ products and to exploit all rights, as set out in our licence.

The Submitting Author accepts and understands that any supply made under these terms is made by BMJ to the Submitting Author unless you are acting as an employee on behalf of your employer or a postgraduate student of an affiliated institution which is paying any applicable article publishing charge ("APC") for Open Access articles. Where the Submitting Author wishes to make the Work available on an Open Access basis (and intends to pay the relevant APC), the terms of reuse of such Open Access shall be governed by a Creative Commons licence – details of these licences and which Creative Commons licence will apply to this Work are set out in our licence referred to above.

Other than as permitted in any relevant BMJ Author's Self Archiving Policies, I confirm this Work has not been accepted for publication elsewhere, is not being considered for publication elsewhere and does not duplicate material already published. I confirm all authors consent to publication of this Work and authorise the granting of this licence.

Confidential: For Review Only

Impact of SARS-CoV-2 infection and mitigation strategy during pregnancy on prenatal outcome, growth and development in early childhood in India: a UKRI GCRF Action Against Stunting Hub protocol paper

Modou L. Jobarteh[1], Deepak B. Saxena[2], Bharati Kulkarni[3], Komal Shah[2], Santhosh Kumar Banjara[3], Priyanka Shah[2], Farjana Memon[2], Monica Chilumula[3], Dharani Pratyusha[3], Kiruthika Selvaraj[3], Teena Dasi[3], Radhika Madhari[3], Beatriz Calvo[4], Julie Dockrell[5], Catherine Antalek[5], Hilary Davies-Kershaw[6], Elaine Ferguson[6] and Claire Heffernan[1]

[1] UKRI GCRF Action Against Stunting Hub, London School of Hygiene and Tropical Medicine, 20 Bloomsbury Square, WC1A 2NS, London

[2] Department of Epidemiology, Indian Institute of Public Health, Gandhinagar, Gujarat, India

[3] Clinical Division, ICMR-National Institute of Nutrition, Jamai-Osmania PO, Hyderabad, India

[4] Royal Veterinary College, Royal College Street, NW1 0TU, London

[5] UCL Institute of Education, University College London, 25 Woburn Square, WC1H 0AL, London

[6] Department of Population Health, London School of Hygiene and Tropical Medicine, Keppel Street, WC1E 7HT, London

Address all correspondence to:

Dr Modou L. Jobarteh; email: modou.jobarteh@lshtm.ac.uk

Key words: COVID-19, Pandemic, SARS-CoV-2, India, Early Child development

Confidential: For Review Only

ABSTRACT

Introduction

COVID-19 pandemic has offset some of the gains achieved in global health, particularly in relation to maternal, child health and nutrition. As pregnancy is a period of plasticity where insults acting on maternal environment have far-reaching consequences, the pandemic has had a significant impact on prenatal outcomes, intrauterine and postnatal development of infants. This research will investigate both the direct and indirect impact of COVID-19 pandemic during pregnancy on prenatal outcomes, growth and development in early childhood.

Methods and analysis

Community and hospital data in Hyderabad and Gujarat, India will be used to recruit women who were pregnant during the COVID-19 pandemic and contracted SARS-CoV-2 (severe acute respiratory syndrome coronavirus 2) infection. In comparison to women who were pregnant around the same time and did not contract the virus, the study will investigate the impact of the pandemic on access to healthcare, diet, nutrition, mental health, and prenatal outcomes in 712 women (356 per study arm). Children born to the women will be followed prospectively for 18 months period to investigate the impact of the pandemic on nutrition, health, growth, and neurocognition in early childhood.

Ethics and Dissemination

Ethics approval was granted from the institutional ethics committees of the Indian Institute of Public Health Gandhinagar (IIPHG) (SHSRC/2021/2185), Indian Council of Medical Research (ICMR)-National Institute of Nutrition (NIN) (EC/NEW/INST/2021/1206), and London School of Hygiene and Tropical Medicine (LSHTM) (72848). The findings of the study will be disseminated to policy and research communities through engagements, scientific conferences, seminars, and open-access, peer-reviewed publication.

Confidential: For Review Only

SUMMARY BOX

What is already known about topic?

- COVID-19 pandemic has caused unprecedented disruptions to health, food systems, economies, and the livelihoods of millions of people.
- The pandemic has also contributed to the morbidity and mortality of millions of people globally, and caused unusually higher adverse maternal and birth outcomes, including maternal death, stillbirth, and depression.

What this study adds?

- This study will improve understanding of the longterm impact of SARS-CoV-2 infection and public lockdown during pregnancy on child growth and development.
- It will investigate the impact of the pandemic on access to healthcare, food, and nutrition during pregnancy, and maternal mental health.

How this study might affect research, practice or policy

- This study will be support post-pandemic recovery and resilience efforts and help guide the development of evidence-based approaches and strategies to protect women and children.

Confidential: For Review Only

Confidential: For Review Only

INTRODUCTION

India has an enduring commitment to improve the nutrition and health of its population (1). Globally, India has one of the highest burden of undernutrition, with 34.7% of children under-5 years of age having stunted growth and 51.4% of women of reproductive age (i.e., 15 to 49 years) being affected by anaemia (2), which are directly attributable to factors such as inadequate dietary intakes of nutritious foods, infectious diseases, poor hand hygiene and sanitation (3). These figures and other indicators of maternal and child undernutrition and mortality are likely to have increased with the emergence of the coronavirus (COVID-19) pandemic (4). The exacerbation is likely due to disruptions to food systems, earnings and nutrition programmes affecting access, affordability of foods and dietary intakes (5-7).

India has been severely impacted by the pandemic with more than half a million (> 530,000 as of January 2023) recorded deaths due to COVID-19 (8). The second wave of the pandemic in India, which started around March 2021, has been the deadliest so far, affecting many sectors of the country including health systems, the economy and food systems (9). In India and other affected countries, mitigation strategies such as mandatory face covering, public lockdown and curfews were implemented to curb the spread of the virus, minimise severe forms of the disease, hospital admission and death (10). Whilst these mitigation strategies are necessary to reduce high infection rates, its introduction can have unintended consequences on high-risk groups such as pregnant women and offspring.

Research has revealed that pregnancy outcomes have worsened worldwide during the pandemic, with an increase in maternal deaths, stillbirths, and maternal depression (11, 12), and with adverse effect on early childhood neurocognitive development (13). During the second wave of the pandemic, hospitals across India reported an unusually high spike in maternal mortality and stillbirths (14). This was attributed to disruptions in MCH (Maternal and Child Health) services, in part due to pregnant women not attending routine antenatal clinics for fear of contracting the virus and hospitals struggled to support pregnant women who contracted the virus (14).

This study will investigate the impact of SARS-CoV-2 infection and public lockdown during pregnancy on growth and development in early childhood in Gujarat and Hyderabad, India. Currently, the data on the impact of SAR-CoV-2 infection or public lockdown during pregnancy on childhood growth and development is very limited. As a result, there is high public health interest to establish research cohorts that can provide an understanding of the long-term impacts of COVID-19 pandemic during pregnancy.

The evidence generated from this study will support post-pandemic recovery and resilience efforts and help support the development of evidence-based approaches and strategies to protect women and children.

Confidential: For Review Only

METHODS AND ANALYSIS

Study design

This cohort study is designed to longitudinally assess the impact of COVID-19 pandemic on growth and development in early childhood. The study will comprise of two groups of dyads (mother-baby pairs) through recruiting: *a*) women who were pregnant during the COVID-19 pandemic in India and had SARS-CoV-2 infection during the pregnancy, and *b*) women who were pregnant around the same time and had no known SARS-CoV-2 infection (i.e., were tested negative) during the pregnancy. Mother-baby pairs in the two groups will be recruited postnatally, between 0-24 months after birth, and prospectively followed for 18 months period. Hospital and community records will be used to match the groups (cases and controls) for age, sex (of the child) and location (community, village, town, or city) to facilitate unbiased comparison. Study specific assessments will be conducted at enrolment into the study (i.e., within one week of an informed consent) and 6, 12 and 18 months later to investigate the impact of SARS-CoV-2 infection and public lockdown during pregnancy on maternal, birth and the development trajectory of children in the two groups. The conceptual framework of the study is elaborated in **Figure 1**.

Outcomes

The primary outcome of this study is growth and development in early childhood, which includes anthropometry (i.e., length, weight, MUAC – Mid Upper Arm Circumference, head circumference, skin fold thickness, and associated Z-scores (including LAZ – length of age Z-score and WLZ – weight of length Z-score)) and neurodevelopment (i.e., cognition, motor, and social development) during the 18 months follow up period. Other outcomes of interest are availability, access and affordability of foods, healthcare and maternal mental health during pregnancy, birth/pregnancy outcome, child morbidity, nutritional status, biochemical makers of inflammation, stress, growth hormone, plasma ACE-2, and TMPRSS2 (**Table 1)**.

Recruitment

https://mc.manuscriptcentral.com/bmjpo

The study will be conducted in Ahmedabad and Sabarkantha regions of Gujarat, and Hyderabad, Telangana, India. In Gujarat, hospital and community records of women who had COVID-19 infection (confirmed through PCR – polymerase chain reaction – test) during pregnancy will be used to generate the contacts of women to approach for recruitment. In Hyderabad, women recruited in an existing observational study and tested for COVID-19 infection during pregnancy will be identified for inclusion. Women identified for the study will be approached by study staff. At the first point of contact, study staff will use a brief eligibility questionnaire to confirm the identity and eligibility of the woman. The criteria for eligibility are:

- Women aged 18-45 years
- Pregnant during the coronavirus pandemic in India
- Had a coronavirus test during pregnancy
- Singleton pregnancy
- Baby aged between 0-24 months
- Currently living in and agrees to continue living in areas of Gujarat and Hyderabad during the study period

Once eligibility is confirmed, staff will use the participant information sheet to explain the details of the study procedures. Women will be given up to 48 hours to decide on participation. Informed consent will be obtained through fingerprint or signature, and women who cannot read or write (illiterate) will require the presence of an impartial witness during the consenting process, who must countersign the consent form. In addition, a control group comprising of women who tested negative for SARS-CoV-2 during pregnancy will be recruited alongside the cases. Case and controls will be matched for age and sex of the child, and address (location). Dyads (mother-baby) pairs recruited into the study will be assigned a unique study identification number (study ID) which will be used in all future analysis.

Data and sample collection

Confidential: For Review Only

Maternal Questionnaire

At enrolment, a structured questionnaire will be used to understand the impact of COVID-19 pandemic on access to food, nutrition, healthcare, morbidity, and mental health of women during pregnancy (see online supplemental file). It will include questions on the participant's socio-economic and health status, including marital status, ethnicity, caste, household size, level of education, employment, sources of income, household water sources, energy type, material possessions, and whether they currently have or had been diagnosed with diseases such as diabetes, hypertension, stroke, or cancer.

A non-quantitative FFQ (food frequency questionnaire) will be used to explore dietary habits of the women during the pandemic. Participants usual dietary intakes of foods (including staple foods) and sources of these foods will be investigated. We will determine whether the availability, accessibility, affordability, and consumption of the foods have changed during the pandemic. Where changes in dietary habits due to the pandemic are reported, prompts will be asked to establish the extent of the changes. For example, if the affordability of basic foods increase, we will ask how much the increment were, and if changes in food consumption are reported, we will find out whether the changes only relate to the quantity of foods/meals consumed or whether the usual foods/diets were substituted for other foods to facilitate satiety and nutrition.

Participants will be asked whether they visited antenatal clinics during pregnancy, and whether access to the clinics and/or services provided by the clinics were disrupted by the pandemic. Questions will be asked about reasons for non-attendance of antenatal clinics. Where antenatal services were provided during the pandemic, we will explore the level of care received and whether it matches the standard antennal care in India. Furthermore, participants will be asked whether they have received counselling on COVID-19 during pregnancy, and whether the counselling has helped them understand the disease, its impact on pregnancy, vaccination, and misinformation.

The impact of the pandemic on maternal mental health will also be investigated. A qualitative questionnaire will be developed and validated in the population (non-study participants) prior its use.

Confidential: For Review Only

The questionnaire will ask mothers about the impact of the pandemic during pregnancy on components of their mental health including stress, anxiety, fatigue, depression, and insomnia. We will further investigate the risk factors to the mental health of the women during the pandemic; whether it was related to bereavement, isolation, lockdown, worry/fear of getting infected with COVID-19, fear of losing their unborn child, fear of dying, fear of losing a family member, fear of not having enough food, loss of income and inability to attend antenatal clinics. Women can give as many self-reported underlying risk factors as applicable, but we will seek to establish their top three risk factors.

Pregnancy/birth outcome

Ante-natal cards, hospital records and interviews will be used to collect data on pregnancy outcomes such as pre-eclampsia, gestational diabetes, gestational age, preterm birth, congenital deformities, and delivery type (vaginal – natural or artificially induced labour, and caesarean section). In addition, health records of neonatal outcomes such as birth weight, placenta weight, infant length, head circumference, MUAC will be collected.

Breastfeeding practice

At enrolment, a questionnaire will be used to explore whether breastfeeding practices during the pandemic might have differ from standard practice. The women will be asked whether early initiation of breastfeeding (usually within one hour of birth of the baby to benefit from colostrum breastmilk) was conducted soon after the birth of the baby. Mothers will be asked whether the baby is currently breastfeeding, and if not, when was the baby weaned and age at introduction of complementary feeds including drinks and/or water were introduced. This questionnaire will provide understanding of the behaviours around adherence to common breastfeeding practices such as exclusive breastfeeding under 6 months, continued breastfeeding at 1 year, and the stage of introduction of solid, semi solid, and soft food.

Biochemical analyses

Confidential: For Review Only

Biomarkers of inflammation (CRP, AGP, IL1, IL6, IL10 and TNFα), growth (IGF-1, IGF-2 and IGFBP3), nutrition (Ferritin, Calcium, Vitamin A, Vitamin D and Zinc) SARS-CoV-2 receptor proteins (ACE 2, TMPRSS2) and COVID-19 antibody will be investigated in mothers and children. These biomarkers will facilitate understanding of the long-term/residual effect of the pandemic on critical physiology, susceptibility to the virus, and inter/counteraction between the physiological markers (including between nutrients, vitamin D, zinc, iron and markers of SARS-CoV-2 entry into host cell/severity such as ACE2, and TMPRSS2).

A nurse trained in phlebotomy will collect 5 mL of venous blood samples from the children at enrolment and at 6 months follow-up visit, and 10 mL of blood sample from the mothers at enrolment only. The samples will be used to estimate nutritional status (Fe, Zn, Ca, vitamin A, and D), growth factors (IGF-1 (insulin-like growth factor-1), IGF-2, IGFBP-3 (insulin-like growth factor binding proteins-3)) inflammation markers (CRP, AGP, cytokines (IL1, IL6, IL10, TNF alpha) and markers related to COVID-19 susceptibility (ACE 2, TMPRSS2, tryptophan, serotonin) and COVID-19 antibody.

Anthropometry

Growth of children will be assessed through anthropometric measurements of weight, length, knee-to-heel length, head circumference, MUAC and skin fold thickness (biceps, triceps, and sub-scapular) at enrolment and follow-up visits. All measurements will be conducted using equipment that are regularly calibrated. Measurements will be conducted in triplicate. The measurements will be used to determine z-scores using the WHO Growth Standards for length-for-age (LAZ), weight-for-length (WLZ), triceps-for-age and sub-scapular-for-age, and to classify the children as stunted or not-stunted (LAZ<-2SD) and wasted or not wasted (WLZ<-2SD). The linear growth rate of children will also be determined

In addition, the height, weight, and MUAC of mothers will be measured only at enrolment and BMI (body mass index) calculated. Where possible, secondary data on weights of the women during pregnancy will be recorded.

Confidential: For Review Only

Neurocognitive development

Age-appropriate measurements will be used to investigate the impact of the pandemic on cognitive, motor, language, emotional, and social development of children. Three methods will be used to measure both the environment in which childhood development is taking place and the development itself (see online supplemental file).

Home Observation for Measurement of the Environment (HOME) (15) will be used to assess the developmental environment at both visits. HOME assesses the emotional support and cognitive stimulation children receive through their home environment, planned events, and family surroundings. HOME contains both observational (by an assessor) and parent/caregiver reported questionnaire items. The observational items of the assessment cover domains such as the quality of parent-child interactions, cleanliness, and order of the home, and distinct features of the dwelling. Self-report items for parents include questions about their child's activities over the past days or weeks, discipline, and parent-child interactions.

Caregiver Reported Early Development Instrument (CREDI) (16) and Oxford Neurodevelopment Assessment (OX-NDA) (17) for infants will be used to assess early childhood neurocognitive development. CREDI measures development in five inter-related domains (motor, language, cognition, social-emotional, and mental health). CREDI will be administered by assessors to parents/caregivers to report milestones and behaviours that are easy for caregivers to understand, observe, and describe. OX-NDA measures several areas of development including cognition, fine and gross motor skills, expressive and receptive language, behaviour (positive, negative, and global), executive function, empathy, problem solving, attention and social-emotional reactivity. OX-NDA assessment consists mainly of observation items which will be completed primarily by an assessor with some questions completed by the parent/caregiver.

Dietary assessment

Confidential: For Review Only

Multi-pass, quantitative 24-hour dietary recall method will be used to estimate the food and nutrient intake of mothers and children at enrolment and follow-up visits. Staff trained in the multi-pass method will visit the homes of participants to complete the dietary questionnaire. The dietary intake of mothers and children will be assessed separately. A dietary application will be used to enter recalls of all the foods and drinks mothers and children consumed in the preceding 24 hours. Food portion sizes will be estimated using real foods/food models whenever feasible, photo album, cost-equivalent or number (discrete commercial food such as biscuits) or size (small, medium, large). Food intake data will be translated into intakes of energy and nutrients using the Indian food composition table (18)

Health and morbidity questionnaire

Health and morbidity assessment questionnaire will be used to investigate the burden of disease in mothers and children. At enrolment and follow-up visits, mothers will be asked whether she or her child have had any illness over the last preceding months, and if any medication was administered due to the illness. In case of a hospital admission, information on diagnosis and treatments received while at hospital will be collected. In addition, records of childhood vaccination the children have received will be collected.

Analysis plan

Sample size calculations were based on length-for-age z-score (LAZ) at 18 months of childhood and conducted using STATA with the command *"power two means -1.5 -2.0, sd1(1.5) sd2(1.5) power (0.8) alpha (0.05)"*. The power of the study and the significance level were set at 80% and 5%, respectively. Mean LAZ in children born to non-infected women was assumed to be -1.5 and we were interested in assessing a minimum difference of 0.5 in LAZ between children in the infected and uninfected groups. The standard deviation of LAZ in both groups was assumed to be 1.5. After considering an attrition rate of 20% the final sample size was calculated at 712 (i.e., 356 participants per group).

Characteristics of pregnant women at the time of recruitment will be summarised and presented by cohort. The summaries will include medians and interquartile ranges in the case of continuous variables,

https://mc.manuscriptcentral.com/bmjpo

Confidential: For Review Only

and the number of individuals per category and their percentages in the case of categorical variables. The WHO R package (anthro) (19) will be used to calculate LAZ and WLZ at enrolment and at 6 months follow-up. The analyses will be carried out in R using a Bayesian framework by means of JAGS (20) or Stan (21). Predictor variables will be standardised to facilitate model convergence and the selection of prior distributions. The parameters' posterior distributions, mean and 95% credible intervals will be computed and compared. Models will be compared with tools such as the Deviance Information Criterion (DIC) and their Widely Applicable Information Criterion (WAIC).

Generalised linear mixed models (GLMM) will be utilised to evaluate the impact of COVID-19 on children's quantitative outcome variables. GLMMs can account for repeated measures per child over time, by modelling children as random effects. COVID-19 infection status during pregnancy will be modelled as an index variable. Adjusted and unadjusted models will be derived. The latter will include sex of child and study site. Furthermore, the effect of other covariates on children's outcome variables will be investigated by means of univariate and multivariate regression modelling. These covariates include household socioeconomic status, maternal level of education, age, height, weight, parity, as well as selected indicators of access to healthcare, food and nutrition, and mental health.

In addition, index variables will be included in the analysis to independently evaluate the impact of SARS-CoV-2 infection and lockdown during pregnancy on outcomes in childhood. The index variables can take four possible values depending on whether the pregnant woman was COVID-19 positive (c+) or negative (c-) and whether she was exposed (m+) or not (m-) to lockdown during pregnancy: c+m+, c+m-, c-m+, c-m-. Comparisons (i.e., contrasts) of the posterior distributions for each of the four cases will enable us to assess the effect associated with each combination of exposures.

Furthermore, generalised mixed models will be used to evaluate the impact of COVID-19 infection on the women's biochemical measurements. These models will be adjusted by gestational age, maternal age, BMI, etc. COVID-19 infectious status during pregnancy will be modelled as an index variable. The impact of COVID-19 on birth/pregnancy outcomes will be assessed by means of logistic regression.

Confidential: For Review Only

Patient and public partnership strategy

The study proposal was devised by stakeholders in India including regional government, hospital, and public health officials. A range of community engagement meetings will take place before the recruitment of study participants to inform the communities about the study and its potential impact. The meetings will also provide an avenue to address any concerns or questions regarding the study protocol. Study participants and the public will be involved in the dissemination of the study's findings through community discussion or engagement events

Confidential: For Review Only

ETHICS AND DISSEMINATION

Approval for the study was obtained from the ethics committees of the State Health System Resource Centre, Gandhinagar, Gujarat, India (reference no: SHSRC/2021/2185), Indian Council of Medical Research (ICMR), National Institution of Nutrition, Hyderabad, India (reference no: EC/NEW/INST/2021/1206) and the ethics committee of London School of Hygiene and Tropical Medicine (reference no:72848). The results of the study will be disseminated through public engagement meetings, presentation at conferences, seminars, meetings, and publication in peer-reviewed, open access journal. The results will also be deposited on an open access data depository.

Confidential: For Review Only

Table 1: Assessments to be conducted in mothers and children at enrolment and follow-up visits

Timeline	**Mother**	**Child**
Enrolment	*Questionnaire*: Household conditions<br>*Questionnaire*: Health and nutrition during pregnancy[1]<br>*Anthropometry:* Weight, Height, MUAC, and BMI<br><br>*Health Records*: SARS-CoV-2 test, date, variant, vaccination status[1]<br><br>*Blood*: Fe, Zn, Ca, Vit. A, D, IGF-1, IGF-2, IGFBP3, CRP, AGP, IL1, IL6, IL10, TNFα, ACE-2, TMPRSS2, Serotonin, Tryptophan, SARS-CoV-2 antibody<br><br>*Diet:* Usual dietary intake using 2 non-consecutive 24HDR	*Questionnaire*: Breastfeeding practice<br>*Anthropometry*: Weight, Length, MUAC, Head circumference, etc.<br>*Questionnaires*: Cognition (HOME, CREDI, OX-NDA)<br><br>*Blood*: Fe, Zn, Ca, Vit. A, D, IGF-1, IGF-2, IGFBP3, CRP, AGP, IL1, IL6, IL10, TNFα, ACE-2, TMPRSS-2, Serotonin, Tryptophan, SARS-CoV-2 antibody<br><br>*Diet*: Usual dietary intake using 2 non-consecutive 24HR<br><br>*Questionnaire*: Health and morbidity<br><br>*Health records*: Birth outcomes (preterm, SGA, LBW, etc)[1]
6, 12 and 18 months visits	*Questionnaire*: Health and morbidity<br>*Diet:* Usual dietary intake using 2 non-consecutive 24HDR	*Questionnaire*: Breastfeeding practice<br>*Anthropometry*: Weight, Length, MUAC, Head circumference, etc.<br>*Questionnaires*: Cognition (HOME, CREDI, OX-NDA)<br><br>*Blood*: Fe, Zn, Ca, Vit. A, D, IGF-1, IGF-2, IGFBP3, CRP, AGP, IL1, IL6, IL10, TNFα, ACE-2, TMPRSS-2, Serotonin, Tryptophan, SARS-CoV-2 antibody<br><br>*Diet*: Usual dietary intake using 2 non-consecutive 24HDR

Confidential: For Review Only

[1]These are mostly retrospective data. The data will be obtained through ante-natal cards, hospital records and verbal interviews with the mothers. Blood samples will be collected only at enrolment and 6 months visits.

Confidential: For Review Only

REFERENCE:

1. Haddad, L. Lifting the Curse: Overcoming Persistent Undernutrition in India. *IDS Bulletin* **40**, 1-8 (2009).
2. Ssentongo, P.*, et al.* Global, regional and national epidemiology and prevalence of child stunting, wasting and underweight in low- and middle-income countries, 2006–2018. *Scientific Reports* **11**, 5204 (2021).
3. Black, R.E.*, et al.* Maternal and child undernutrition: global and regional exposures and health consequences. *The Lancet* **371**, 243-260 (2008).
4. Osendarp, S.*, et al.* The COVID-19 crisis will exacerbate maternal and child undernutrition and child mortality in low- and middle-income countries. *Nature Food* **2**, 476-484 (2021).
5. Aday, S. & Aday, M.S. Impact of COVID-19 on the food supply chain. *Food Quality and Safety* **4**, 167-180 (2020).
6. Laborde, D., Herforth, A., Headey, D. & de Pee, S. COVID-19 pandemic leads to greater depth of unaffordability of healthy and nutrient-adequate diets in low- and middle-income countries. *Nature Food* **2**, 473-475 (2021).
7. Picchioni, F., Goulao, L.F. & Roberfroid, D. The impact of COVID-19 on diet quality, food security and nutrition in low and middle income countries: A systematic review of the evidence. *Clin Nutr* **41**, 2955-2964 (2022).
8. Worldometer. COVID-19 Coronavirus pandemic. *Available online at: https://www.worldometers.info/coronavirus/ (accessed Januray 2022).*
9. Singh, J., Rahman, S.A., Ehtesham, N.Z., Hira, S. & Hasnain, S.E. SARS-CoV-2 variants of concern are emerging in India. *Nature Medicine* **27**, 1131-1133 (2021).
10. O'Reilly, K.M.*, et al.* Effective transmission across the globe: the role of climate in COVID-19 mitigation strategies. *The Lancet Planetary Health* **4**, e172 (2020).
11. Chmielewska, B.*, et al.* Effects of the COVID-19 pandemic on maternal and perinatal outcomes: a systematic review and meta-analysis. *The Lancet Global Health* **9**, e759-e772 (2021).
12. Stock, S.J.*, et al.* SARS-CoV-2 infection and COVID-19 vaccination rates in pregnant women in Scotland. *Nature Medicine* (2022).
13. Shuffrey, L.C.*, et al.* Association of Birth During the COVID-19 Pandemic With Neurodevelopmental Status at 6 Months in Infants With and Without In Utero Exposure to Maternal SARS-CoV-2 Infection. *JAMA Pediatrics*, e215563-e215563 (2022).
14. Srivastava, K. Covid-19: Why has India had a spike in stillbirths? *BMJ* **374**, n2133 (2021).
15. Bradley, R.H. & Caldwell, B.M. Home observation for measurement of the environment: A revision of the preschool scale. *American Journal of Mental Deficiency* **84**, 235-244 (1979).
16. McCoy, D.C., Waldman, M. & Fink, G. Measuring early childhood development at a global scale: Evidence from the Caregiver-Reported Early Development Instruments. *Early Childhood Research Quarterly* **45**, 58-68 (2018).
17. Fernandes, M.*, et al.* The INTERGROWTH-21st Project Neurodevelopment Package: a novel method for the multi-dimensional assessment of neurodevelopment in pre-school age children. *PLoS One* **9**, e113360 (2014).

Confidential: For Review Only

18. T. Longvah, R.A., K. Bhaskarachary, K. Venkaiah. Indian Food Composition Tables. *Accessible from* http://www.ifct2017.com/ (2017).
19. Schumacher, D. anthro: Computation of the WHO Child Growth Standards. R package version 1.0.0.9000. https://github.com/worldhealthorganization/anthro (Accessed: March 2022).
20. Plummer, M. JAGS: A program for analysis of Bayesian graphical models using Gibbs sampling. in *Proceedings of the 3rd international workshop on distributed statistical computing*, Vol. 124 1-10 (Vienna, Austria., 2003).
21. Team, S.D. Stan Modeling Language Users Guide and Reference Manual, 2.29. https://mc-stan.org/users/documentation/ (Accessed: March 2022).

Confidential: For Review Only

CONTRIBUTIONS

The author's contribution to writing the manuscript were as follows: MLJ wrote the first draft and was responsible for compiling the content of the manuscript for submission. CH, DBS, and BK developed the concept of the study. MLJ, CH, DBS, BK, KS, PS, JD, CA, and SK contributed to writing the original study protocol which form the basis of this manuscript. MC, KS, TD, DP FM, RM contributed to development of the protocol and editing the manuscript. HDK and EF developed the methods for anthropometry and dietary intake assessment and contributed to writing the manuscript. BC and MLJ developed and wrote the analysis plan. All authors contributed to the review of the manuscript for publication.

FUNDING STATEMENT

The work was supported by UK Research and Innovation (UKRI) under its Global Challenge Research Fund (GCRF). Funding reference: MR/S01313X/1

COMPETING INTEREST STATEMENT

Authors declared no competing or conflict of interest

Confidential: For Review Only

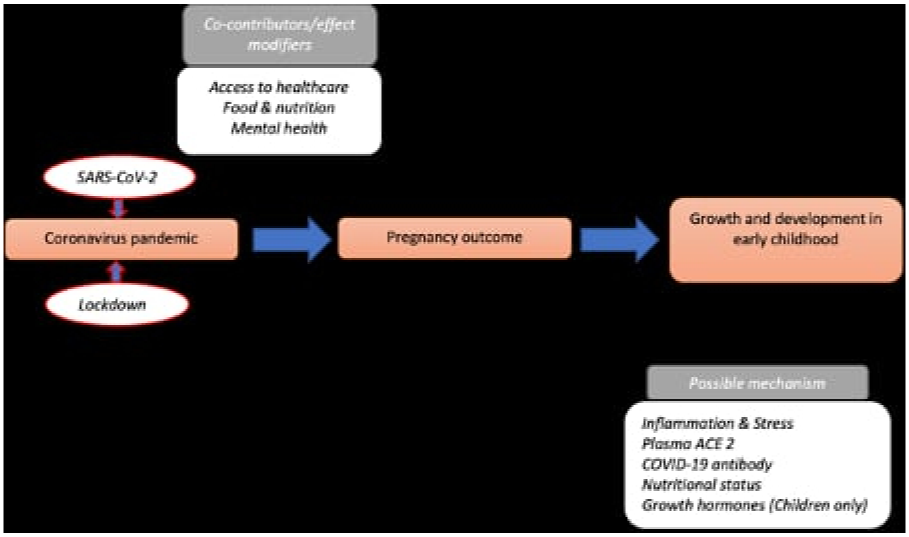

Figure 1: A conceptual framework of the study

The study will investigate the impact of the pandemic (including independently SAR-CoV-2 infection and public lockdown) on pregnancy outcomes and growth and development in early childhood. In addition, the impact of the pandemic on access to healthcare, food and nutrition and mental health in the women and their role, as co-contributor/effect modifiers, in pregnancy and childhood outcomes will be investigated. Furthermore, the study will evaluate possible explanations of the underlying mechanisms through investigation of biomarkers involved in inflammation, stress, plasma ACE2 (angiotensin converting enzyme 2), nutritional status, etc., in mothers and children.

38x22mm (300 x 300 DPI)

Longitudinal follow-up of babies born to covid-19 positive antenatal mothers in Gujarat -Maternal

Basic Details

Question	Responses	Remarks
Name of District	1. Ahmedabad<br>2. Sabarkantha	
Mother UID	☐☐ ☐☐☐ ☐☐	
Status of Covid infection during Pregnancy	0. Negative<br>1. Positive	
Date of Interview		
GPS location		
Name of data collector:		

Section 1: Socio-demographic details

No	Question	Response	Instruction
1	Mother's s Birth date	DOB________	Record verbal age if participants do not remember the birth date
2	Father's s Birth date	DOB________	Record verbal age if participants do not remember the birth date
3	Religion	1. Hindu<br>2. Islam<br>3. Christian<br>4. Others ___________	
4	Caste	1. SC<br>2. ST<br>3. OBC<br>4. General	
5	Education	1. Illiterate<br>2. Can read and write<br>3. Primary<br>4. Secondary<br>5. Higher Secondary<br>6. Graduation<br>7. Postgraduation& above	
6	What type of Ration card do you have?	1. APL (white)<br>2. BPL (yellow)<br>3. Not any<br>4. Not aware/don't know	
7	Occupation	1. Farmer<br>2. Labourer<br>3. Business<br>4. Job<br>5. Housewife<br>6. Any other	
8	What is your family type?	1. Joint<br>2. Nuclear	
9	Total Number of family members in HH (including you)		

Confidential: For Review Only

No	Question	Response	Instruction
10	List all family members with their age and relation to participants in detail.	______________________	
11	Status of current Housing	1. Own<br>2. Rented<br>3. Living with relatives/friends<br>4. Other	
12	Type of house (current)	0. No House/rented<br>1. Hut<br>2. Kuccha House (walls/floors made from mud, roof made from leaves or other low-quality materials)<br>3. Semi-pucca House (Partly low quality and partly high-quality material used)<br>4. Pucca House (High-quality materials used including roof, walls, floor)<br>5. Mansion	
13	Was the status and type of housing being same during covid pandemic?	0. No<br>1. Yes	*If yes, jump to the Q:15*
14	If no, record the difference in terms of both (Type & status)	__________________________	
15	Household fuel source (current)	1. Electricity (Government/state grid)<br>2. Generator (Public/personal)<br>3. Kerosine<br>4. Others (specify)	
16	Was the HH fuel source was same during covid pandemic?	0. No<br>1. Yes	*If yes, jump to the Q:18*
17	If No, record the difference in details.	_______________________________	
18	Cooking fuel source (current)	1. LPG<br>2. Wood/straw<br>3. Animal waste<br>4. Coal/Charcoal<br>5. Kerosine<br>6. Others (specify)	
19	Was the Cooking fuel source being same during the pandemic?	0. No<br>1. Yes	*If yes, jump to the Q:14*
20	If no, record the difference in details.	____________________	
21	Material Possession	1. Livestock (Cow, Sheep, Chicken, etc.)<br>2. Fridge<br>3. TV<br>4. Vehicle (car, motorcycle, etc.)<br>5. Broadband internet<br>6. Others_____<br>7. Not any_____	
22	What is the primary source of water for your Household usage?	1. Piped water into dwelling<br>2. Piped water into the yard/plot<br>3. Piped to neighbour<br>4. Public-tap/standpipe	

Confidential: For Review Only

No	Question	Response	Instruction
		5. Tube-well/borehole<br>6. Dug well<br>7. Water from spring<br>8. Rain water<br>9. Water-vendor -tanker truck/cart delivery<br>10. Open water source (e.g. River, lake, stream, canal)<br>11. Other (specify)	
23	Do you have the same source of drinking water as HH?	0. No<br>1. Yes	*If yes, jump to the Q:25*
24	If not the same, please specify. (Write option number from Q:22)		
25	Do you have to walk/travel to get household/drinking water?	0. No<br>1. Yes	*If NO, jump to the Q:27*
26	If Yes, Distance travelled the closest source of water?		
27	Do you have a toilet facility in your house?	0. No<br>1. Yes	*If NO, jump to the Q:29*
28	If yes, what type of toilet	1. Toilet with water flush or pour-flush<br>2. Ventilated improved pit (VIP) latrine<br>3. Pit latrine with slab<br>4. Pit latrine without slab/open pit<br>5. Twin pit/composting toilet	
29	Since how long are you staying in this house with these amenities?	No of months________	
30	Were these house amenities being same (water source + Toilet facilities _ material passion) during the pandemic?	0. No<br>1. Yes	*If yes, end the Section here*
31	If no, record the details for each of the three parameters	____________________________	

Section 2: Household Income details

No	Question	Response	Instruction
32	Are you involved in any paid work?	0. No<br>1. Yes	*If no jump to the Q:38*
33	if yes, state the type of work.	_________	
34	Do you commute to work?	0. No<br>1. Yes	*If no, jump to the Q:36.*
35	If yes, state the travel distance and the transportation type used.	_______________	
36	How much are you paid per month? State the amount, if possible. If not, please give us a close estimation.		
37	Since how many years have you been involved with this work?		
38	Is your husband involved in any paid work?	0. No<br>1. Yes	*If no, jump to the Q:44.*
39	If yes, state the type of work.	__________	
40	Does he commute to work?	0. No<br>1. Yes	*If no, jump to the Q:42.*

Confidential: For Review Only

No	Question	Response	Instruction
41	If yes, state the distance of travel and the type of transportation used.		
42	How much does he earn per month? State the amount if possible. If not, please give an estimation.		Please use 9 if the information is not known or refused.
43	Since how many years has, he been involved in this same job with the same travelling options?		
44	Does anyone else work in your household?	0. No<br>1. Yes	*If No, end sect. 2 here*
45	If yes, state your relationship (daughter, son, cousin, uncle, etc.) and the type of work.	1. Type of work________<br>2. Relationship with you_____	
46	Do they commute to work?	0. No<br>1. Yes	
47	If yes, state distance of travel and type of transportation.		
48	How much do they earn? State the amount if possible. If not please estimate.		Please use 9 in the data entry if the information is not known/refused.
49	How much have your total household income each month? State amount if known.		If not please estimate. Please use 9 in the data entry if the information is refused.
50	Do you have other sources of income (such as gifts, remittance, inheritance, etc.)	0. No<br>1. Yes	
51	Does the covid-19 pandemic affect your household income?	0. No<br>1. Yes	
52	Whose income was affected in the household?	1. Self<br>2. Husband<br>3. Son<br>4. Daughter<br>5. Uncle<br>6. Any other	
53	How was the income affected?	1. Lost job totally<br>2. lost some earnings<br>3. Other	
54	On average, how much earnings would you say your household has lost during the pandemic? State amount. If not, please estimate.		

Section 3: Current anthropometry, morbidity and addiction details

No	Question	Response	Instruction
55	Are you currently pregnant?	0. No<br>1. Yes	
56	Are you currently breastfeeding?	0. No<br>1. Yes	
57	Are you suffering from any of the listed diseases? (current)	1. Not any<br>2. Diabetes<br>3. Hypertension	Multiple answers possible

Confidential: For Review Only

No	Question	Response	Instruction
		4. Cardiac disease<br>5. Thyroid<br>6. Chronic respiratory diseases<br>7. Immune-compromised status<br>8. Any other_______	
58	Were you suffering from any of the listed diseases during your pregnancy (please consider the pregnancy during the pandemic)?	1. Not any<br>2. Diabetes<br>3. Hypertension<br>4. Cardiac disease<br>5. Thyroid<br>6. Chronic respiratory diseases<br>7. Immune-compromised status<br>8. Any other___	Multiple answers possible – please consider the during the pandemic pregnancy
59	Do you have any infection/illness within the past seven days?	0. No<br>1. Yes	
60	If yes, record the details.	_________________	
61	Do you currently have any addiction to any of the listed items?	1. Not any<br>2. Cigarette/bidi smoking<br>3. Tobacco chewing<br>4. Alcohol<br>5. Any other	
62	If any other, record the details about the item's name with frequency/day.	__________	
63	If the response is except "not any", then record the frequency/day for each type of received response.	_________	
64	Did you have any addiction during pregnancy from the listed items?	1. Not any<br>2. Cigarette/bidi smoking<br>3. Tobacco chewing<br>4. Alcohol<br>5. Any other	
65	If any other, record the details about the item's name with frequency/day.		
66	If the response is except "not any", then record the frequency/day for each type of received response.		
67	Any other history of hospitalization (mother) till date?	1. No<br>2. Yes	
68	If yes, record the reason & no of days		

Confidential: For Review Only

No	Question	Response	Instruction
69	Weight in kg during pregnancy (Recorded)	______________kg	
70	Level of Hb during pregnancy (Recorded)		
71	Level of RBS during pregnancy (Recorded)		
72	BP during pregnancy (Recorded)		
73	Maternal current weight in kg		
74	Weight on the date of the interview		Current anthropometry will be taken on every follow-up visits
75	Height in cm	______________cm	
76	Maternal MUAC on the date of the interview	______________cm	
77	Skin fold thickness – Triceps on the date of the interview	______________mm	
78	Skin fold thickness - Biceps on the date of the interview	______________mm	
79	Skin fold thickness – Scapula on the date of the interview	______________mm	
80	Skin fold thickness – Supra iliac on the date of the interview	______________mm	
81	Waist circumference (in cm) on the date of the interview	______________cm	
82	Hip Circumference (in cm) on the date of the interview	______________cm	

Section 4: Covid Infection Prevention and vaccination details

No	Question	Response	Instruction
83	Have you followed any of the following covid-infection preventive measures strictly and regularly?	1. Face mask wearing<br>2. Handwashing<br>3. Use of hand sanitizer frequently<br>4. Social distancing (Avoiding all general social gatherings)<br>5. Staying away from infected or suspected patients<br>6. Not any<br>7. Any other	*Multi response.*<br>*If Ans: is not any, then jump to 85*
84	If yes, specify the duration	1. During all three waves of pandemic<br>2. During Covid-infection only<br>3. Only if anyone infected in family/close contact<br>4. Not Followed	
85	Have you taken any of the following Prophylactic drugs and dietary supplements to prevent from Covid pandemic	1. Vitamin C<br>2. Vitamin D<br>3. Zink<br>4. Ayurvedic homemade Gadha<br>5. All of the above<br>6. Not any<br>7. Others	*Multi response.*<br>*If Ans: is not any, then jump to 87*
86	If yes, specify the duration (no of weeks) and Dosage.		
87	Status of during pregnancy	1. Not vaccinated<br>2. One dose<br>3. Two doses<br>4. Two doses + precautionary	

Confidential: For Review Only

88	Status of covid vaccination on the date of interview (current)	5. Not vaccinated<br>6. One dose<br>7. Two doses<br>8. Two doses + precautionary	
89	Date & Type of the first vaccine	1. Date_____<br>2. Type_____	
90	Date & Type of the second vaccine	1. Date_____<br>2. Type______	
91	Date & Type of Precautionary Vaccine	1. Date______<br>2. Type______	
92	Did you receive covid-19 counselling during pregnancy?	1. No<br>2. Yes<br>3. Not applicable (no guideline introduced at that time)	*If Ans: is no any, then end the sec:3 here*
93	if yes, where did you receive the counselling?	1. Any Hospital settings (PHC/CHC/SC/private clinic)<br>2. Mamta day<br>3. Mass media (radio, tv,)<br>4. Family/friends<br>5. Other (write)	
94	If yes, did the counselling help you to understand the covid-19 disease, vaccination, misinformation, and its impact on your pregnancy and baby?	1. Yes – It was helped me to develop good understanding<br>2. yes – in somewhat aspects<br>3. yes – little bit but still I have/had many doubts<br>4. Others	

Section 5: Status of Covid Infection during pregnancy and Management details

No	Question	Response	Instruction
95	Have you been tested positive for COVID 19 during your pregnancy?	0. No<br>1. Yes	
96	If yes, during which wave it was detected?	1. 1.1st wave- (March 2020 to November 2020)<br>2. 2.2nd wave- (February 2021 to June 2021)<br>3. 3.3rd wave- (November 2021 to March 2022)	Skip Q:96 to 117 if the answer of ques. 95 is No
97	If yes, record the performed confirmatory investigations and result	Test done | Result (0/1) | Date<br>RAT | | RTPCR | | others | |	
98	If yes, in which trimester of pregnancy?	______month	The First Trimester – (0-13 weeks)<br>The Second Trimester –(14-26weeks)<br>Third Trimester (27 -40)
99	If yes, record the experienced symptoms	1. No symptoms<br>2. Mild Fever<br>3. High grade fever<br>4. Cough<br>5. Cold<br>6. Headache<br>7. Myalgia<br>8. Diarrhea<br>9. Breathlessness<br>10. Loss of test/ smell<br>11. Any other: ________	

Confidential: For Review Only

No	Question	Response	Instruction
100	Mode of treatment during covid	1. Home isolation<br>2. Hospitalization<br>3. ICU care<br>4. Any other______	
101	If home Isolation, no of days	-------------------------Days	Skip Q:101 if the ans. Of Q:100 is other than home isolation
102	If hospitalized, no of days	-------------------------Days	Skip Q:102 if the ans. Of Q:100 is other than hospitalization
103	If admitted to ICU, no of days	-------------------------Days	Skip Q:103 if the ans. Of Q:100 is other than ICU care
104	Level of SPO2	_______________	
105	H/O oxygen support	0. No<br>1. Yes<br>2. NA	
106	If yes, no of days	________	Skip Q:106 if the ans. Of Q:105 is other than yes
107	H/O Steroid during covid treatment	0. No<br>1. Yes	
108	H/O Ventilator during covid treatment	0. No<br>1. Yes	
109	Type of facility for covid treatment	1. Public facility<br>2. Private facility – Physician<br>3. Private facility -BAMS/BHMS<br>4. Trust hospital<br>5. Self-medication<br>6. Any other_______	
110	Latest prescription or pharmacy bill of COVID treatment (Take picture/Choose image)		
111	lab report details	1. Not performed any<br>2. CRP<br>3. D-Dimer<br>4. HB<br>5. ACE-2<br>6. IL-6<br>7. Any Other________<br>8. Performed but not having any record	Take maximum – min value
112	If performed any, record the details for each with min two values (max, min) and the date		Skip Q:112 if the ans. Of Q:111 is opt. 8 & 1
113	Have you taken any medication after recovering from covid? (Covid follow-up RX and name of the medicine)	0. No<br>1. Yes	
114	If yes, record the detail of the treatment	______	Skip Q:114 if the ans. Of Q:113 is No
115	Have you experienced any pregnancy-related complications during/after covid treatment?	0. No<br>1. Yes	
116	If yes, name the complication.		Skip Q:116-40 if the ans. Of Q:115 is No

Confidential: For Review Only

No	Question	Response	Instruction
117	If yes, how did you manage this complication?	1. Hospitalized<br>2. Consulted doctor<br>3. Self-medications<br>4. Any other	Skip Q:117-40 if the ans. Of Q:115 is No
118	Remarks	________	

Section 6: Reproductive History (previous) & health care access during pregnancy (consider pandemic pregnancy)

No	Question	Answer	Instruction
119	Status of Gravida	1. Nulliparous<br>2. Multi para	
120	Record the details (in numbers) of	1. Gravida___<br>2. Parity____<br>3. Abortion ___<br>4. Live births___<br>5. Pre-term birth______	
121	Status of (Covid pandemic pregnancy) on the date of interview	1. Delivered<br>2. Yet to deliver	
122	Date of LMP	__________(dd/mm/year)	
123	Date of EDD	__________(dd/mm/year)	End the tool here if baby is yet to delivered
124	If delivered, outcome of pregnancy	0. Singleton<br>1. Twins<br>2. Triplet<br>3. Any other	
125	How many ANC visit you have completed?	1. Less than 4<br>2. Minimum 4 as per national guideline<br>3. More than 4	
126	Record the reasons if less than 4	_______	
127	Record the reasons if more than 4		
128	What services did you receive at the ANC?	1. BP check<br>2. Hb Check<br>3. Glucose check<br>4. Ultrasound check<br>5. Heart monitor<br>6. Urine analysis<br>7. Lab tasting<br>8. Anthropometry<br>9. Other, pls specify<br>0. Not any	Multiple response is possible
129	What intervention did you receive at your ANC?	1. No Intervention<br>2. Iron -Folic acid tablet<br>3. Calcium tablet<br>4. multi-micronutrient<br>5. De-worming tab<br>6. Counselling for birth planning	Multiple response is possible

Confidential: For Review Only

No	Question	Answer	Instruction
		7. Others pls specify	
130	Have you experienced any complication during pregnancy?	0. No<br>1. Yes	
131	If yes,	1. Frequent contraction<br>2. Vaginal bleeding<br>3. Vaginal discharge<br>4. Gestational diabetes<br>5. Gestational Hypertension<br>6. sudden hike of Blood pressure<br>7. Convulsion<br>8. Anaemia<br>9. Severe Headache<br>10. Any other<br>11. Not any	
132	Have you experienced any complication during labour?	1. Prolonged labour pain<br>2. Excessive vaginal bleeding<br>3. No cervix dilatation<br>4. Convulsion<br>5. premature rupture of membrane (PROM)<br>6. Hike blood pressure<br>7. Vaginal discharge<br>8. Any other_______<br>9. Not any	
133	Have you experienced any complication after labour (during 42 days of delivery)?	1. Excessive vaginal bleeding<br>2. Fever<br>3. Vaginal discharge<br>4. Convulsion<br>5. Vaginal ulcer<br>6. Not any<br>7. Any other	
134	Actual Date of Delivery	__________(dd/mm/year)	Only for mothers who have delivered
135	Place of delivery	1. Home<br>2. Private institution<br>3. Public institution<br>4. Trust hospital<br>5. Any other	
136	Type of delivery	1. Vaginal<br>2. C-section<br>3. Induced labour	
137	If C-section	1. Medical indication<br>2. Elective	
138	If Medical indication, record the details		
139	In which trimester/term the baby was borne?	1. Full term<br>2. Preterm	
140		0. No	

Confidential: For Review Only

No	Question	Answer	Instruction
	Were you delivered at the same place (health care facility) where you received the majority of your ANC services?	1. Yes	
141	If, No Record the details (reasons, no of hospital approached and shifted)	____________	
142	Remarks		

Section:6 Maternal Mental Health during the Pandemic

No	Question	Response	Instructions
143	Do you feel the pandemic has affected your mental health?	1. No<br>2. Yes	
144	What impact has the pandemic had on your mental health?	1. Feeling nervous, anxious, or on edge<br>2. Not being able to stop or control worrying/ Worrying too much about different things<br>3. Being so restless that it is hard to sit still<br>4. Becoming easily annoyed or irritable<br>5. Feeling afraid, as if something awful might happen<br>6. Difficulty falling / staying asleep<br>7. Inadequate / not satisfied sleep - less than 7 hours<br>8. Sleeping too much<br>9. Feeling down, depressed, or hopeless<br>10. Feeling tired or having little energy<br>11. Poor appetite or overeating<br>12. Feeling bad about yourself or that you are a failure or have let yourself or your family<br>13. Trouble concentrating on things, such as reading the newspaper or watching television.<br>14. Moving or speaking so slowly that other people could have noticed. Or the opposite being so get or restless that you have been moving around a lot more than usual<br>15. Thoughts that you would be better off dead, or of hurting yourself<br>16. Stressed<br>17. Other	
145	If any other, record the detail	______________________	
146	If feeling nervous, anxious, or on edge - rank it using Scale 1 to 5 (1 is the lowest & 5 is the highest)		
147	If not being able to stop or control worrying - rank it using Scale 1 to 5 (1 is the lowest & 5 is the highest)		
148	If being so restless that it is hard to sit still - rank it using Scale 1 to 5 (1 is the lowest & 5 is the highest)		
149	If becoming easily annoyed or irritable - rank it using Scale 1 to 5 (1 is the lowest & 5 is the highest)		
150	If feeling afraid, as if something awful might happen - rank it using Scale 1 to 5 (1 is the lowest & 5 is the highest)		

Confidential: For Review Only

No	Question	Response	Instructions
151	If difficulty falling/staying asleep - rank it using Scale 1 to 5 (1 is the lowest & 5 is the highest)		
152	If problems waking up too early / couldn't complete 7 hours sleep - rank it using Scale 1 to 5 (1 is the lowest & 5 is the highest)		
153	If feeling down, depressed, or hopeless- rank it using Scale 1 to 5 (1 is the lowest & 5 is the highest)		
154	If feeling tired or having little energy - rank it using Scale 1 to 5 (1 is the lowest & 5 is the highest)		
155	If poor appetite or overeating - rank it using Scale 1 to 5 (1 is the lowest & 5 is the highest)		
156	If feeling bad about yourself or that you are a failure or have let yourself or your family down- rank it using Scale 1 to 5 (1 is the lowest & 5 is the highest)		
157	If trouble concentrating on things, such as reading the newspaper or watching television - rank it using Scale 1 to 5 (1 is the lowest & 5 is the highest)		
158	If moving or speaking so slowly that other people could have noticed. Or the opposite being so gety or restless that you have been moving around a lot more than usual - rank it using Scale 1 to 5 (1 is the lowest & 5 is the highest)		
159	If thoughts that you would be better o dead, or of hurting yourself- rank it using Scale 1 to 5 (1 is the lowest & 5 is the highest)		
160	If stressed- rank it using Scale 1 to 5 (1 is the lowest & 5 is the highest)		
161	What do you think were the reasons your mental health was impacted during the pandemic?	1. Bereavement<br>2. Isolation<br>3. loss of income<br>4. fear of not having enough food for your household<br>5. Fear of COVID-19 infection<br>fear of losing your unborn baby<br>6. Fear of losing a family member<br>7. Inability to visit ANC/hospital<br>8. Other (write)	
162	If Other, record the details		
163	Do you currently have or had any mental health disorders before the pandemic?	0. No<br>1. Yes	
164	If yes, record the details using prompt from above Q: what impact has the pandemic had on your mental health?	______________	
165	If yes, Record the reason for present mental health condition	______________	
166	Does anyone in your family currently have or had mental health disorder?	0. No<br>1. Yes	

Confidential: For Review Only

Section:7 Current Food frequency questionnaire

No	Food items	Daily	Four times a week	Thrice a week	Twice a week	Once a week	Once in 15 days	Once in a month	Occasionally /seasonal	Never
**[A]**	**Protein-rich foods**									
1	Bajra									
2	Wheat flour, whole									
3	Bengal gram, dal									
4	Green gram, dal									
5	Lentil, dal									
6	Red gram, dal									
7	Soyabean, white									
8	Groundnut									
9	Egg, poultry, whole, boiled									
10	Chicken, breast									
11	Chicken, liver									
12	Salmon									
13	Tuna									
**[B]**	**Vitamin A and Beta-carotene-rich foods**									
1	Egg, poultry, whole, boiled									
2	Chicken, liver									
3	Goat, liver									
4	Amaranth, leaves									
5	Colocasia, leaves									
6	Drumstick, leaves									
7	Fenugreek leaves									
8	Spinach									
9	Mango, ripe, kesar									
10	Sweet potato, brown skin									
11	Carrot, orange									
**[C]**	**Iron-rich foods**									
1	Bengal gram, whole									
2	Cowpea, brown									
3	Lentil, dal									
4	Moth beans									
5	Peas, dry									
6	Fenugreek, leaves									
7	Drumstick, leaves									
8	Cluster beans									
9	Dates, dry, dark brown									
10	Mint leaves									
11	Gingelly seeds, white									
12	Niger seeds, black									
13	Chicken, liver									
14	Beef, liver									
**[D]**	**Calcium-rich foods**									

Confidential: For Review Only

No	Food items	Daily	Four times a week	Thrice a week	Twice a week	Once a week	Once in 15 days	Once in a month	Occasionally/seasonal	Never
1	Ragi									
2	Bengal gram, whole									
3	Moth beans									
4	Rajmah, brown									
5	Soyabean, brown									
6	Red gram, whole									
7	Amaranth, leaves, green									
8	Colocasia, leaves									
9	Fenugreek, leaves									
10	Drumstick, leaves									
11	Curry leaves									
12	Mint leaves									
13	Gingelly seeds, white									
14	Paneer									
15	Khoa									

Section:8 Nutrition Access during pandemic

No	Question	Response	Instruction
167	What foods do you usually eat (i.e., usual dietary intake/staple foods)? List and describe		See the current FFQ for the details.
168	On average, how many times in a week do you eat these foods?		
169	Was there a time in the pandemic when you were not able to get these foods?	0. No<br>1. Yes<br>2. Don't know	
170	What foods were you mostly unable to get during the pandemic? List and describe		
171	How frequently were the foods not available during the pandemic?	0. Never<br>1. Often<br>2. sometimes	
172	What was the reason?	0. Poverty/low income (chronic)<br>1. Unemployment<br>2. Systemic racism and racial discrimination<br>3. Unavailable near by/in local market<br>4. Unaffordable (so expensive that people do not have enough money to buy it or pay for it)<br>5. Inaccessible (very difficult/impossible to get)<br>6. Others	Where the participant chooses all or first 3 options, ask what she think was the main reason?
173	What was the main reason?	0. Unavailable<br>1. Unaffordable<br>2. Inaccessible<br>3. Others, specify	Where the participant said the foods were unaffordable (i.e., prices increased), ask what the increment were.

No	Question	Response	Instruction
174	What was the price of these foods before the pandemic?		
175	What was the price of these foods during the pandemic?		
176	Was there a time you had to change your dietary habits because the foods you usually eat were unavailable, inaccessible, or too expensive?	0. No<br>1. Yes<br>2. Don't know	
177	What changes in dietary habits did you make?	1. Reduced the quantity of foods I eat<br>2. I reduced the amount meals I eat<br>3. I substituted my usual foods to other types<br>4. I eat when I can<br>5. other	
178	What foods did you eat when the foods you usually eat were not available?		
179	Were you eating a three-square meal a day (i.e., breakfast, lunch and dinner) during pregnancy?	1. Yes-always<br>2. Yes-sometimes<br>3. No	
180	What meals do you almost always (usually) eat during the pandemic?	1. Breakfast, lunch, dinner<br>2. breakfast & lunch<br>3. breakfast & dinner<br>4. lunch & dinner	

Confidential: For Review Only

Longitudinal follow-up of babies born to covid-19 positive antenatal mothers in Gujarat - Children

Basic details	
Unique ID (Children)	☐☐ ☐☐ ☐☐☐ ☐☐
Name of the districts	1. Ahmedabad<br>2. Sabarkatha
Maternal status of covid infection during pregnancy	0. No<br>1. Yes
Date of Interview	
Name of RA	

Section:1 Birth Outcome details

Sr.no	Questions	Response	Instructions
1	Birth date of the child	dd/mm/yyyy	
2	Age of the baby	☐☐ In months	*verbal*
3	Gender	1. Male<br>2. Female	
4	Birth weight	____________Kg	
5	Did the baby cry immediately after birth? (recorded/maternal recall)	0. No<br>1. Yes	
6	Did respiration rate normal immediately after birth? (recorded/maternal recall)	0. No<br>1. Yes	
7	Did the baby's feeding reflexes normal immediately after birth? (recorded/maternal recall)	0. No<br>1. Yes	
8	Did the baby develop COVID-related symptoms (cough, cold, fever, breathlessness) within 24 hours of birth?	0. No<br>1. Yes<br>2. Don't know	
9	If yes, record the detail of whether he/she went for confirmatory investigations & if yes, mention the result	0. No<br>1. Yes	
10	Did the child admitted to NICU/PICU?	0. No<br>1. Yes	

Confidential: For Review Only

Sr.no	Questions	Response	Instructions
11	If yes, the reason for admission		Skip Q:11 if the answer is no in Q:10.
12	Any congenital abnormality in the baby	0. No<br>1. Yes	
13	If yes, please specify		Skip Q:13 if the answer is no in Q:12.

Section:2 Anthropometry & Morbidity Profile

Sr.no	Question	Response	Instruction
14	Current weight in Kg	________kg	
15	Current Height in cm	________cm	
16	Current MUAC in cm	________cm	
17	Head circumference in Cm	________cm	
18	Does the child have any infection within the past seven days?	0. No<br>1. Yes	
19	If yes, record the details.		
20	Present status of 1st baby	0. Alive and healthy<br>1. Alive with some health issue<br>2. Dead	
21	Specify the condition if alive with some health issue (1st baby).	____________	Skip Q:21 if answer 20 is other than opt.1
22	Any other major health complications to the baby after birth?	1. No<br>2. Yes	
23	If yes, record the details.	______________	
24	Any other H/O hospitalization to date?	0. No<br>1. Yes	
25	If yes cause of hospitalization	____________	Skip Q:25 if the answer is no in Q:24.

Confidential: For Review Only

Section:2 IYCF Practice & Immunization details

Sr.no	Question	Response	Instructions
26	Initiation of breastfeeding?	1. within an hour of birth<br>2. within 2-4 hours of birth<br>3. Within 24 hours of birth<br>4. After one day<br>5. Within three days<br>6. any Other_______	
27	Did the baby receive the first yellowish breast milk which came out after birth?		
28	Till what age baby was given only breastmilk?	________months	
29	Age eligibility for immunization on the date of the 1st interview	1. less than six weeks<br>2. 6 weeks<br>3. 10 weeks<br>4. 14 weeks<br>5. 9-12 months<br>6. 16 -24 month	
30	If the child's age is less than six weeks	1. BCG<br>2. OPV-0<br>3. Hepatitis birth dose<br>4. Not any	Multi response
31	If the child's age is up to 6 weeks	1. BCG<br>2. OPV-0<br>3. Hepatitis birth dose<br>4. OPV-1<br>5. PENTAVALENT-1<br>6. FIPV-1<br>7. ROTA-1<br>8. PCV-1<br>9. Not any	Multi response
32	If the child's age is up to 10 weeks	1. BCG<br>2. OPV-0<br>3. Hepatitis birth dose<br>4. OPV-1<br>5. PENTAVALENT-1<br>6. FIPV-1<br>7. ROTA-1<br>8. PCV-1<br>9. OPV-2<br>10. PENTAVALENT-2<br>11. ROTA-2<br>12. Not any	Multi response

Confidential: For Review Only

Sr.no	Question	Response	Instructions
33	If the child's age is up to 14 weeks	1. BCG<br>2. OPV-0<br>3. Hepatitis birth dose<br>4. OPV-1<br>5. PENTAVALENT-1<br>6. FIPV-1<br>7. ROTA-1<br>8. PCV-1<br>9. OPV-2<br>10. PENTAVALENT-2<br>11. ROTA-2<br>12. OPV-3<br>13. PENTAVALENT-3<br>14. FIPV-2<br>15. ROTA-3<br>16. PCV-2<br>17. Not any	Multi response
34	If the child's age is up to 9 - 12 months	1. BCG<br>2. OPV-0<br>3. Hepatitis birth dose<br>4. OPV-1<br>5. PENTAVALENT-1<br>6. FIPV-1<br>7. ROTA-1<br>8. PCV-1<br>9. OPV-2<br>10. PENTAVALENT-2<br>11. ROTA-2<br>12. OPV-3<br>13. PENTAVALENT-3<br>14. FIPV-2<br>15. ROTA-3<br>16. PCV-2<br>17. MR-1<br>18. PCV-BOOSTER<br>19. Not any	Multi response

Confidential: For Review Only

Sr.no	Question	Response	Instructions
35	If the child's age is up to 16-24 months	20. BCG<br>21. OPV-0<br>22. Hepatitis birth dose<br>23. OPV-1<br>24. PENTAVALENT-1<br>25. FIPV-1<br>26. ROTA-1<br>27. PCV-1<br>28. OPV-2<br>29. PENTAVALENT-2<br>30. ROTA-2<br>31. OPV-3<br>32. PENTAVALENT-3<br>33. FIPV-2<br>34. ROTA-3<br>35. PCV-2<br>36. MR-1<br>37. PCV-BOOSTER<br>38. MR-2<br>39. 2. OPV-BOOSTER<br>40. Not any	
36	Age eligibility for Vaccination during a follow-up visit (FU)	1. less than nine months<br>2. 9-12 months - Full Immunization<br>3. 13 -24 months - Complete immunization	
37	Status of immunization for less than nine months (FU)	1. Received all eligible vaccines with the alignment of the national immunization schedule<br>2. Received but not as per schedule<br>3. Not received all eligible vaccine<br>4. Record not found	PCV is applicable to children born after 20th October 2021 in Gujarat; PCV was introduced on 20th October 2021
38	If received but not as per schedule, record the reasons. (1 FU)	__________	
39	If not received all eligible vaccines/missing some, record the reasons. (FU)		
40	Status of immunization for 9-12 months (a child is eligible for Full Immunization) (1st FU)	1. Received all eligible vaccines with the alignment of the national immunization schedule<br>2. Received but not as per schedule<br>3. Not received all eligible vaccine<br>4. Record not found	Full Immunization: One dose of BCG, Measles/MR/MMR, three doses of OPV, Pentavalent, Rotavirus and PCV, and two doses of IPV.
41	If received but not as per schedule, record the reasons. (1st FU)	_____________	

Confidential: For Review Only

Sr.no	Question	Response	Instructions
42	If not received all eligible vaccines/missing some, record the reasons. (1st FU)	___________	
43	Status of immunization for 13-24 months & above (Complete immunization) (1st FU)	0. Received all eligible vaccines with the alignment of the national immunization schedule<br>1. Received but not as per schedule<br>2. Not received all eligible vaccine Record not found	Complete Immunization: All vaccines described in Q:40 + Second dose of Measles/ MR/MMR, and one booster dose of OPV and DPT
44	If received but not as per schedule, record the reasons. (1st FU)	___________	
45	Consent for a biochemical marker?	1. For mother only<br>2. For baby only<br>3. For mother and baby both<br>4. Will let us know after discussion with family<br>5. Not for any	

Section: 3 Food Frequency Questionnaire

No	Food items	Daily	Four /week	Thrice/ week	Twice/ week	Once/ week	Once/ 15 days	Once/ month	Occasionally /seasonal	Never
**[A]**	**Protein-rich foods**									
1	Bajra									
2	Wheat flour, whole									
3	Bengal gram, dal									
4	Green gram, dal									
5	Lentil, dal									
6	Red gram, dal									
7	Soyabean, white									
8	Groundnut									
9	Egg, poultry, whole, boiled									
10	Chicken, breast									
11	Chicken, liver									
12	Salmon									
13	Tuna									
**[B]**	**Vitamin A and Beta-carotene-rich foods**									

Confidential: For Review Only

No	Food items	Daily	Four /week	Thrice/ week	Twice/ week	Once/ week	Once/ 15 days	Once/ month	Occasionally /seasonal	Never
1	Egg, poultry, whole, boiled									
2	Chicken, liver									
3	Goat, liver									
4	Amaranth, leaves									
5	Colocasia, leaves									
6	Drumstick, leaves									
7	Fenugreek leaves									
8	Spinach									
9	Mango, ripe, kesar									
10	Sweet potato, brown skin									
11	Carrot, orange									
**[C]**	**Iron-rich foods**									
1	Bengal gram, whole									
2	Cowpea, brown									
3	Lentil, dal									
4	Moth beans									
5	Peas, dry									
6	Fenugreek, leaves									
7	Drumstick, leaves									
8	Cluster beans									
9	Dates, dry, dark brown									
10	Mint leaves									
11	Gingelly seeds, white									
12	Niger seeds, black									
13	Chicken, liver									
14	Beef, liver									
**[D]**	**Calcium-rich foods**									
1	Ragi									
2	Bengal gram, whole									
3	Moth beans									
4	Rajmah, brown									

Confidential: For Review Only

No	Food items	Daily	Four /week	Thrice/ week	Twice/ week	Once/ week	Once/ 15 days	Once/ month	Occasionally /seasonal	Never
5	Soyabean, brown									
6	Red gram, whole									
7	Amaranth, leaves, green									
8	Colocasia, leaves									
9	Fenugreek, leaves									
10	Drumstick, leaves									
11	Curry leaves									
12	Mint leaves									
13	Gingelly seeds, white									
14	Paneer									
15	Khoa									

Section: 4 ECD questionnaire

Sr.no	Question	Response	Instructions
1	Birth date	dd/mm/yyyy	
2	Assessment date	dd/mm/yyyy	
3	Age eligibility for ECD	1. INTER-NDA<br>2. OX-NDA<br>3. Not applicable	
4	Status of ECD data collection	0. Not Collected<br>1. Collected	
5	If not collected, record the reason.		
7	Relationship of carer to the child		
8	What is the child's native (first) language?		
9	Does the child speak/understand any language other than his/her nativelanguage?		
10	Is the child exposed to any language other than his/her native languagefor more than 30 minutes each day on most days?		
11	What is the language in which the assessment is being conducted?		

Confidential: For Review Only

The INTERGROWTH-21st Project Neurodevelopment Assessment - The INTER-NDA

No	Item	Observed Performance				
1	**Builds a tower of 5 cubes** *(trials=3, demonstration=3)*	5 cubes	3-4 cubes	2 cubes	No attempt	Unable to assess
2	**Names 4 colours when asked to do so** *(trials=1, demonstration=0)*	Names 4 colours	Names 3 colours	Names 1 or 2 colors	Does not name anycolor	Unable to assess
3	**Matches 3 cubes of same colours when requested todo so** (trials=1, demonstration=1 of one colour)	Matches 3 colours	Matches 2 colours	Matches 1 colour	Does not match any colour	Unable to assess
4	**Hands the examiner one cube when asked to do so (Examiner says "Please give me one cube" & keeps palm open for 5 seconds after child has handed over1 cube)** *(trials=1, demonstration=0)*	Hands only oneblock within 5 seconds	Hands only one block in more than 5 seconds	Hands two or more blocks	Does not handany block / No attempt	Unable to assess
5	**Puts the spoon in the cup when asked to do so** *(trials=5, demonstration=0)*	Puts the spoon in cup in ≤3 trials	Puts the spoon in cup in 4-5 trials	Takes the spoon or the cup but does not complete action	No attempt	Unable to assess
6	**Matches shapes on board** *(trials=5, demonstration=partial – removal only)*	All shapes in ≤3 trials	All shapes with repeated demonstration i.e. 4-5 trials	One or two shapesin 4-5 trials	No attempt	Unable to assess

Confidential: For Review Only

7	**Matches shapes on rotated board**<br>*(trials=5, demonstration=partial – removal only)*	All shapes in ≤3 trials	All shapes with repeated demonstration i.e. 4-5 trials	One or two shapesin 4-5 trials	No attempt	Unable to assess

8	**Points correctly when asked "Where is thedoor/entrance to the room?"**<br>*(trials=5, demonstration=0)*	Identifies door correctly in ≤3 trials	Identifies door correctly in 4-5 trials	Attempts, but doesnot identify door	No attempt	Unable to assess
9	**Puts a raisin precisely inside a small opening in abottle**<br>*(trials=1, demonstration=1, test both hands)*	Precise release of raisin into bottle witheach hand	Clumsy release, raisin falls out of bottle with one ormore hand	Attempts but unsuccessful release with one ormore hand	No attempt	Unable to assess
10	**Drinks water from cup/bottle/sippy cup when placedin front of child**<br>*(trials=1, demonstration=0; maternal recall if observationnot possible)*	Drinks water fromcup/sippy cup without spilling	Drinks clumsily & spills	Attempts but unsuccess ful	No attempt	Unable to assess
11	**Looks towards an object located across the roomwhen pointed at by the examiner**<br>(trials=5)	Looks or points atobject in ≤3 trials	Looks or points atobject in 4-5 trials	Looks at the wrongobject, or attempts but cannot identify object	No attempt	Unable to assess
12	**Pretends to drink from a toy cup when placed in frontof him/her**<br>*(trials=2, demonstration=1 if not spontaneous on firstattempt)*	Spontaneously	After 1 demonstrati on	Partial attempt after1 demonstratio	No attempt	Unable to assess

				n		
13	**Able to make a cup of tea with the toy tea set whenrequested by examiner (Examiner says "Can you make a cup of tea?")** *(trials=2, demonstration=1 if not spontaneous on firstattempt)*	Spontaneously, withpouring motion	After 1 demonstrati on	Partial attempt after1 demonstratio n	No attempt	Unable to assess
14	**Feeds doll when requested to (Examiner says "Canyou give the dolly some tea?")** *(trials=2, demonstration=1 if not spontaneous on firstattempt)*	Spontaneously	After 1 demonstrati on	Partial attempt after1 demonstratio n	No attempt	Unable to assess
15	**Imitates straight horizontal scribble** *(trials=5, demonstration=5)*	≤3 trials	4-5 trials; with difficulty	Attempts (hold crayon)	Cannot hold crayon	Unable to assess
16	**Identifies glitter bracelet under correct washcloth** *(trials=5, demonstration=0, test both sides)*	Finds bracelet correctly in ≤2 trailson both sides	Find bracelet correctly in 3 trials or on one side only	Find bracelet correctly in 4-5 trialsor on one side only	Does not find bracelet or noattempt	Unable to assess
17	**Correctly identifies object groups using plurals** *(concurrent observation)*	Uses 5 plurals	Uses 3-4 plurals	Uses 1-2 plurals	Does not useany plurals	Unable to assess

18	**Asks for toilet by gesture or verbally** *(maternal recall)*	Always	Occasionally	Partial (only for bowel movement)	Never	Unable to assess

Confidential: For Review Only

19	**Runs**<br>*(maternal recall)*	Runs steadily	Attempts	Walks only	Walks with suppor t	Unable to assess
20	**Throws a ball very near**<br>*(trials=1, demonstration=1; test both hands)*	Good release	Unsteady release	Attempts	No attempt	Unable to assess
21	**Kicks ball**<br>*(maternal recall)*	Kicks ball with kneeflexed	Runs after ball & attempts kicking it	Walks and touchesball with foot	No attempt	Unable to assess
22	**Climbs upstairs holding rail, 2 feet/stair or in adultfashion**<br>*(maternal recall)*	Climbs stairs alone steadily	Climbs stairs alone unsteadily	Climbs stairs with help (uses railing, holds adult's hand)	No attempt	Unable to assess
23	**Uses 2-4 syllable babble such as dada, mama but notspecifically to anything or any person**<br>*(concurrent observation)*	Spontaneously	Mimics	1 syllabe babble e.g. ba, ma, da	None	Unable to assess
24	**Use two words together**<br>*(concurrent observation)*	Two words, appropriate use	Two words, inappropriate use	One word, appropriate use	No attempt	Unable to assess
25	**Indicates by gesture to say no**<br>*(concurrent observation or maternal recall)*	Indicates verbally orby definite gesture all the time	Indicates verbally orby definite gesture some of the time	Attempts, but incomplet e indication	No attempt	Unable to assess
26	**Use of a pronoun e.g. me, my, she, he, it, I**<br>*(concurrent observation)*	≥1 pronoun incorrect	≥1 pronoun,	Use of proper names but	No use	Unable to

Confidential: For Review Only

		context	incorrect use	not pronouns		assess
27	**How many words does the child use during theassessment other than mama/dada** *(concurrent observation)*	≥8 words	6-7 words	4-5 words	≤3 words	Unable to assess
28	**How many sentences of 3 words or more does thechild use during the assessment?** *(concurrent observation)*	≥2	1	≥1 two word utteranc e	None	Unable to assess
29	**In how many instances does the child follow on atopic of conversation providing new information?** *(concurrent observation)*	At least one, using ≥2 words, proving correct information	At least one, uses single words, provides correct information	Uses any number of words, provides incorrect information	Does not follow up on conversati ons	Unable to assess

31	**Positive Affect**	Never or rarely	Some of the time	Most of the time
32	**Exploration**	Never or rarely	Some of the time	Most of the time
33	**Ease of engagement**	Never or rarely	Some of the time	Most of the time
34	**Cooperativeness**	Never or rarely	Some of the time	Most of the time
35	**Adaptability to change**	Never or rarely	Some of the time	Most of the time
36	**Distractibility**	Never or rarely	Some of the time	Most of the time
37	**Negative Affect**	Never or rarely	Some of the time	Most of the time

Confidential: For Review Only

The Oxford Neurodevelopment Assessment for 10 – 14 months

Item No	Item	Observed Performance				
1	Builds a tower of 3 cubes *(Trials = 5, demonstration = 5)*	3 cubes in ≤3 trials	3 cubes in 4-5 trials	2 cubes in 5 trials	No attempt	Unable to assess
2	Takes 3 cubes out of cup (Examiner says "Look, I am putting the cubes into the cup – one, two and three. Can you take them out?") *(Trials =3, demonstration = 0 but visibly place cubes inside cup before asking child to take them out counting as you go)*	Takes all 3 cubes out of the cup in ≤3trials	Takes 2 cubes out of cup	Takes 1 cube out of cup	No attempt	Unableto assess
3	Puts 3 cubes back in cup (Examiner says 'Can you put the cubes back in the cup?") *(Trials = 3, demonstration = 0)*	Puts all 3 cubes inside cup in ≤3 trials	Puts 2 cubes inside cup in ≤3 trials	Puts 1 cube inside cup in ≤3 trials	No attempt	Unableto assess
4	Hands the examiner one cube when asked to do so (Examiner says 'Please can you give me 1 cube?") *(Trials = 1, demonstration = 0)*	Precise release of one cube into examiner's hand	Clumsy release, cube falls out of examiner's or child's hand, childgives examiner more than one cube	Attempts but unsuccessful release with oneor more hand	No attempt	Unableto assess

Confidential: For Review Only

5	Squeeze toy to make sound (trials = 1, demonstration = 2 squeezes followed by 2 squeezes)	Squeezes toy to make sound	Squeezes toy but not hard enough to make sound	Picks up toy and plays with it but does not squeeze it	No attempt	Unable to assess
6	Drop squeeze toy on floor (Examiner says "Uh oh, where's ducky gone?") (trials = 5 following verbal prompts, demonstration = 0)	Child looks towards object or tries to retrieves it spontaneously	Child looks towards object or tries to retrieve it in ≤3 prompts	Child starts looking for fallen toy on repeated prompting (4-5 prompts)	Child does not look for toy even on repeated prompting	Unable to assess
7	Finds squeeze toy under cup (Examiner hides toy under cup and says 'Where's ducky gone now?") (trials = 1, demonstration = 0; verbal prompts = maximum 3)	Child retrieves toy spontaneously by inverting cup	Child pushes cup or points to cup but does not retrieve toy	Child looks at cup but does not touch it	No attempt	Unable to assess
8	Ask child to give squeeze toy to mummy (trials = 5, demonstration = 0)	Child gives toy to mother in in ≤3 trials	Child gives toy to mother with repeated prompting i.e. 4-5 trials	Child does not give toy to mother or gives it and takes it back	No attempt	Unable to assess

Confidential: For Review Only

9	Identifies spoon out of 5 objects. Examiner says, "Which one is the spoon?"<br>(trials = 5, demonstration = 0)	Child correctly identifies spoon in ≤3 trials	Child identifies spoon with repeated prompting i.e. 4-5 trials	Child identifies an object but it is not the spoon	No attempt	Unable to asses
10	Identifies sock and toothbrush out of 5 objects. Examiner says, "Show me the sock and the toothbrush".<br>(trials = 3, demonstration = 0)	Child correctly identifies sock and toothbrush correctly	Child identifies one object correctly	Child attempts to identify objects but none are correct	No attempt	Unable to asses
11	Pretends to drink from a toy tea cup when placed in front of him/her (Trials = 2, demonstration = 1 if not spontaneous<br>on first attempt)	Spontaneously	After 1 demonstration	Partial attempt after 1 demonstration	No attempt	Unable To assess
12	Lifts empty cup by handle<br>(if not concurrently observed with item above; demonstration=1, trial = 1)	Lifts cup to mouth by handle using one hand with pincer grasp	Lifts cup to mouth using one hand with full hand grasp	Lifts cup to mouth using both hands – full grasp	Touches cup but does not lift it or no attempt	Unable to assess

Confidential: For Review Only

13	Feeds doll when requested to. Examiner says, "Can you feed dolly some tea?"<br>(Trials = 2, demonstration = 1 if not spontaneous on first attempt)	Spontaneously	After 1 demonstration	Partial attempt after 1 demonstration	No attempt	Unable to assess
14	Matches shapes in puzzle (objects in 1 line)<br>(Trials = 5, demonstration = 0)	All shapes in ≤3 trials	All shapes in 4-5 trials	1 or 2 shapes	No attempt	Unable to asses
15	Matches shapes on rotated puzzle (objects in 1 line)<br>(Trials = 5, demonstration = 0)	All shapes in ≤3 trials	All shapes in 4-5 trials	1 or 2 shapes	No attempt	Unable to asses
16	Unscrews lid of box to retrieve raisins<br>(Trials = 2, demonstration=1 if first attempt unsuccessful)	Unscrews lid in first attempt	Unscrews lid after demonstration	Attempts but unsuccessful	No attempt	Unable to asses

Confidential: For Review Only

17	Puts a raisin precisely inside a small opening in a box (Trials =1, demonstration = 1, test both hands)	Precise release of raisin into box with each hand	Clumsy release, raisin falls out of bottle with one or more hand	Attempts but unsuccessful release with one or more hand	No attempt	Unable to assess
18	Thumb-finger tip grasp – grasps raisin between thumb and index finger (Observed during item above, trials = 0, demonstration = 0)	Precisely grasps raisin between thumb & index finger on both	Clumsily grasps raisin between thumb & index finger on either	Grasps the raisin with fingers on one or both hands	Tries to grasp raisin with palm or no attempt	Unable to assess
		hands	hand or both hands			
19	Child imitates 4 different consonant-vowel combinations e.g. ‘dolly’ ‘baby’ ‘lorry’ ‘happy’ ‘cookie’ (Trials = 4; can ask child to repeat after mother)	Imitates 3-4 consonant-vowel combinations correctly	Imitates 1-2 consonant-vowel combinations correctly	Imitates but not correctly	No attempt	Unable to assess

Confidential: For Review Only

20	Responds to name by interrupting activity (scribbling)<br>(Trials = 3, demonstration = 0)	Immediately on calling name when engaged in an activity	Only when mother calls child's name when child is involved in an activity	On repeated calling i.e. 2-3 trials	Does not respond to name	Unable to assess
21	Responds to 'no-no' e.g. stops reaching for an object (more crayons) when you say no-no (Trials = 3, demonstration = 0)	Child stops immediately (may or may not continue reaching after a few seconds)	Child stops on repeating no-no 2- 3 times (may or may not continue reaching after a few seconds)	Child does not stop reaching but looks up	Child does not react to no-no	Unable to assess
22	Transfers ball from one hand to another (Trials =1, demonstration = 1)	Freely transfers ball from right to left and left to right hands	Transfers in one direction only	Unable to release ball during 1 or more transfers	No attempt	Unable to assess
23	Walks sideways with support<br>(Concurrent observation or maternal recall)	Walks 3-4 steps with stepping movements	Walks 1-2 steps with stepping movements	Stands only (with/without support)	Stands momentaril y or no attempt	Unable to assess

Confidential: For Review Only

24	Stands alone for >3 seconds when placed in that position (Concurrent observation/trials = 1)	Stands without support for >3 seconds	Stands without support for <1-3 seconds and then falls/reaches for support	Attempts but cannot stand at all without support	No attempt	Unable to assess
25	Walks alone (Concurrent observation/maternal recall)	Walks 5 steps unsupported with stepping movements	Walks 1-3 steps unsupported with stepping movements	Walks forward supported by an adult	Cannot walk even if supported	Unable to assess
26	Raises self from sitting to standing position (Concurrent observation/maternal recall)	Can stand up himself/herself with support and stay standing for 5 seconds (chair/table/adult's hand)	Can stand up himself/herself with support but cannot stay standing for 5 seconds (chair/table/adu lt's hand)	Tries to stand up with support but not able to stand up completely	Cannot stand up from sitting position at all,– or no attempt	Unable to assess
27	Two 2-4 syllable babble such as dada, mama but not specifically to any person or any thing (Concurrent observation)	Spontaneously	Mimics	1 syllable bable e.g. ma, da, pa	None	Unable to assess

Confidential: For Review Only

28	Uses one meaningful word (Concurrent observation)	Spontaneously, and in correct context	Spontaneously, but in incorrect context	Mimics on hearing	Does not use words	Unable to assess
29	Uses two meaningful words together (Concurrent observation)	Spontaneously, and in correct context	Spontaneously, but in incorrect context	Mimics on hearing	Does not use words	Unable to assess
30	Combines word and gesture when asked (Do not demonstrate, trials = 3, use different examples each time)	Combines word and gesture completely and appropriately	Combines word and gesture completely but inappropriately	Combines word and gesture incompletely but inappropriately	Does not combine word and gesture	Unable to assess
31	Positive affect		Never or rarely	Some of the time	Most of the time	
32	Exploration		Never or rarely	Some of the time	Most of the time	
33	Ease of engagement		Never or rarely	Some of the time	Most of the time	
34	Cooperativeness		Never or rarely	Some of the time	Most of the time	
35	Adaptability to change		Never or rarely	Some of the time	Most of the time	
36	Distractibility		Never or rarely	Some of the time	Most of the time	
37	Negative Affect		Never or rarely	Some of the time	Most of the time	

Confidential: For Review Only
